# Data-driven multi-objective analysis of activity–poisoning resistance trade-offs in complex multimetallic electrocatalysts for glucose oxidation

**DOI:** 10.1039/d6ey00174b

**Published:** 2026-11-05

**Authors:** Divyansh Gautam, Ahmad Tirmidzi, Sarvada Shailesh Andhare, Nicolas Schlegel, Armin Asghari Alamdari, Jonathan Quinson, Gustav K. H. Wiberg, Matthias Arenz

**Affiliations:** a Department of Chemistry, Biochemistry and Pharmaceutical Sciences, University of Bern, Freiestrasse 3 CH-3012 Bern Switzerland matthias.arenz@unibe.ch; b Metallurgical Engineering and Material Science, Indian Institute of Technology Bombay, Powai 400076 Mumbai Maharashtra India; c Department of Chemistry, University of Copenhagen, Universitetsparken 5 DK-2100 Copenhagen Denmark; d Biological and Chemical Engineering Department, Aarhus University, 40 Åbogade 8200 Aarhus Denmark; e CICA-Centro Interdisciplinar de Química e Bioloxía, Facultade de Ciencias, Universidade da Coruña, Campus de Elviña A Coruña Spain; f Department of Physics, University of Nevada, Reno 1664 N. Virginia Street Reno NV 89557 USA

## Abstract

The discovery of efficient and durable multimetallic nanoparticle electrocatalysts requires effective navigation of vast compositional spaces. However, systematically exploring these spaces while balancing competing performance objectives remains challenging. Here, we present a closed-loop experimental framework for medium-throughput screening and multi-objective analysis of the trade-off between “activity” (nanoparticle-coverage-normalized current density) and “poisoning resistance” (current retention ratio) for the electrochemical glucose oxidation reaction (GOR). The framework couples electrochemical nanoparticle synthesis, performance evaluation, materials characterization, and machine learning to systematically explore the six-element Au–Ir–Pt–Pd–Rh–Ru composition space. Nanoparticles with controlled compositions were synthesized *via* our recently developed electrodeposition approach and evaluated in terms of nanoparticle-coverage-normalized current density and current retention ratio during GOR under alkaline conditions. The experimental data were used to train Gaussian process regression (GPR) models, which reveal distinct performance landscapes and highlight a trade-off between the two objectives, “activity” and “poisoning resistance”. Highly active compositions are localized near the Au–Pt bimetallic edge with minor contributions from Ir, Pd, Rh, and Ru. In contrast, the most poisoning-resistant compositions were found toward the center of the composition space with contributions from Rh and Pd up to 20 at%. Model interpretation using SHapley Additive exPlanations (SHAP) attributes Au and Pt as the primary contributors to both activity and poisoning resistance, while Rh and Pd contribute additionally to poisoning resistance. Overall, this work establishes a generalizable, data-driven strategy for the discovery of complex multimetallic electrocatalysts.

## Abstract 2

Experiment-guided Bayesian optimization and Pareto analysis identifies Au–Pt-rich candidates balancing activity and current retention within the Au–Ir–Pt–Pd–Rh–Ru nanoparticle composition space.

## Graphical abstract

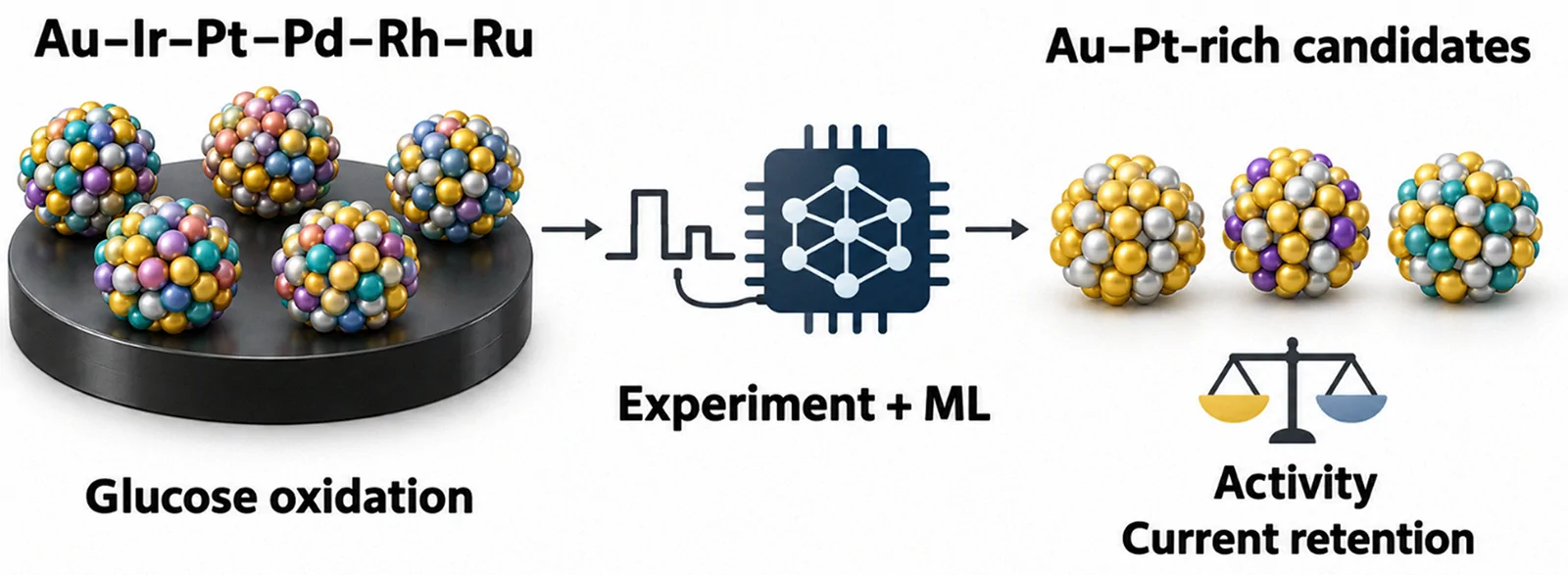

**Broader context**

Discovering catalysts that are both highly active and resistant to performance loss is a central bottleneck in electrochemical energy and sensing technologies, from fuel-forming reactions to biosensors. Multimetallic nanoparticles, *i.e.*, alloys combining several metals, offer a vast space of possible compositions in which to find such catalysts, but this space is far too large to explore by trial and error, and activity and durability often work against each other. This work presents a closed-loop, data-driven strategy that combines rapid nanoparticle synthesis, automated electrochemical testing, and machine learning to navigate a six-metal composition space for the electrochemical oxidation of glucose, a reaction relevant to diabetes-sensing technology and to converting biomass-derived sugars into valuable chemicals. By explicitly modeling the trade-off between activity and resistance to catalyst poisoning, the approach identifies multimetallic compositions that balance both properties–candidates a purely activity-driven search would miss. The workflow itself is not reaction-specific and offers a general route to accelerating the discovery of complex catalysts for other energy-relevant electrochemical transformations.

## Introduction

Catalyst optimization remains a time- and resource-intensive task. This challenge is well illustrated by the historical development of ammonia synthesis catalysts, during which Alwin Mittasch conducted one of the earliest systematic catalyst-screening campaigns, evaluating approximately 3000 catalysts in the search for an industrially viable material.^1,2^ Despite substantial advances in our understanding of surface chemistry and reaction mechanisms, catalyst discovery still often relies on empirical, trial-and-error approaches guided by chemical intuition.^3,4^

In recent years, high- and medium-throughput experimentation combined with machine learning has emerged as a powerful alternative to empirical catalyst discovery.^5–7^ In this context, compositionally complex materials such as high-entropy alloys (HEAs) have attracted significant attention as platforms for catalyst discovery in electrocatalysis.^8–11^ In HEAs, several principal elements are mixed, ideally forming a single-phase solid solution, creating a broad distribution of adsorption sites. Varying the composition enables continuous tuning of catalytic properties, making compositionally complex materials such as HEAs particularly attractive for systematic exploration.^12–16^ Thin-film combinatorial libraries fabricated by gradient magnetron sputtering have demonstrated the effectiveness of this concept, allowing automated electrochemical screening of hundreds of compositions on a single substrate.^9,17^

Implementing analogous strategies for nanoparticles is considerably more challenging because medium- and high-throughput studies require the rapid, reproducible synthesis of particles with precisely controlled compositions.^18^ Consequently, only a limited number of studies have explored compositional screening of complex multimetallic nanoparticles, particularly when multiple performance objectives must be optimized simultaneously.

Recently, we introduced an electrodeposition-based synthesis approach that enables the preparation of noble-metal-based compositionally complex nanoparticles in the Au–Ir–Pt–Pd–Rh–Ru composition space in a medium-throughput approach.^19^ This method is rapid, experimentally straightforward, and relies solely on metal precursors in aqueous electrolytes, providing an environmentally benign route to complex nanoparticle libraries.^19^ In the present study, we employ this electrodeposition strategy to synthesize compositionally complex multimetallic nanoparticles spanning the Au–Ir–Pt–Pd–Rh–Ru composition space as a discovery platform for the electrochemical oxidation of d-glucose (glucose oxidation reaction, GOR). The GOR has broad relevance for applications ranging from glucose sensing for diabetes diagnostics to the electrocatalytic production of gluconic and glucaric acids as value-added chemicals.^20–24^ For all applications, the catalyst must operate at low overpotentials and exhibit high resistance to electrochemical poisoning, *i.e.*, the catalyst must maintain a high number of available active sites despite the presence of adsorbed intermediates and side products.^25–30^ The Au–Ir–Pt–Pd–Rh–Ru composition space was selected for this study based on previous reports of high GOR activity for Au, Pt and Pd, as well as the oxophilic nature of Ru and Ir and the high stability of Rh.^31–36^

In this work, we establish a unified GOR measurement protocol using a rotating disk electrode (RDE) setup that enables the determination of both “catalytic activity” and “poisoning resistance” for each screened nanoparticle composition. “Catalytic activity” is quantified by the chronoamperometric oxidation current density measured at a defined potential and normalized to the nanoparticle-covered projected area. “Poisoning resistance” is quantified as the ratio of the current measured after a defined time interval to the initial current. The inclusion of poisoning resistance as an objective is motivated by previous studies identifying surface poisoning as a major limitation in the electrochemical oxidation of small organic molecules, including formic acid oxidation, methanol oxidation, glucose oxidation, and glycerol oxidation.^25–30,37–39^

The two performance metrics defined in this study enable multi-objective analysis and are modeled separately using Gaussian process regression (GPR) to map the full Au–Ir–Pt–Pd–Rh–Ru composition space.^5^ By testing more than 80 distinct nanoparticle compositions,^40^ we identify compositional regions associated with high activity and high poisoning resistance, respectively. Pareto analysis subsequently identifies optimal trade-offs between activity and poisoning resistance, thereby guiding the rational design of multimetallic nanoparticle catalysts for the GOR.

## Results and discussion

Fig. 1 summarizes the closed-loop workflow used for the medium-throughput optimization of Au–Ir–Pt–Pd–Rh–Ru nanoparticle catalysts for the GOR. The workflow comprises three experimental steps (A)–(C), also shown in the SI in Fig. S1, and one machine-learning step (D), which together form a closed optimization loop (E). The workflow begins with nanoparticle synthesis *via* electrodeposition, in which nanoparticles of varying compositions are deposited onto glassy carbon (GC) substrates using different noble-metal precursor ratios in aqueous media while maintaining a constant total metal–cation concentration of 500 µM.^19^ Fig. S2 presents the pulsed electrodeposition protocol applied to all synthesized compositions. For each composition, three samples (*i.e.*, three individual GC electrodes) were prepared using the same metal precursor ratio in the electrolyte (see Methods).

**Fig. 1:**
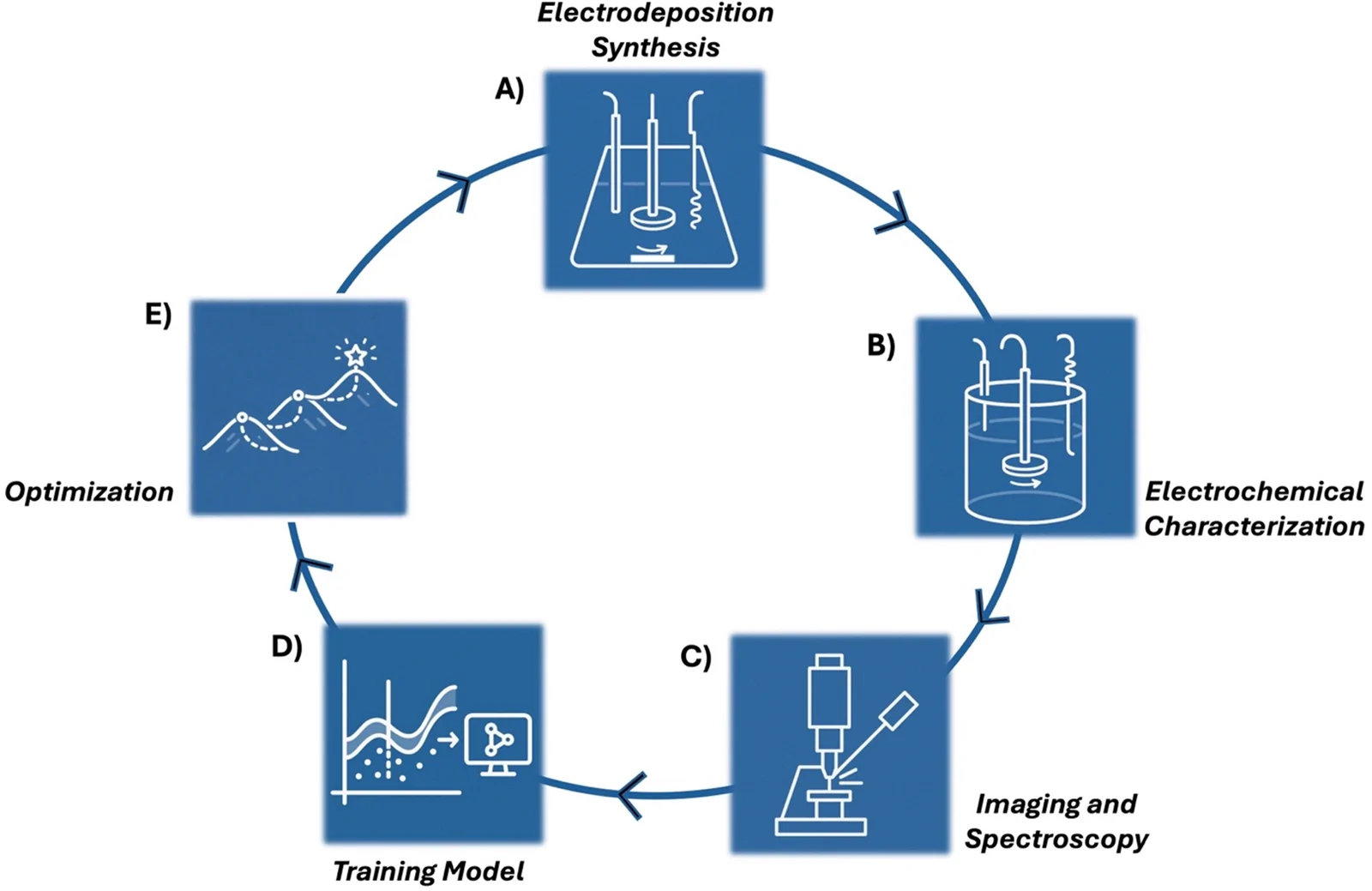
Workflow of the catalyst optimization loop. (A) Pulsed electrodeposition is used to synthesize Au–Ir–Pt–Pd–Rh–Ru catalysts with different compositions on glassy carbon electrodes. (B) Electrochemical measurements are performed using a fixed potential protocol to determine the glucose oxidation rate and its time dependence. (C) Scanning electron microscopy coupled with energy-dispersive X-ray spectroscopy (SEM-EDX) is used to determine the nanoparticle coverage and average catalyst composition. (D) The EDX-derived average nanoparticle composition as well as the nanoparticle-coverage-normalized current density and its time dependence, respectively, are used to train Gaussian process regression (GPR) surrogate models for “activity” and “poisoning resistance”. (E) The trained models identify new compositions for experimental evaluation, completing the iterative optimization loop.

Following synthesis, the nanoparticles were evaluated electrochemically toward the GOR using the potential protocol shown in Fig. 2 and Fig. S3. The same samples were subsequently characterized by scanning electron microscopy (SEM) and energy-dispersive X-ray spectroscopy (EDX) to determine nanoparticle coverage (see Fig. S4 for the thresholding) and composition. Representative EDX spectra are shown in Fig. S5. Details on determining the average nanoparticle composition are provided in Fig. S6 and S7 as well as in our previous work;^19^ Fig. S8 provides an overview of the compositional variability of the catalyst samples. Once data from the initial six corner compositions had been collected, GPR surrogate models were trained to guide subsequent experiments (further information is provided in Fig. S8). The experimental data were collected sequentially, and the GPR models were updated after each experimental iteration to guide the selection of the next composition. Because inactive compositions provide little information regarding poisoning resistance, the first 40 synthesized compositions (with three repeats each to capture noise) focused on maximizing activity before additional 40 compositions were sampled around the emerging activity optima.

**Fig. 2:**
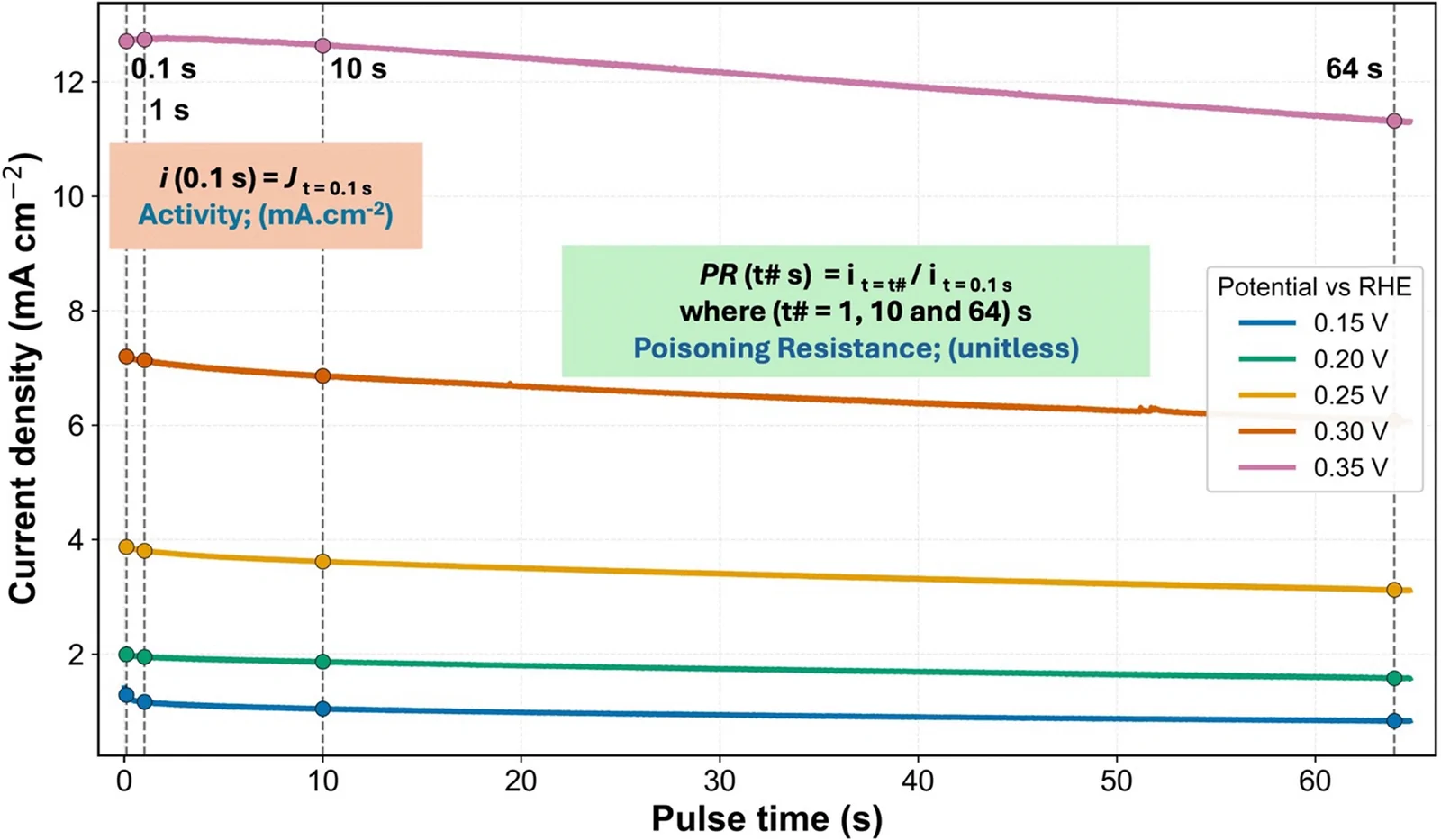
Electrochemical protocol and definition of the optimization objectives. Representative electrochemical response of an Au_0.61_Pt_0.34_Rh_0.05_ catalyst illustrating the activity and poisoning resistance metrics. The measurements were performed in a rotating disk electrode (RDE) configuration at 2500 rpm under continuous Ar purging in 1 M NaClO_4_ (pH 12) containing 0.1 M d-glucose. The same potential pulse protocol was applied to more than 80 catalyst compositions synthesized by pulsed electrodeposition on 5 mm glassy carbon working electrodes. The SEM images were used to determine the nanoparticle coverage (projected geometric area) for the normalization of the current densities (activity defined as nanoparticle-coverage-normalized current density), while SEM-EDX provided the average catalyst compositions used as model inputs.

Fig. 2 illustrates the objectives defined for the optimization of the GOR catalyst composition. The first objective is the coverage-normalized current density measured after 0.1 s, *i*(0.1 s), the “activity objective”, while the second objective is the poisoning resistance. Poisoning is a well-known limitation during the electrooxidation of organic molecules because reaction intermediates gradually block active surface sites.^25,41–43^ Although poisoning is expected to depend on the reaction rate, no single empirical kinetic expression adequately described the experimental data (also see Fig. S9).^26–30^ This observation alone does not establish that the underlying reaction mechanism changes with catalyst composition. We therefore quantify the transient response using the relative current-retention descriptor PR(*t*) = *i*(*t*)/*i*(0.1 s), without assigning it to a unique kinetic or poisoning mechanism. Gaussian process regression is used as a non-parametric surrogate model to capture composition–potential–response relationships and does not infer elementary kinetic parameters. Accordingly, composition-dependent variations in PR are interpreted as empirical composition–property correlations, while mechanistic interpretations concerning adsorbate formation and removal are presented only as literature-supported hypotheses.

To prevent inactive samples with little current decay from being favored by the poisoning-resistance metric, penalties were introduced for low-activity compositions (see Methods). The choice of several time scales for the poisoning resistance objective is motivated by the different time-dependent current responses observed during the GOR. As shown in Fig. S9, measurements at lower potentials exhibit a rapid initial current decay. In contrast, at higher potential, such as 0.35 V_RHE_, no clear overall decay pattern was observed.

We first discuss the results obtained at an electrode potential of 0.20 V_RHE_, see Fig. 3. At this potential, the GOR exhibited sufficiently high current densities and the results were relatively insensitive to the synthesis duration, see Figure S10. In addition, the GOR currents obtained at 0.20 V_RHE_ exhibit a linear dependence (0.017 mA cm^−2^ mM^−1^) on the glucose concentration over the range of 1–100 mM, which is desirable for applications such as sensing and suggests a dominant kinetic component in the recorded oxidation currents, see Fig. S11.^44^ Last but not least, 0.20 V_RHE_ was the lowest potential at which the GOR currents could be clearly distinguished from currents obtained from the oxidation of other potentially interfering organic molecules such as ascorbic acid, lactic acid, uric acid, and paracetamol, see Fig. S12, and hence high selectivity for d-glucose oxidation can be assumed.

**Fig. 3:**
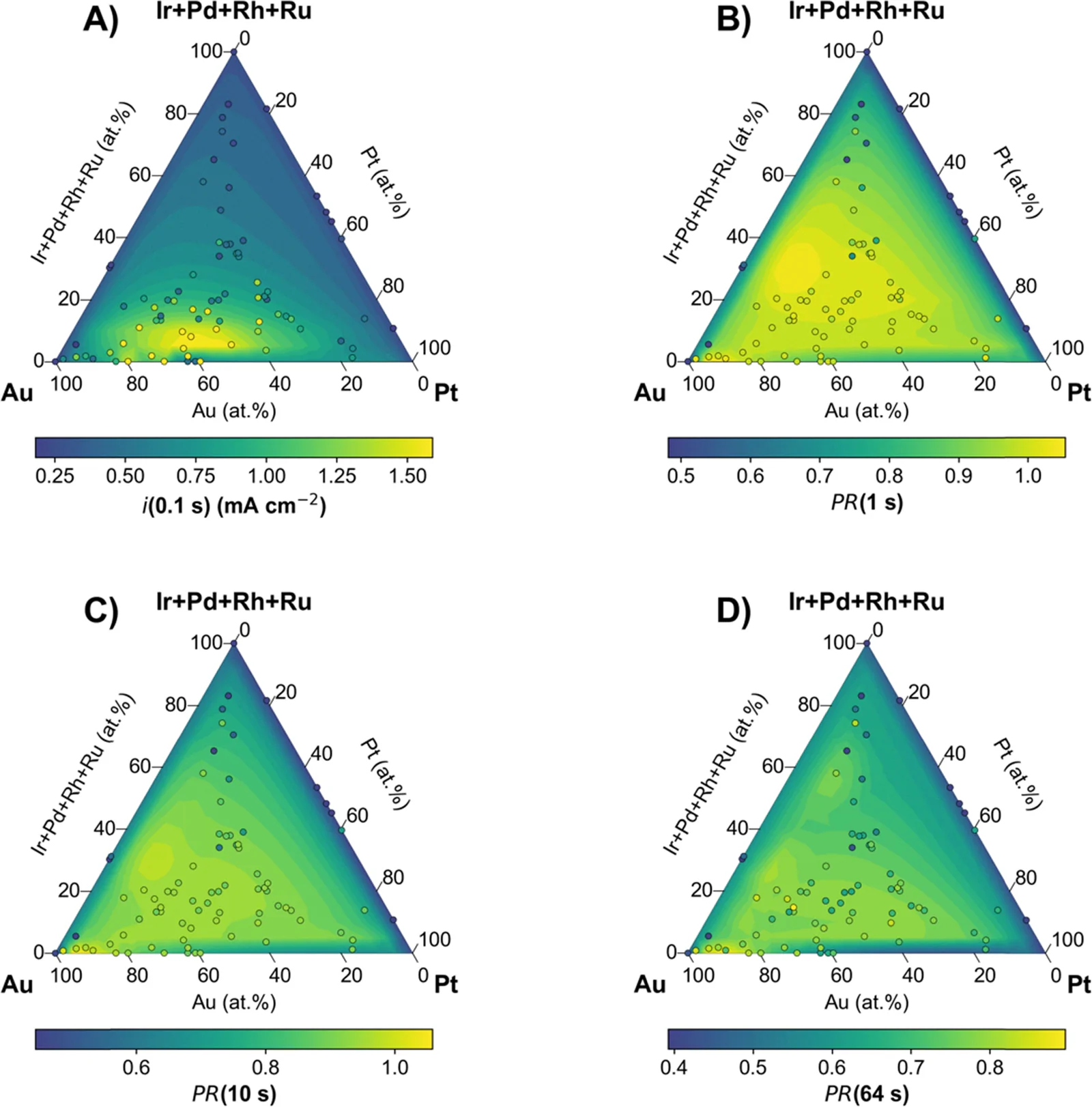
Surrogate model predictions at 0.20 V_RHE_ across the Au–Ir–Pt–Pd–Rh–Ru composition space. Simplex plots showing the predicted mean values of the Gaussian process regression models for (A) activity, *i*(0.1 s); (B) poisoning resistance after 1 s, PR(1 s) = *i*(1 s)/*i*(0.1 s); (C) poisoning resistance after 10 s, PR(10 s) = *i*(10 s)/*i*(0.1 s); and (D) poisoning resistance after 64 s, PR(64 s) = *i*(64 s)/*i*(0.1 s). Predictions were evaluated on a compositional grid with 5 at% resolution. Compositions with activity below the 25th percentile were assigned low poisoning resistance values to exclude inactive catalysts from the optimization.

In Fig. 3A, the results from the obtained GPR model of the composition-activity space at 0.20 V_RHE_ are shown. The predicted activity (mean) across a 5 at% grid is visualized as a heat map in a simplex representation of Au, Pt and Ir–Pd–Rh–Ru. The experimental data points are projected onto the simplex and indicated by circular markers. The GPR model predicts high GOR activity localized along the Au–Pt-rich side of the simplex, with approximately 10–15 at% contributions from Ir, Pd, Rh, and Ru. A comparison of the different GPR activity models and their uncertainties for the other electrode potentials investigated (0.15, 0.20, 0.25, 0.30 and 0.35 V_RHE_) is shown in Fig. S13 and S14, respectively.

The “activity” and “poisoning-resistance” predictions differ substantially at 0.20 V_RHE_, indicating that highly active compositions are not necessarily the most poisoning resistant. Furthermore, different poisoning resistance maps evolve with time. Short-term current retention (PR(1 s)) is favored across a relatively broad range of Au-rich compositions containing moderate Pt and minor amounts of Ir, Pd, Rh, or Ru.^45,46^ As the observation time increases to 10 and 64 s, the favorable region progressively contracts toward the Au–Pt edge, indicating that sustained resistance to poisoning requires a more specific compositional balance.

These simplex representations should be interpreted cautiously because they substantially reduce the dimensionality of the composition space. Furthermore, the obtained results show a clear dependence on electrode potential. Fig. 4 therefore provides a condensed summary of the overall results obtained, *i.e.*, the GPR models of all measurements at the five different electrode potentials, displaying the top 10 predicted compositions per potential in box plots.

**Fig. 4:**
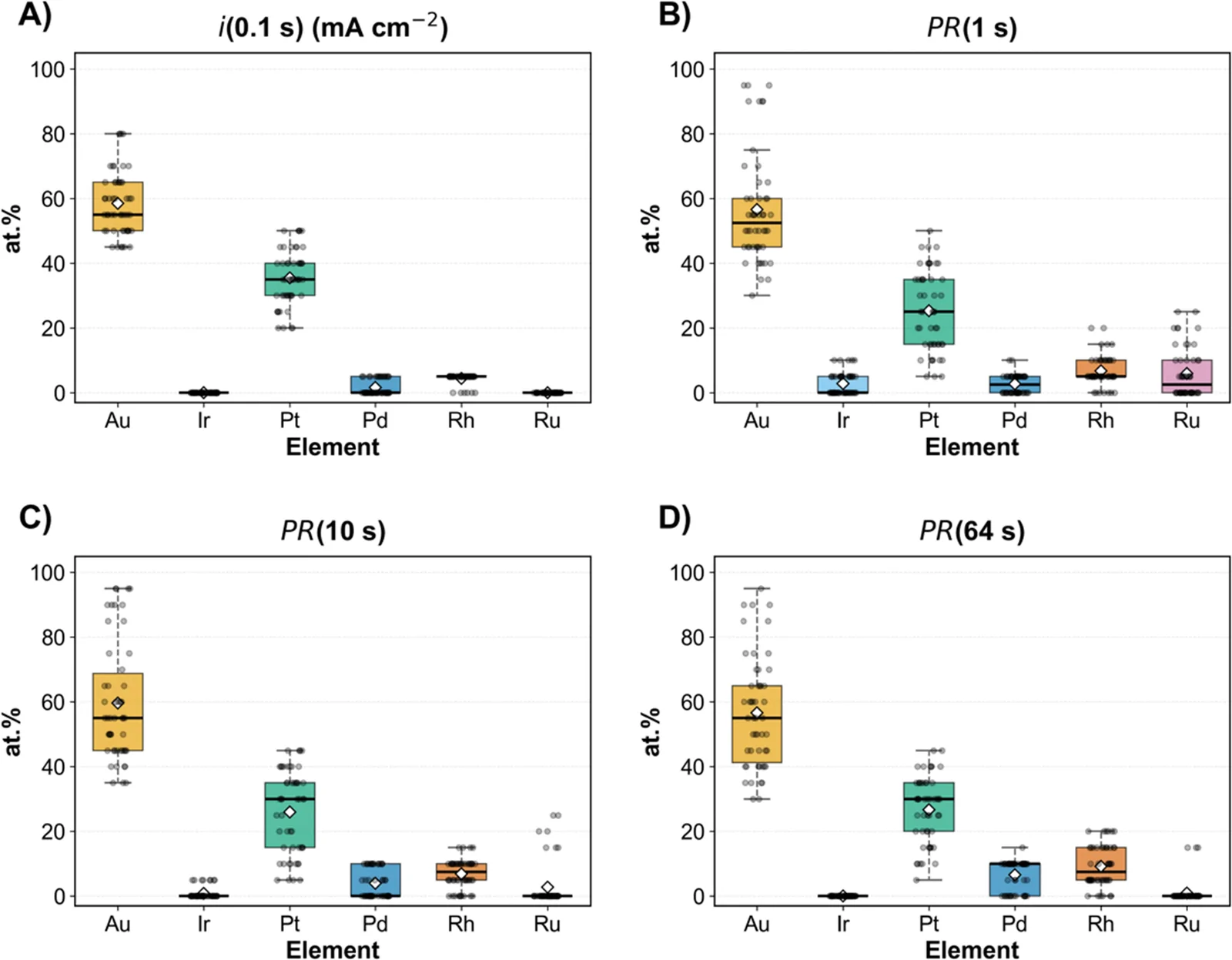
Distribution of the highest-performing catalyst compositions across different electrode potentials. Box plots (boxes, interquartile range; center line, median; whiskers, minimum to maximum; diamonds, mean) summarize the best 50 catalyst compositions (grey circles; top 10 at each investigated potential) for (A) activity, *i*(0.1 s); (B) PR(1 s); (C) PR(10 s); and (D) PR(64 s). Candidate compositions were identified using Gaussian process regression models evaluated on a 5 at% compositional grid.

The highest predicted GOR activities are consistently associated with Au-rich compositions containing 45–80 at% Au and 20–50 at% Pt. Rh and Pd appear only as minor alloying elements, whereas Ir and Ru contribute little to catalytic activity. Cross-validation demonstrates good predictive performance for potentials ≥0.20 V_RHE_ (Fig. S15), while SHapley Additive exPlanations (SHAP) analysis (Fig. S16) identifies Au and Pt as the dominant contributors to activity. Rh and Pd provide smaller positive contributions, whereas Ir and Ru are largely neutral or detrimental. Together, these results consistently identify Au–Pt-rich compositions as the primary “activity” optimum. These composition ranges are in agreement with previously reported catalysts for the GOR, whereas no systematic studies of the poisoning resistance could be found.^32,41,47,48^

By comparison, the summary of the top candidates for “poisoning resistance” at the five probed electrode potentials indicates dominant roles of Au and Pt as well. For PR(1 s), the top candidates are Au-rich with substantial amounts of Pt, but they also contain noticeable amounts of Ir, Pd, Rh and Ru in a subset of compositions. In contrast, PR(10 s) and PR(64 s) exhibit secondary contributions of Pd and Rh to the poisoning resistance, while Ir and Ru contributions are absent. Fig. S17–S22 show composition – poisoning resistance correlations and their uncertainties are shown in their simplex representation. Cross-validation (Fig. S23–S25) and SHAP analysis (Fig. S26–S28) support these overall trends. Au gives the most consistent positive SHAP contribution across PR(1 s), PR(10 s), and PR(64 s), while Pt also contributes positively, supporting the regions near the Au–Pt edge observed in the ternary maps and boxplots. Pd and Rh show minor but clear composition-dependent positive SHAP contributions. This is consistent with their role as secondary components (5–20 at%) in the top-candidate distributions, particularly for PR(64 s). Finally, Fig. S29 provides an alternative representation of the GPR models predicting the potential-dependence of the top compositions for activity and poisoning resistance.

### Pareto analysis of the multi-objective study

When multiple objectives are considered, there is typically no single best solution; instead, a set of Pareto-optimal solutions represents the best trade-offs among the objectives. From the differences in the landscapes of the activity and poisoning resistance correlations in simplex representation, it can be expected that this is also the case for the GOR. Hence, we performed a Pareto analysis^49^ to determine the trade-offs between activity and poisoning resistance at the different applied potentials, see Fig. 5.^49^ The Pareto-optimal solutions are indicated by thick markers and represent the best compromises between activity and poisoning resistance. The individual compositions corresponding to the Pareto optimal solutions are listed in Table S1.

**Fig. 5:**
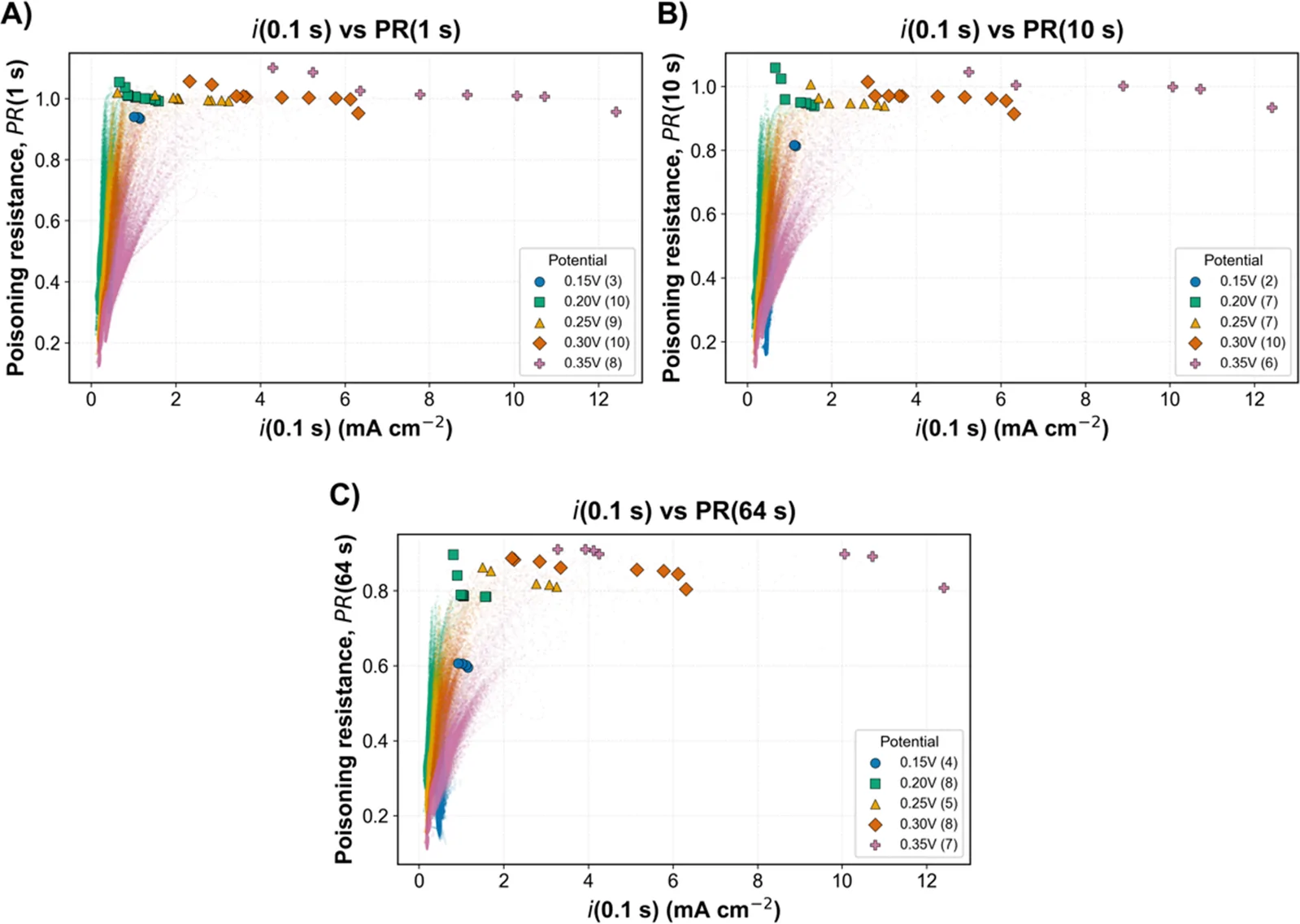
Pareto-optimal catalysts for glucose oxidation reaction. Predicted catalyst performance in the activity–poisoning resistance objective space. (A) *i*(0.1 s)-PR(1 s); (B) *i*(0.1 s)-PR(10 s); and (C) *i*(0.1 s)-PR(64 s). The thick markers represent the Pareto-optima at each electrode potential obtained by a bi-objective analysis of the Gaussian process regression models using a 5 at% compositional grid, while the dots indicate the non-optimal compositions.

The Pareto-optimal compositions depend on electrode potential and the time at which current retention is evaluated, consistent with the observation that “poisoning resistance” is challenging to describe by a single empirical relation (see Fig. S9). Increasing the applied potential shifts the Pareto front progressively to the right, reflecting the expected increase in initial catalytic current with electrode potential. At 0.35 V_RHE_, the Pareto front extends to activity values roughly one order of magnitude higher than those at 0.15 V_RHE_. Furthermore, the Pareto front contracts markedly from PR(1 s) to PR(64 s), indicating that high poisoning resistance is challenging to maintain over longer timescales. Interestingly, the maximum values for the poisoning resistance of the Pareto optimal solutions increase primarily as the electrode potential is raised from 0.15 V_RHE_ to higher electrode potentials. In addition, the relatively high glucose concentration of 100 mM is expected to lead to a relatively fast accumulation of chemisorbed species at the surface.^45^ Hence, the highest values of the poisoning resistance metric do not substantially increase as the electrode potential is raised from 0.20 to 0.35 V_RHE_. Increasing the applied electrode potential beyond 0.20 V_RHE_ primarily amplifies the initial catalytic turnover rather than substantially mitigating surface poisoning.

The contraction of the Pareto set from PR(1 s) to PR(64 s) indicates that fewer compositions simultaneously maintain high initial activity and current retention as the pulse duration increases. This trend is consistent with the established understanding of alkaline GOR, where the transient current can reflect not only the initial glucose activation but also the evolving balance between formation and removal of glucose-derived oxygenated adsorbates.^32,50–52^ Similar time-dependent current losses have been associated with the accumulation of adsorbed intermediates during methanol, formic-acid, ethanol, and glycerol electrooxidation on noble-metal catalysts.^26–30^ The Au–Pt-rich region identified at longer times may therefore reflect a favorable balance between glucose activation and the removal or reduced persistence of blocking intermediates. However, because adsorbates were not directly measured in the present study, this interpretation is presented as a literature-supported hypothesis rather than evidence for a specific poisoning intermediate or pathway.

Fig. 6 summarizes the results using box plots. The Pareto-optimal compositions for short-term current retention (PR(1 s)) are dominated by Au and Pt, with median values of approximately 65 at% Au and 27.5 at% Pt. Rh is present at approximately 5 at%, while Pd appears only in selected compositions and Ir/Ru are nearly absent with two outliers. For *i*(0.1 s) *versus* PR(10 s), the Pareto set becomes even more Au-rich, with a median of 70 at% Au and 25 at% Pt, while Ru and Ir are completely absent from the Pareto compositions. For long-time current retention, *i*(0.1 s) *versus* PR(64 s), the Pareto compositions remain Au–Pt-rich, with medians of 60 at% Au and 30 at% Pt. In addition, stronger secondary contributions from Rh (up to 20 at%) and Pd (up to 10 at%) are seen. This suggests that compositions containing Pd and Rh can be particularly useful for maintaining current retention over longer times. As a complementary analysis, Section S7.5 of the SI reports the same workflow using the integrated oxidation charge (*Q_*norm) as an alternative activity descriptor (Fig. S34–S41 and Table S3), which yields qualitatively similar activity trends and Pareto-optimal compositions.

**Fig. 6:**
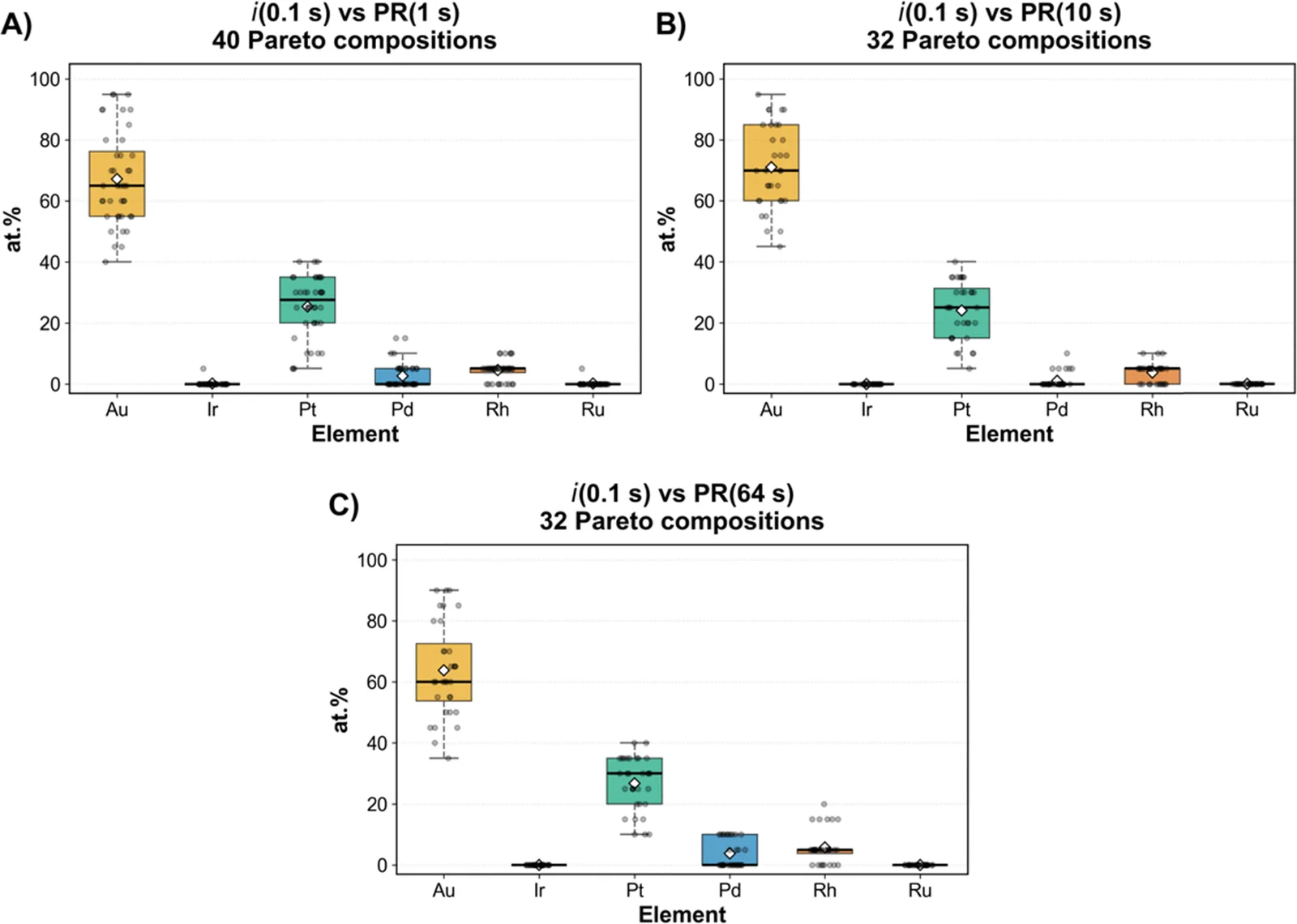
Compositional distribution of Pareto-optimal catalysts. Box plots (boxes, interquartile range; center line, median; whiskers, minimum to maximum; diamonds, mean) summarize the elemental compositions of the Pareto-optimal catalysts identified in the activity-poisoning resistance objective space. (A) *i*(0.1 s)-PR(1 s); (B) *i*(0.1 s)-PR(10 s); and (C) *i*(0.1 s)-PR(64 s).

The enrichment of the “long-term” Pareto set near the Au–Pt edge is mechanistically consistent with recent *operando* and theoretical work on Au_3_Pt nanowires.^32^ In that study, Pt–Au coordination was proposed to facilitate glucose oxidation while promoting the desorption of strongly adsorbed gluconolactone-related intermediates from Pt-containing sites. In the present experiments, Au–Pt-rich particle-population-average compositions may therefore provide a favorable balance between glucose activation and reduced persistence of blocking oxygenated intermediates.

The preference for Au–Pt-rich compositions over more compositionally complex regions may additionally reflect a balance between beneficial multielement interactions and dilution of favorable active-site environments as elemental complexity increases.^14^ However, because adsorbates and local active-site configurations were not directly measured in the present study, these interpretations are presented as literature-supported hypotheses rather than evidence for a specific poisoning intermediate or pathway.

Moreover, our results indicate that increasing the elemental complexity of an electrocatalyst does not necessarily lead to improved GOR performance.^14^ Within the investigated Au–Ir–Pt–Pd–Rh–Ru composition space and under the electrochemical conditions employed in this study (pH 12, 0.1 M glucose), the optimization did not favor the compositionally most complex quinary or senary nanoparticles. Although Ir and Ru were included in the screening because of their oxophilic character, neither element was strongly represented among the high-performing or Pareto-optimal compositions. In particular, the optimization did not identify substantial Ir- or Ru-rich regions within the best-performing composition space. Instead, the Pareto-optimal regions were predominantly associated with compositionally simpler, Au–Pt-rich binary and ternary systems, together with selected quaternary compositions containing Pd and Rh. The SHAP analysis further supports these element-specific composition–property correlations, although it does not provide direct insight into the underlying elementary surface reaction mechanisms.

Notably, the limited representation of Ir and Ru among the high-performing compositions under the investigated conditions does not imply that these elements are inherently unfavorable for GOR, as GOR activity and reaction pathways are known to depend strongly on electrolyte pH and mass-transport conditions.^53,54^ These findings therefore motivate extending the compositional screening of the Au–Ir–Pt–Pd–Rh–Ru system to neutral and acidic electrolytes, where the influence of oxophilic elements such as Ir and Ru on GOR performance may differ from that observed under alkaline conditions.

## Methods

### Chemicals and gases

Hydrogen tetrachloroaurate(iii) trihydrate (CAS-16961-25-4), sodium hexachloroiridate(iv) hexahydrate (CAS-19567-78-3), dihydrogen hexachloroplatinate(iv) hexahydrate (CAS-26023-84-7), ammonium hexachloropalladate(iv) (CAS-19168-23-1), chloropentaammineruthenium(iii) chloride (CAS-18532-87-1) were purchased from Sigma Aldrich and rhodium(iii) Chloride hydrate (CAS-20765-98-4) was purchased from Alfa Aesar.

All metal precursors were stored in a glovebox and whenever required for the synthesis were weighed and individually dissolved in 5 mL Milli-Q water in glass vials as stock solutions and stored in a refrigerator (outside the glovebox). Argon with a purity of 99.999% (grade 5.0) was used to purge the electrolyte.

### Electrodeposition

The electrodeposition was performed in a three-neck cell using a Nordic Electrochemistry potentiostat ECi-210. The apparent solution resistance was determined by superimposing an alternating potential of 5 mV and 5000 Hz and an online compensation scheme provided by the potentiostat was used to reduce the cell resistance.

Glassy carbon (GC) disks with a geometric area of 0.196 cm^2^ (5 mm diameter) were used as working electrode (WE). A GC rod of 5 mm diameter was used as the counter electrode (CE). The section exposed to the electrolyte had a length of 3 cm. An Ag/AgCl in 3 M KCl solution was used as the reference electrode (RE). The potential offset relative to RHE was determined experimentally at regular intervals. The glassy carbon (GC) disk electrodes were polished using a 50 nm alumina (Al_2_O_3_) slurry. Before synthesis, the cleaned surfaces were inspected *via* optical microscopy to confirm the absence of large alumina particle agglomerates.

An aqueous solution of 0.1 M sodium chloride (NaCl, initial pH 5.5) served as the supporting electrolyte, containing a total metal precursor concentration maintained at 500 µM. While the total metal concentration remained constant, the relative ratios of the metal precursors were systematically varied. Because static GC disks were used without rotation, a Teflon stir bar was employed to agitate the deposition electrolyte. Additionally, a continuous flow of Ar was maintained to remove dissolved oxygen. The final electrolyte volume in the deposition cell was 12 mL. Upon the addition of the metal precursors, the solution pH decreased from 5.5 to approximately 3–4. During this addition, the electrolyte was continuously purged with Ar gas while the RE was positioned in the cell. Following 15 minutes of purging, the CE, previously cleaned *via* sonication in Milli-Q water and dried under Ar flow, was introduced. Immediately thereafter, the precleaned WE was immersed into the electrolyte under a protective potential of >1.1 V *vs.* RHE. This specific potential prevented any premature electrodeposition onto the substrate before the start of the experiment.

Following the fresh preparation of the bulk electrolyte, an electrochemical pulsing sequence was applied for designated time intervals to deposit the desired nanoparticles. First, an insertion pulse was held at 1.0 V *vs.* Ag/AgCl for 2 minutes, followed immediately by a nucleation pulse at −0.5 V *vs.* Ag/AgCl for 2 seconds. Subsequently, a continuous pulse sequence consisting of a rest pulse (*E*_rest_ = 0.75 V *vs.* Ag/AgCl) and a deposition pulse (*E*_dep_ = −0.5 V *vs.* Ag/AgCl) – each lasting 0.01 seconds – was applied for a total duration of 90 seconds.

Three replicate samples were prepared from a single electrolyte bath. The three replicates were deposited onto three separate GC disks. For data representation, each sample condition (treated as input features) is cross-referenced with its respective average energy-dispersive X-ray spectroscopy (EDX) composition.

### Electrochemical characterization

Glucose oxidation reaction (GOR) measurements were performed in a cleaned glass cell. The electrolyte consisted of 100 mL of an aqueous solution containing 0.1 M d-glucose and 1 M NaClO_4_ adjusted to pH 12. A gold (Au) spiral wire and a saturated calomel electrode (SCE, Hg/Hg_2_Cl_2_ in saturated KCl) were utilized as the CE and RE, respectively. Prior to and during all electrochemical measurements, the electrolyte was continuously purged with argon to eliminate dissolved oxygen.

To evaluate the three replicate samples from each batch, a rotating disk electrode (RDE) configuration was employed at a constant rotation speed of 2500 rpm. The testing protocol for each sample comprised three potentiodynamic scans from −0.9 to −0.5 V *vs.* SCE at a scan rate of 50 mV s^−1^, followed by five consecutive potentiostatic steps held for 65 seconds each at −0.8, −0.75, −0.7, −0.65, and −0.6 V *vs.* SCE. These applied potentials correspond to 0.15, 0.20, 0.25, 0.30, and 0.35 V *vs.* RHE, based on repeated experimental calibration of the SCE. The same automation macro was executed for all replicates using the same freshly prepared electrolyte volume. After characterization of each three-replicate set, the final solution pH was verified to remain at 12, the cell was thoroughly rinsed with Milli-Q water, and fresh electrolyte was introduced for the subsequent sample group.

### Imaging and spectroscopy

Scanning electron microscopy (SEM) and EDX were performed using a Zeiss GeminiSEM 450 equipped with a Schottky field-emission gun (a single-crystal tungsten tip with a zirconia coating). The overall imaging and spectroscopy workflow implemented in this study is illustrated in Fig. S4. For surface imaging, the accelerating voltage, probe current, and working distance were set to 5 kV, 100 pA, and 5 mm, respectively. Areal scans with a resolution of 1024 × 768 pixels were acquired using an Inlens detector at a SmartSEM scan speed setting of 10 across various magnifications. To ensure consistent spatial sampling across all samples, specific regions of interest (ROIs) were targeted on each GC disk. Micrographs were systematically collected from the center of the disk as well as from the top, bottom, right, and left peripheral boundaries. A total of 10–15 images were acquired per sample to quantify the nanoparticle surface coverage, defined here as the two-dimensional projection of the nanoparticles onto the GC plane.

The nanoparticle coverage was quantified from each raw image (TIF format) using Otsu's thresholding method.^57^ This non-parametric approach converts a greyscale image to a binary image by automatically determining an optimal pixel-intensity threshold. In this study, the fractional particle coverage was calculated as the ratio of white pixels (representing the particles) to the total pixel count of the image. The mean coverage computed across all images for a given sample was defined as the average particle coverage. This average coverage value was subsequently utilized to normalize the electrochemical current response of its respective sample. Accordingly, the activity descriptor was defined as nanoparticle-coverage-normalized current density. Normalization by the ECSA derived from double-layer capacitance (*C*_dl_) was not applied because neither the non-faradaic potential window nor the specific double-layer capacitance can be assumed to be composition-independent across the chemically diverse Au–Ir–Pt–Pd–Rh–Ru nanoparticle library.^58^ Furthermore, the measured *C*_dl_ would include contributions from both the nanoparticles and the exposed glassy-carbon substrate, with their relative contributions depending on nanoparticle coverage. Therefore, nanoparticle-coverage-normalized current density was adopted as a consistent electrode-level activity descriptor for high-throughput comparisons.

Following the coverage determination, the nanoparticles were mechanically transferred from the GC disks onto holey carbon-coated copper TEM grids which were assembled in a grid holder made from Cu.^19^

For these measurements, the accelerating voltage, probe current, and working distance were set to 20 kV, 100 pA, and 8.5 mm, respectively. Backscattered electron (BSE) and scanning transmission electron microscopy (STEM) detectors, including a high-angle annular dark-field (HAADF) detector, were used to locate nanoparticle agglomerates. Characteristic X-ray spectra were acquired using an Oxford Instruments AZtecLive system equipped with an Ultim Max 150 mm^2^ large-area EDX detector. For each sample, five to six distinct nanoparticle agglomerate regions were selected for spectral acquisition, yielding a total of 30–50 spectra per sample. Acquisition parameters included a live time of 60 s, a dwell time of 20 µs, and a process time setting of 6.

Quantitative elemental analysis was performed using AZtec software (version 6.1, Oxford Instruments). Standardless matrix corrections were applied automatically to account for X-ray absorption and secondary fluorescence effects.^19^ The reported average compositions were obtained directly from the AZtec software, and their actual experimental uncertainties may exceed those estimated by the software.

The compositions used as descriptors in the screening and GPR analysis represent particle-population-average compositions determined by SEM-EDX. These values were not independently validated by bulk elemental analysis, such as inductively coupled plasma mass spectrometry (ICP-MS), and should not be regarded as independently verified bulk stoichiometries. Moreover, SEM-EDX does not resolve nanoscale compositional heterogeneity within individual nanoparticles.^19^ Consequently, the composition–property relationships established in this study should be interpreted as correlations with particle-population-average compositions rather than local nanoscale or atomic-scale compositions. As demonstrated in Fig. S1, the nanoparticles average composition of one repeat was assigned to all the three repeats. This was done as all the three repeats were made from the same bulk electrolyte concentration. To make this assumption statistically relevant 50–100 spectrum across thousands of nanoparticles were taken into account to get an average composition descriptor.

Finally, the output features extracted from the GOR measurements were paired with their corresponding input features (average nanoparticle compositions) to construct a structured dataset for subsequent machine learning analyses.

### Data analysis

The electrochemical current-transient data were processed directly from the raw TDMS files and mapped against a curated metadata table containing sample names, elemental compositions, average catalyst coverages, and coverage standard deviations. For each sample, the current response and the corresponding time axis were extracted from the respective electrochemical channel. Five distinct potential steps were analyzed, corresponding to 0.15, 0.20, 0.25, 0.30, and 0.35 V *vs.* RHE. Within each potential window, the absolute time was converted to relative pulse time by subtracting the timestamp at the onset of the corresponding pulse. The measured current was converted to current density by first converting the raw current to µA and then normalizing it by the nanoparticle coverage across 0.196 cm^2^ (geometric area of a 5 mm disc) using TIF format data (Inlens images) collected from GC disks.

To facilitate interpretation of the Python scripts that are made publicly available, the specific line of code reads:*i*_norm (mA cm^−2^) = *i* (µA) × 0.001/(coverage_fraction × geometric_area)with coverage*_*fraction = Average*_*coverage/100.

To ensure statistical reliability and a robust signal-to-noise ratio, an initial data filtering threshold was applied: only samples with an average particle coverage of ≥13% were retained for downstream modeling.

Several parameters were extracted from each current–time transient. The activity was defined as the nanoparticle-coverage-normalized current density measured 0.1 s after the respective potential jump (relative pulse time), denoted as *i_t*0*p*1*_*norm in the Python scripts. To quantify catalyst poisoning, additional current densities were extracted at specific intervals of 1, 10, and 64 s to calculate the temporal current retention relative to the initial current. For each potential, the denominator was defined as: max(*i*(0.1 s), *p*25_*i*(0.1 s)), where *p*25_*i*(0.1 s) is the 25th percentile of *i*(0.1 s) across all processed samples at that potential.

The denominator was introduced to penalize low-activity samples and avoid artificially high retention values caused by division by very small initial currents. The resulting poisoning-resistance descriptors were defined as:poisoning_resistance_1 s; PR(1 s) = *i*(1 s)/max(*i*(0.1 s), p25)poisoning_resistance_10 s; PR(10 s) = *i*(10 s)/max(*i*(0.1 s), p25)poisoning_resistance_64 s; PR(64 s) = *i*(64 s)/max(*i*(0.1 s), p25)

Values were clipped only at a lower bound of zero. No upper clipping was applied; thus samples exhibiting increasing current over time, where *i*(*t*) > *i*(0.1 s), were retained as physically meaningful cases.

### Compositional heterogeneity analysis using CLR and Aitchison metrics

To investigate if the synthesized complex multimetallic nanoparticle are random or non-random solid solutions,^59^ the compositional heterogeneity was evaluated at the mesoscale, see appendix in SI (Fig. S7), using log-ratio statistics to account for the closed nature of elemental composition data, wherein all constituent fractions sum to one. For the complete SEM-EDX dataset, the six-component compositions consisting of Au, Ir, Pt, Pd, Rh, and Ru were analyzed sample-wise by grouping replicate measurements according to their respective sample identifiers. To accommodate logarithmic transformation, non-positive values were replaced with a small imputation threshold (1e-3). Notably, Au–Ir–Pt–Pd–Rh–Ru system has been investigate to undergo face-centered cubic system.^19^

Within each sample, elements were retained only if their Aitchison geometric mean contribution exceeded 2 at%, ensuring that pairwise log-ratio statistics were not artificially dominated by trace components. The centered log-ratio (CLR) transformation was subsequently calculated according to clr(*x_*_*i*_) = ln(*x_*_*i*_) - mean[ln(*x*)]. The sample variance of these CLR values was defined as the element-specific CLR variance. This metric quantifies the magnitude of fluctuations for each individual element relative to the compositional geometric center of the sample. To assess the co-dependence of elemental variations across replicate measurements, pairwise Aitchison variations *T_*_*ij*_ = Var[ln(*x_*_*i*_/*x_*_*j*_)] were calculated using sample variance (ddof = 1), to quantify how strongly each elemental ratio varies across replicate measurements. These statistical values were compiled across the entire SEM-EDX dataset and visualized as boxplots of element-specific CLR variances and pairwise Aitchison variation ratios *T_*_*ij*_.

For the multi-scale comparison between SEM-EDX and S/TEM-EDX, this same log-ratio framework was restricted to the active Au–Pt–Rh ternary composition subspace. While arithmetic means and standard deviations were used to describe the measured compositions in conventional at% units, the Aitchison geometric means, CLR variances, and pairwise *T_*_*ij*_ values were used to rigorously evaluate compositional heterogeneity in a closure-aware manner. The resulting CLR value and pairwise log-ratio distributions were visualized *via* boxplots to directly compare mesoscale (SEM) *versus* nanoscale (S/TEM) compositional variability. Combined, the global SEM-EDX analysis models population-level compositional heterogeneity across the synthesized library, while the localized S/TEM comparison resolves the variance across differing characterization length scales. Inspired by prior work,^19^ Separately non-negative matrix factorization (NMF) was subsequently performed on STEM-EDX hyperspectral datasets to resolve spectral factors and spatial loadings underlying compositional features.

### Gaussian process regression (GPR)

GPR was used to build surrogate models relating nanoparticle composition to electrochemical performance across the five applied potentials. The input descriptors were the measured average elemental fractions of the six catalyst components: Au, Ir, Pt, Pd, Rh, and Ru. Because these variables are compositional and constrained to sum to unity, the raw elemental fractions were first normalized and then transformed using a centered log-ratio (CLR) transformation with a pseudocount of 10^−3^,*x_*_CLR_ = log(*x* + pseudocount) − mean[log(*x* + pseudocount)]

Thus, the GPR models operate in a Euclidean space while preserving their constant-sum constraint.

For each objective (activity and poisoning resistance) and applied potential, the dataset was filtered to retain samples with valid target values, an average coverage of at least 13%, and positive values for objectives modelled in logarithmic space. The surrogate models were developed for the normalized initial current density, *i_t*0*p*1*_*norm, denoted as *i*(0.1 s); the normalized integrated charge, *Q_*norm_mC_cm2, denoted as Q_norm; and the poisoning-resistance descriptors poisoning_resistance_1 s, poisoning_resistance_10 s, and poisoning_resistance_64 s, denoted as PR(1 s), PR(10 s), and PR(64 s), respectively. All objectives were modelled in logarithmic space following positive-value filtering.

The GPR models were implemented using the GaussianProcessRegressor implementation in scikit-learn with target normalization (normalize_*y* = True). The covariance function corresponded to the optimized kernel identified in the kernel-selection workflow and was augmented with a WhiteKernel term to account for experimental observation noise. During prediction, the GPR posterior mean was evaluated in logarithmic space and subsequently back-transformed to the original scale using exp(*µ*). Predictions were constrained to non-negative values by clipping negative values to zero, thereby avoiding physically meaningless activity, charge, or poisoning-resistance values. The trained surrogate models were evaluated on a six-dimensional compositional simplex with a grid resolution of 5 at%, generated using an integer simplex-grid algorithm. The resulting predictions were used to construct continuous composition–property maps, identify top-performing catalyst compositions, and determine Pareto-optimal composition sets.

For visualization, the six-dimensional compositions were projected onto a reduced ternary space by retaining Au and Pt as individual axes while combining Ir, Pd, Rh, and Ru into a single pseudo-component, resulting in the coordinates (*x*_Au, *x*_Pt, *x*_Ir + Pd + Rh + Ru). Projected compositions were grouped after rounding the ternary coordinates to three decimal places. For mean objective maps, the maximum predicted value within each projected bin was retained, representing the best predicted performance along the collapsed compositional dimensions. For uncertainty maps, the median predictive standard deviation within each bin was used. For models trained in logarithmic space, the predictive uncertainty was obtained from the posterior standard deviation in log space and approximately transformed to the original scale as exp(*µ*)*σ* (approximated uncertainty). Finally, the projected ternary coordinates were converted to Cartesian coordinates for contour visualization, and the experimentally measured compositions were overlaid to indicate the sampled region of the compositional simplex.

### Cross validation

Model performance was evaluated using leave-one-group-out cross-validation (LOGO-CV in the Python script). Groups were defined by identical six-element compositions, ensuring that replicate measurements of the same catalyst composition were assigned to the same validation group and held out together during model evaluation. This grouping strategy prevented information leakage by ensuring that chemically identical compositions were never split between the training and test sets. For each LOGO-CV fold, the model was trained on all composition groups except one and subsequently used to predict the held-out group. For objectives modelled in logarithmic space, both predictions and experimental values were back-transformed to the original scale before calculating the validation metrics. Predictions from replicate measurements within each composition group were averaged to obtain one predicted value and one measured value per unique composition.

Model performance was quantified using the coefficient of determination (*R*^2^) and the mean absolute error (MAE):*R*^2^ = 1 − sum((*y*_true - *y*_pred)^2^)/sum((*y*_true − mean(*y*_true))^2^)MAE = mean(abs(*y*_true − *y*_pred))

These metrics were used both for kernel selection and for evaluating the predictive performance of the final surrogate models. Model quality was further visualized using parity plots comparing measured and LOGO-CV-predicted values, with the one-to-one line indicating perfect agreement.

### Kernel optimization

Kernel optimization was performed independently for each objective and applied potential to identify the most appropriate covariance structure for the Gaussian process surrogate models. Model selection was carried out using the filtered dataset containing samples with an average coverage ≥13%, valid target values, and positive values for objectives modelled in logarithmic space. Compositions were represented using the CLR-transformed elemental fractions described above. Five base covariance kernels (Constant, radial basis function (RBF), Matérn, RationalQuadratic, and DotProduct) were considered. In addition, all pairwise additive and multiplicative combinations, as well as three-kernel additive, multiplicative, and mixed combinations, were systematically evaluated. Each candidate kernel was assessed using LOGO-CV, where composition groups were defined by identical elemental compositions to ensure that replicate measurements were never divided between training and validation folds. During model fitting, a WhiteKernel term was added to account for observation noise, and target values were normalized prior to optimization.

For objectives modelled in logarithmic space, predictions were back-transformed before calculating *R*^2^ and MAE. Candidate kernels were ranked according to their LOGO-CV *R*^2^ values, while MAE served as a complementary measure of prediction accuracy. Although kernels containing DotProduct terms were included during optimization, the highest-performing non-DotProduct kernel was selected for the final models to avoid favoring overly linear covariance structures. The optimized kernels and fitted hyperparameters are reported in Table S2.

The optimized covariance structures indicate that activity is governed by both broad compositional trends and localized composition-dependent effects. The kernels selected for these objectives frequently combine short-length-scale RationalQuadratic components, suggesting multi-scale behavior across the compositional simplex. In contrast, the poisoning-resistance descriptors are generally characterized by smoother covariance structures, particularly for PR(1 s) and PR(10 s), consistent with broader Au/Pt-rich stability regions. The lower predictive performance observed for PR(64 s), together with larger estimated noise contributions at some potentials, suggests that sustained catalyst deactivation involves additional complexity beyond that captured by the shorter-time descriptors.

### Shapley additive explanations (SHAP) analysis

SHAP values were used to quantify the contribution of each elemental descriptor to the predictions of the trained Gaussian process surrogate models. For each objective and applied potential, the optimized GPR model was wrapped in a prediction function and interpreted using the model-agnostic shap.KernelExplainer. The same CLR-transformed compositional descriptors used during model training were employed for the SHAP analysis. For objectives modelled in logarithmic space, the GPR predictions were exponentiated before explanation so that SHAP values are expressed on the original objective scale. The Kernel SHAP background distribution consisted of the CLR-transformed training compositions used to fit each surrogate model. SHAP values were calculated for all available training compositions, thereby describing how each elemental descriptor shifts the predicted response relative to the background distribution within the experimentally sampled compositional manifold.

For visualization, features were ranked according to the mean absolute SHAP value averaged across all applied potentials, providing an overall measure of feature importance for each objective. Individual points were colored according to the corresponding elemental concentration (at%) using a Viridis color scale with power normalization to improve contrast at low and intermediate concentrations. Although the colour scale reflects the original elemental fractions, the SHAP values themselves correspond to contributions in CLR-transformed feature space.

Positive SHAP values indicate that a descriptor increases the predicted response relative to the model background, whereas negative values indicate a decrease. Consequently, the distribution of SHAP values captures both the magnitude and composition dependence of each elemental contribution. Because the elemental descriptors are compositional and constrained by the constant-sum relationship, the SHAP values should be interpreted as conditional contributions within the observed compositional manifold rather than as independent causal effects of varying individual elemental fractions.

### Multi-objective analysis

A bi-objective optimization analysis was performed to identify compositional trade-offs between electrocatalytic activity and poisoning resistance across the six-component Au–Ir–Pt–Pd–Rh–Ru composition space.

For each applied potential (0.15, 0.20, 0.25, 0.30, and 0.35 V_RHE_), independent Gaussian process surrogate models were trained for the activity descriptor *i*(0.1 s) as well as for the poisoning-resistance metrics PR(1 s), PR(10 s), and PR(64 s). The optimized covariance kernels identified during kernel selection were subsequently refitted using the complete training dataset.

The surrogate models were evaluated on a six-dimensional compositional simplex with a resolution of 5 at%, and predictions were used to construct Pareto fronts for each pair of objectives. A composition was classified as Pareto-optimal when no other composition simultaneously achieved equal or higher predicted activity and poisoning resistance, with at least one objective being strictly improved.

Pareto-optimal compositions were visualized together with the complete prediction set in objective space. The corresponding compositional trends were further summarized using elemental box plots, potential-dependent evolution plots, and tabulated Pareto-optimal compositions.

### Bayesian optimization

Bayesian optimization was performed in a sequential closed-loop framework.^60^ The optimization was initialized using six experimentally characterized compositions located near the vertices of the six-dimensional compositional design space (Au_0.94_Ir_0.0_Pt_0.03_Pd_0.01_Rh_0.02_Ru_0.0_, Au_0.23_Ir_0.52_Pt_0.12_Pd_0.04_Rh_0.09_Ru_0.01_, Au_0.06_Ir_0.02_Pt_0.80_Pd_0.03_Rh_0.04_Ru_0.05_, Au_0.16_Ir_0.03_Pt_0.011_Pd_0.61_Rh_0.06_Ru_0.03_, Au_0.13_Ir_0.04_Pt_0.07_Pd_0.02_Rh_0.67_Ru_0.05_, and Au_0.18_Ir_0.01_Pt_0.12_Pd_0.04_Rh_0.09_Ru_0.57_). These initial compositions provided the training data for the first Gaussian process surrogate model.

Following each experimental iteration comprising catalyst synthesis, GOR measurements, imaging, and spectroscopy, three newly characterized compositions were incorporated into the training dataset, and the Gaussian process model was retrained using all available CLR-transformed compositions. Candidate compositions were generated by Dirichlet sampling within the admissible compositional space and ranked according to the selected acquisition function. The next experiment was chosen either by maximizing the Expected Improvement (EI) acquisition function to exploit regions of high predicted activity or by maximizing the predictive standard deviation to promote exploration of uncertain regions. This iterative update–retrain–select procedure was repeated for a total of 40 optimization cycles, progressively refining the surrogate model while balancing exploration of unexplored compositions with exploitation of promising catalyst candidates.

## Conclusions

The experimental dataset was used to train GPR models to predict both catalytic activity, defined as nanoparticle-coverage-normalized current density, and poisoning resistance for the GOR across the six-element composition space. A central challenge in GOR electrocatalysis is catalyst poisoning by adsorbed intermediates and reaction products. Given that the measured current transients depend on both catalyst composition and applied electrode potential, poisoning resistance was assessed using experimentally determined current retention rather than mechanistic model fitting. To avoid favoring catalysts exhibiting low activity but seemingly high poisoning resistance, a penalty was introduced for low-activity compositions.

Multi-objective analysis based on these models identified Pareto-optimal compositions, narrowing the six-dimensional compositional search space to a more manageable set of experimentally actionable binary, ternary, and quaternary catalyst candidates.

The analysis indicates that Au and Pt are the primary elements associated with high catalytic activity, while additional alloying elements, particularly Pd and Rh, can contribute to improved current retention. These findings suggest that, in contrast to previous optimization studies of the oxygen reduction reaction (ORR), which have predominantly identified bimetallic compositions, selected multimetallic alloys offer promising opportunities to balance activity and poisoning resistance in GOR electrocatalysis.^32,33,41,47–49,55,56^ More broadly, this study demonstrates how medium-throughput experimentation, machine learning, and multi-objective optimization can be integrated to identify promising electrocatalyst compositions and guide experimental screening. The resulting framework is adaptable to other electrocatalytic reactions involving competing performance objectives.

## Conflicts of interest

There are no conflicts to declare.

## Supplementary Material

EY-OLF-D6EY00174B-s001

## Data Availability

All the raw data, along with key features extracted using the Python scripts can be found here: https://doi.org/10.5281/zenodo.18488326. See DOI: https://doi.org/10.1039/d6ey00174b.
