# Supplementary material for: Data-driven multi-objective analysis of activity–poisoning resistance trade-offs in complex multimetallic electrocatalysts for glucose oxidation
Source: EES Catal. 2026 Nov 5. Online ahead of print. doi: 10.1039/d6ey00174b (PMC13757965; doi:10.1039/d6ey00174b)
Supplement: Supplementary file 1 [file EY-OLF-D6EY00174B-s001.pdf]

Supplementary Information (SI) for EES Catalysis.
This journal is © The Royal Society of Chemistry

Data-Driven Multi-Objective Analysis of Activity–Poisoning resistance Trade-Offs in Complex Multimetallic Electrocatalysts for Glucose Oxidation

Divyansh Gautam[1], Ahmad Tirmidzi[1], Sarvada Shailesh Andhare[2], Nicolas Schlegel[3], Armin Asghari Alamdari[4], Jonathan Quinson[4,5], Gustav K. H. Wiberg[1,6], Matthias Arenz[1*]

[1] Department of Chemistry, Biochemistry and Pharmaceutical Sciences, University of Bern, Freiestrasse 3, CH-3012 Bern, Switzerland

[2] Metallurgical Engineering and Material Science, Indian Institute of Technology Bombay, Powai (400076) Mumbai, Maharashtra, India

[3] Department of Chemistry, University of Copenhagen, Universitetsparken 5, DK-2100 Copenhagen

[4] Biological and Chemical Engineering Department, Aarhus University, 40 Åbogade, 8200, Aarhus, Denmark

[5] CICA-Centro Interdisciplinar de Química e Bioloxía, Facultade de Ciencias, Universidade da Coruña, Campus de Elviña, A Coruña, Spain

[6] Department of Physics, University of Nevada, Reno, 1664 N. Virginia Street, Reno, NV 89557, USA

* Corresponding author: matthias.arenz@unibe.ch

* Corresponding Author

Email – matthias.arenz@unibe.ch

Table of Contents

1. DIFFERENT STEPS: EXPERIMENTAL AND COMPUTATIONAL 3
1.1 EXPERIMENTAL WORKFLOW 3
1.2 SYNTHESIS PROTOCOL 4
1.3 ELECTROCHEMICAL PROTOCOL 5
1.4 IMAGING AND NANOPARTICLE COVERAGE 6
1.5 ENERGY DISPERSIVE SPECTROSCOPY: GETTING NANOPARTICLES AVERAGE COMPOSITION 8
1.6 COMPOSITION CONTROL 9
1.7 COMPOSITION UNIFORMITY ACROSS SAMPLES 10
1.8 GAUSSIAN PROCESS REGRESSOR: TRAINING SURROGATE MODEL 11
2. EFFECT OF GLUCOSE CONCENTRATION ON THE MEASURED CURRENT - TIME 12
3. EFFECT OF DIFFERENT SYNTHESIS TIMES ON THE ACTIVITY OBJECTIVE 13
4. CATALYST SENSITIVITY AND ANALYTE SELECTIVITY: GLUCOSE ELECTROCHEMICAL OXIDATION 14
5. ACTIVITY AND POISONING RESISTANCE SURROGATES 16
5.1 ACTIVITY SURROGATE 17
5.2 POISONING RESISTANCE AT DIFFERENT TIME DOMAINS 21
5.3 CROSS-VALIDATION METHOD 27
5.4 SHAP VALUES 30
5.5 TOP COMPOSITIONS AT DIFFERENT POTENTIALS 33
6. PARETO OPTIMALS 34
7. APPENDIX 36
7.1 DISTRIBUTION OF ELEMENTS IN THE SAMPLES 36
7.2 HYPERSPECTRAL DATA: DISTRIBUTION OF ELEMENTS IN THE NANOPARTICLES 37
7.3 OPTIMIZED KERNEL 39
7.4 MEASURED CHARGE AS AN 'ACTIVITY' OBJECTIVE 40
7.5 MULTI-OBJECTIVE OPTIMIZATION IN Q_{NORM} VS PR(T S) 46

1. Different steps: Experimental and Computational

1.1 Experimental workflow

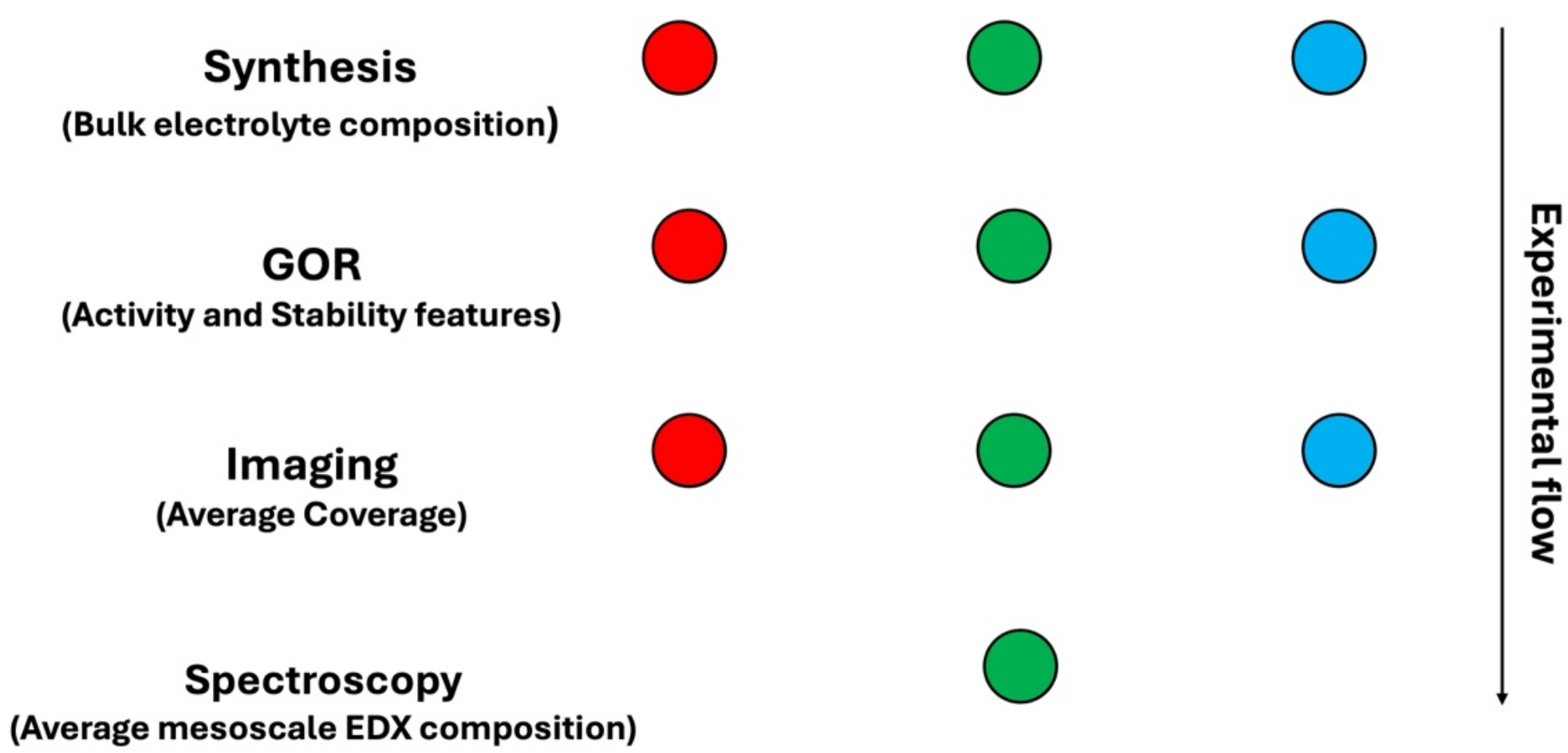

Figure S1: Experimental workflow used in this study. Three replicate samples (S0, red; S1, green; and S2, blue) were synthesized on three independent glassy carbon (GC) disks using the same bulk electrolyte composition, defined by the molar fraction ratio of the metal precursors. After electrodeposition, the three samples were individually evaluated for their glucose oxidation reaction (GOR) activity and poisoning resistance in a separate electrochemical cell. Following subsequent cleaning and drying, electron microscopy imaging was performed to determine the average nanoparticle surface coverage. The middle replicate sample was then selected for energy-dispersive X-ray spectroscopy (EDX) analysis to determine the elemental composition. Based on the EDX results,

two distinct input features were defined from the mesoscale elemental compositions obtained for the respective synthesis electrolyte compositions. The average EDX composition and the associated standard deviation were assigned to all three replicate samples synthesized from the same electrolyte composition.

1.2 Synthesis protocol

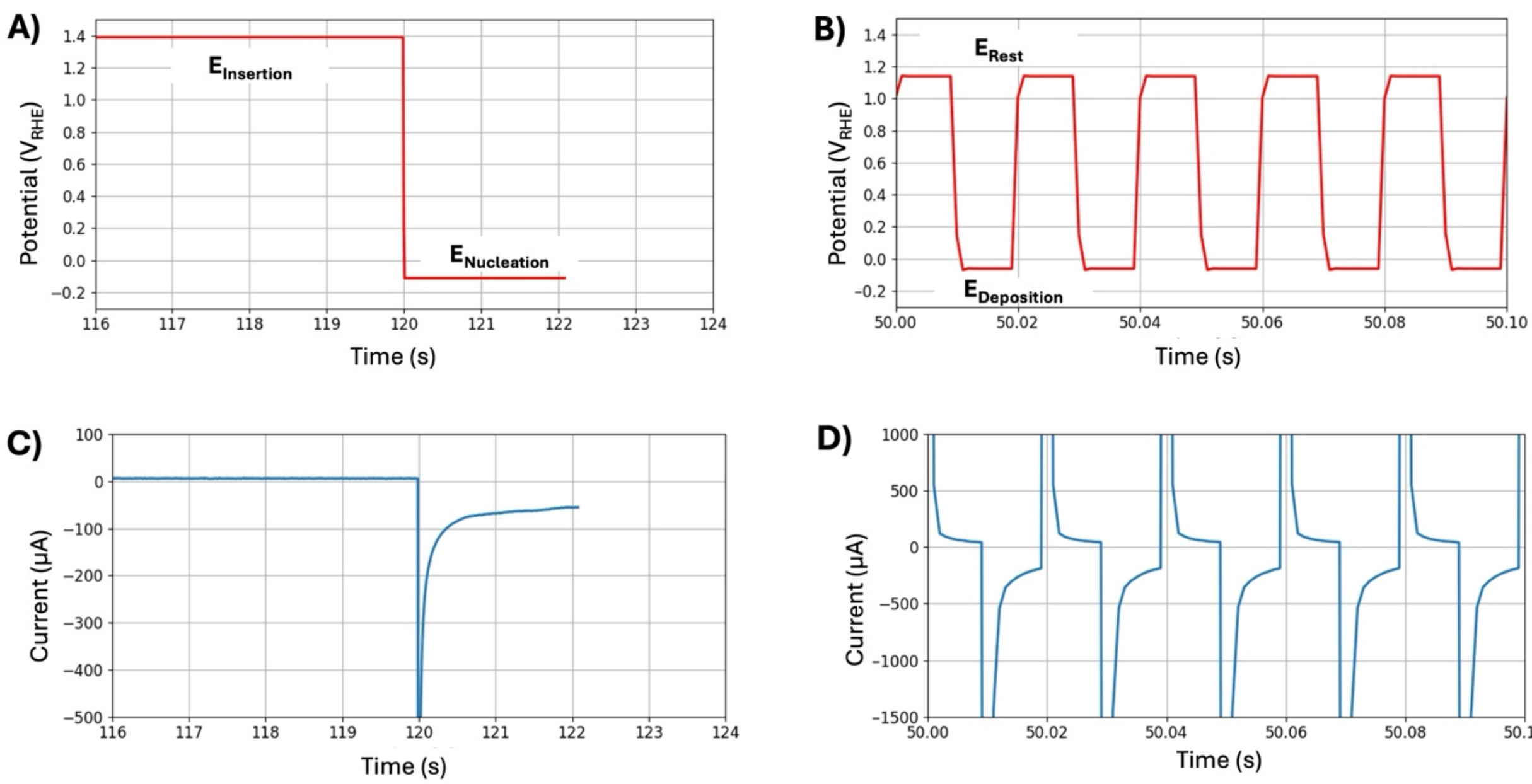

Figure S2: Electrodeposition protocol used for nanoparticle synthesis. Representative data are shown for the electrodeposition of monometallic Au nanoparticles from an electrolyte containing 500 µM Au^{3+}. A) Applied potential sequence consisting of an insertion pulse (120 s), during which the glassy carbon (GC) working electrode (WE) was immersed in the electrolyte, followed by a nucleation pulse (2 s). B) Deposition protocol to initiate particle growth employing a bi-hold waveform, alternating between E_{Rest} and $E_{Deposition}$ (0.01 s each) for 90 s. C) and D) Corresponding current–time responses recorded during the nucleation and deposition steps. The solution resistance remained between 5 and 7 Ω for all samples owing to the fixed rotating disk electrode (RDE) cell configuration.

1.3 Electrochemical protocol

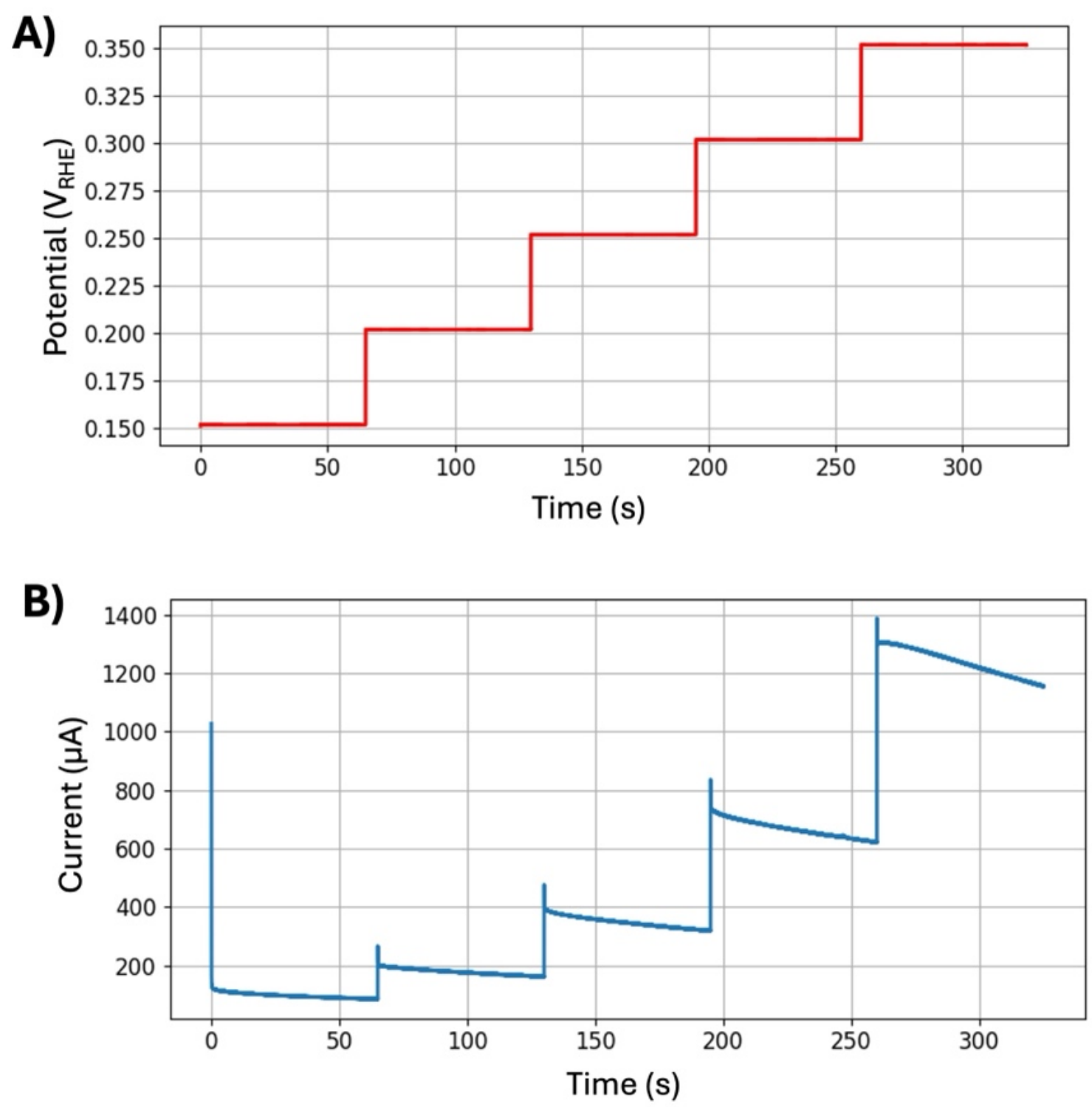

Figure S3: Electrochemical characterization protocol used to determine the activity and poisoning-resistance for the glucose electrooxidation reaction (GOR). A) Applied potential-step sequence. B) Corresponding current response. A transient non-faradaic charging current is observed after each potential step; therefore, the activities were extracted 0.1 s after each potential transition to minimize capacitive contributions.

1.4 Imaging and nanoparticle coverage

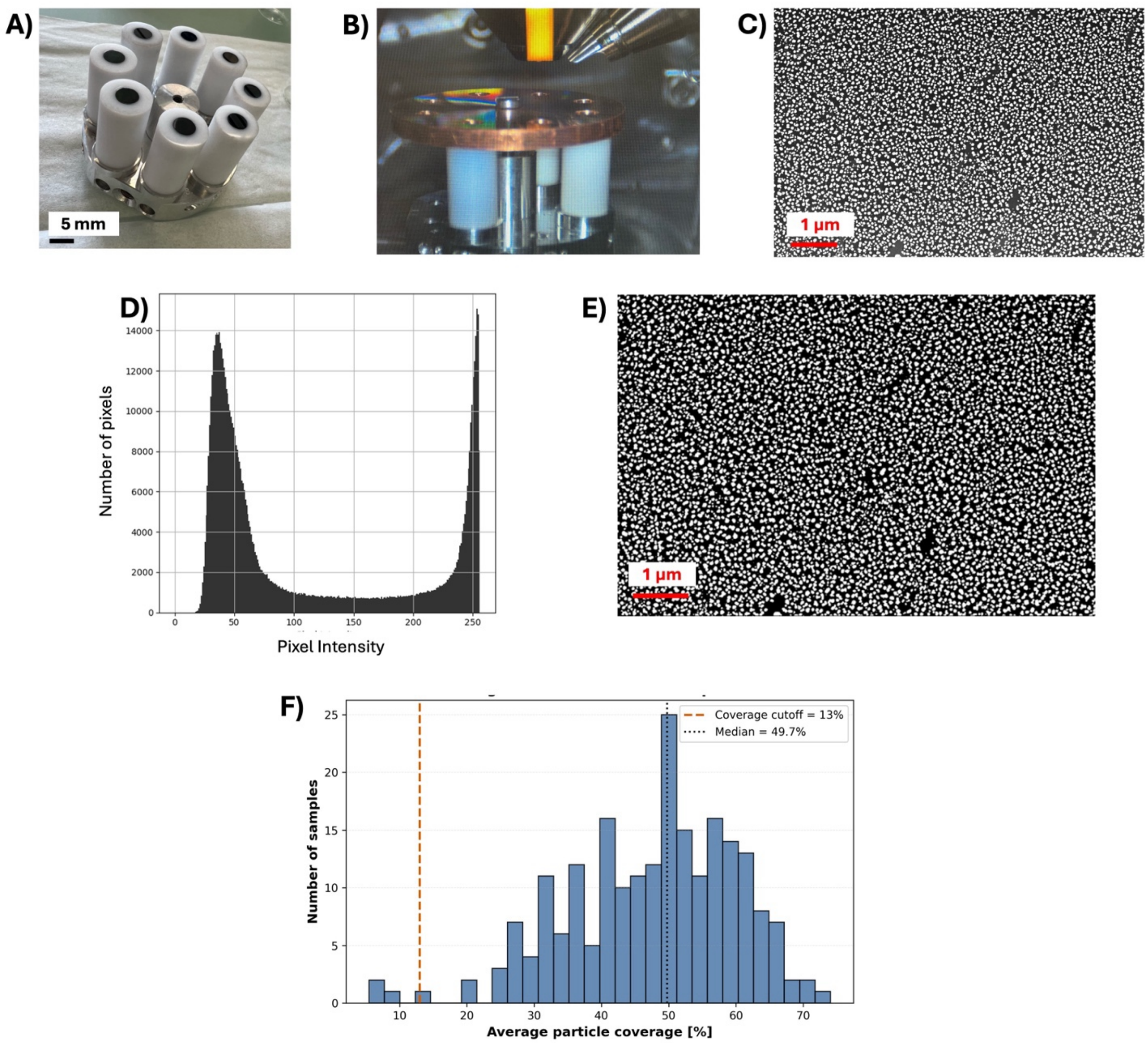

Figure S4: Scanning electron microscopy (SEM) workflow used to determine nanoparticle surface coverage on the glassy carbon (GC) electrodes. A) GC electrodes mounted on the SEM holder with electrical contact established beneath the GC disks. B) GC electrodes positioned inside the SEM chamber with a Cu plate placed on top of the GC sleeves to reduce electrostatic charging. C) Representative grayscale SEM image (1024 × 768 pixels) acquired using the InLens detector at an accelerating voltage of 5 kV, a probe current of 100 pA, and a working distance of 5 mm. D) Pixel-intensity histogram corresponding to the image shown in C). E) Binary image generated from C) using Otsu's thresholding method, which maximizes the between-class variance. F) Distribution of nanoparticle surface coverages calculated from 2,808 TIFF images acquired across all catalyst compositions investigated in this study. Samples with coverages below 13% (red dashed line) were excluded from subsequent analysis to avoid artefacts associated with normalizing electrochemical responses by extremely low nanoparticle coverages.

1.5 Energy dispersive spectroscopy: getting nanoparticles average composition

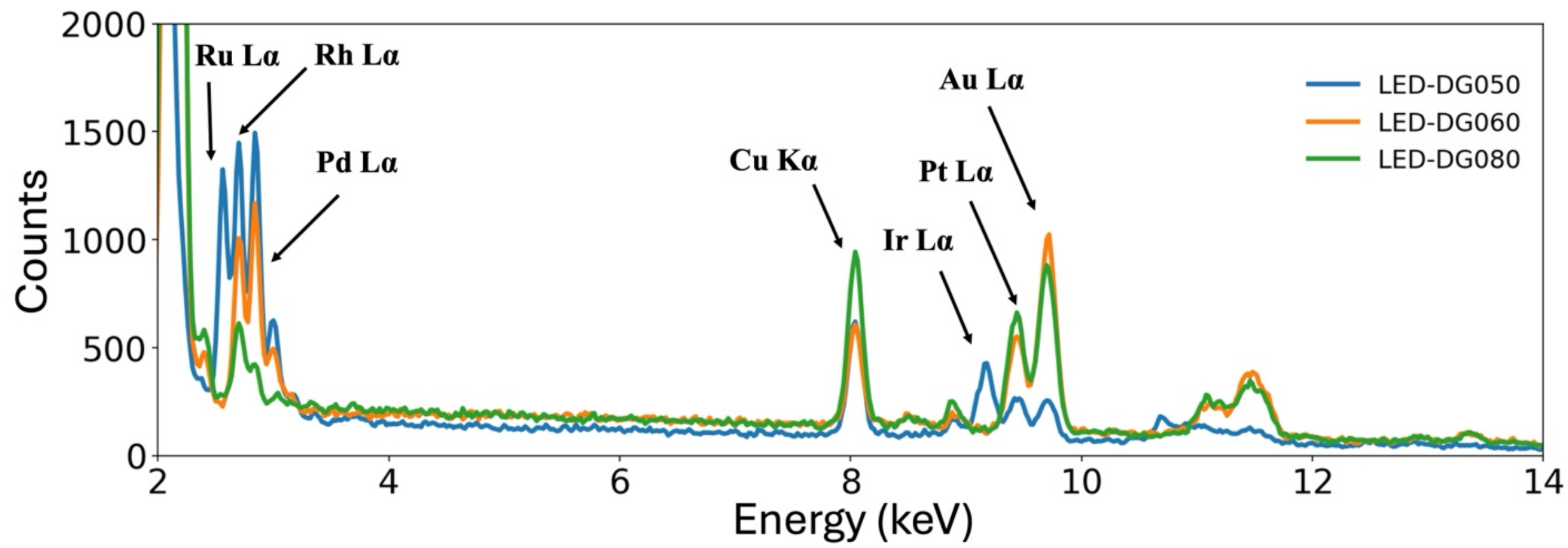

Figure S5: Representative energy-dispersive X-ray spectroscopy (EDX) datasets acquired from three different catalyst samples: LED-DG050 ($Au_{0.15}Ir_{0.23}Pt_{0.15}Pd_{0.14}Rh_{0.12}Ru_{0.21}$), LED-DG060 ($Au_{0.56}Pt_{0.24}Pd_{0.08}Rh_{0.12}$) and LED-DG080 ($Au_{0.61}Pt_{0.34}Rh_{0.05}$).

1.6 Composition control

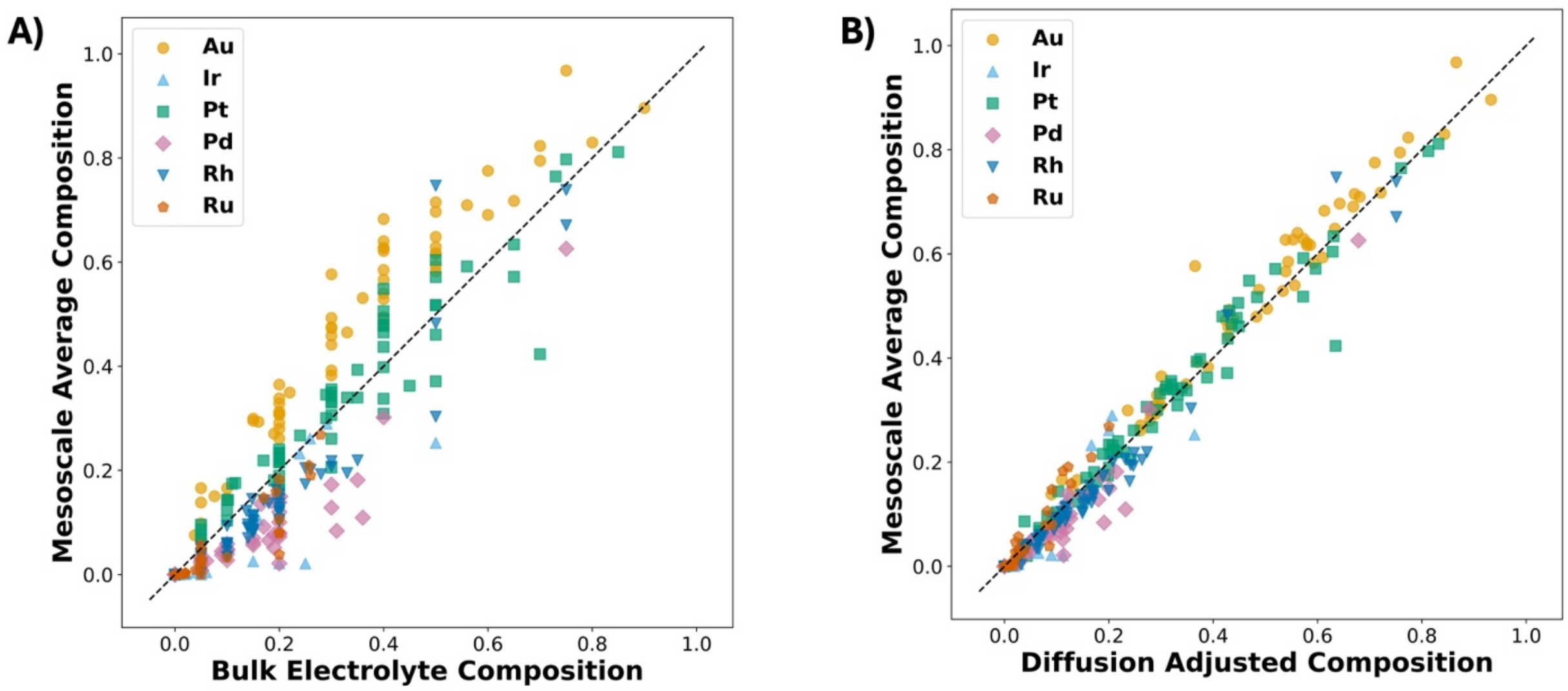

Figure S6: Comparison of metal precursor compositions in the electrolyte, and average catalyst composition obtained from the EDX analysis. A) Parity plot comparing the bulk electrolyte compositions with the (mesoscale) average compositions of the synthesized nanoparticles determined by EDX analysis. B) Parity plot comparing the (mesoscale) average EDX compositions with the corresponding diffusion-adjusted precursor compositions. The diffusion-adjusted compositions were calculated from the bulk electrolyte precursor concentrations using the metal-ion Levich constants reported in our previous work.[1]

1.7 Composition uniformity across samples

A)

B)

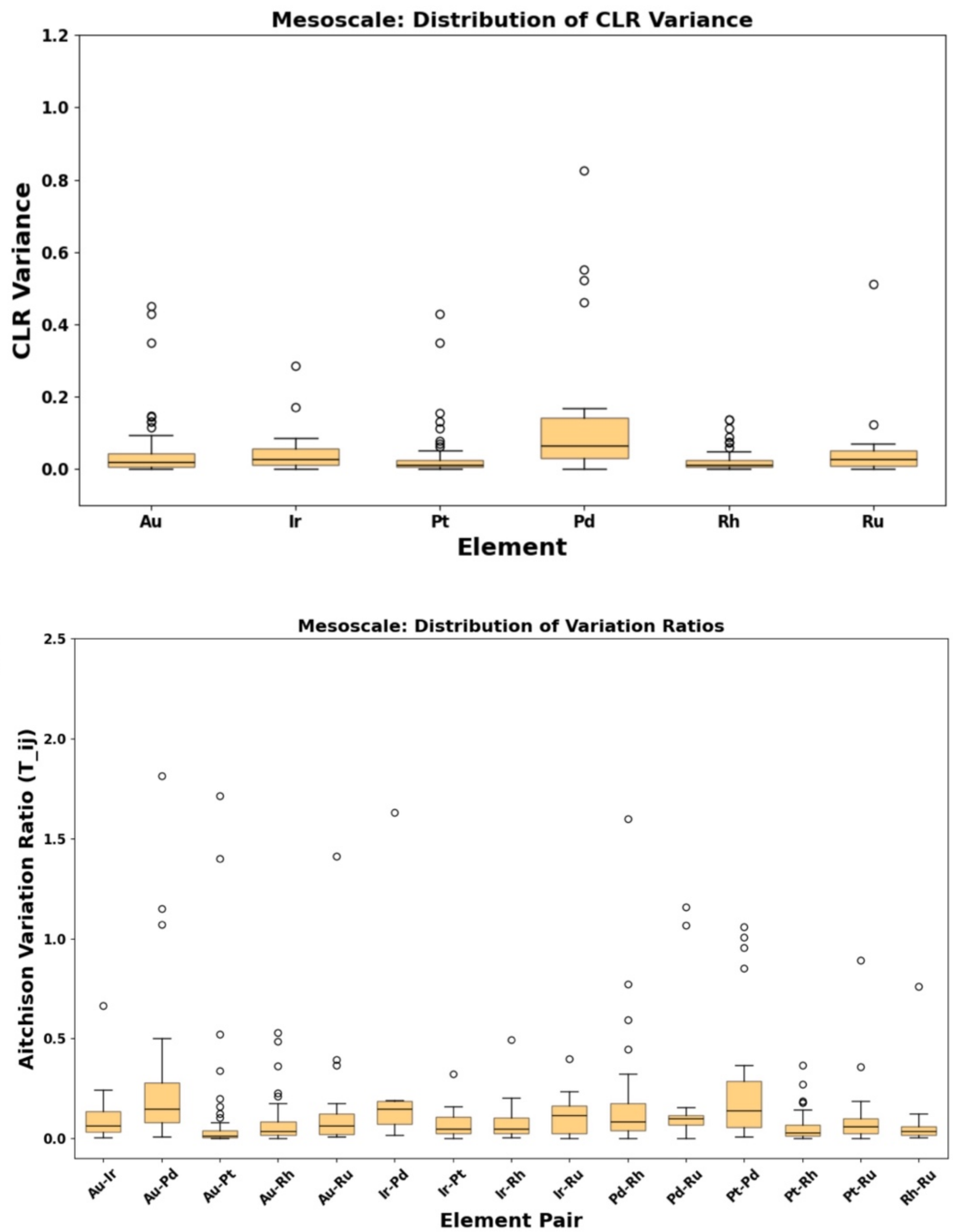

Figure S7: Compositional variability of the catalyst samples quantified using centered log-ratio (CLR) variance and the Aitchison variation ratio. The CLR variance is shown for each of the six constituent elements based on the mesoscale SEM-EDX measurements from all catalyst samples (approximately 4,000 individual spectra). The Aitchison variation ratio summarizes the covariance structure of the compositional dataset.

1.8 Gaussian process regressor: training surrogate model

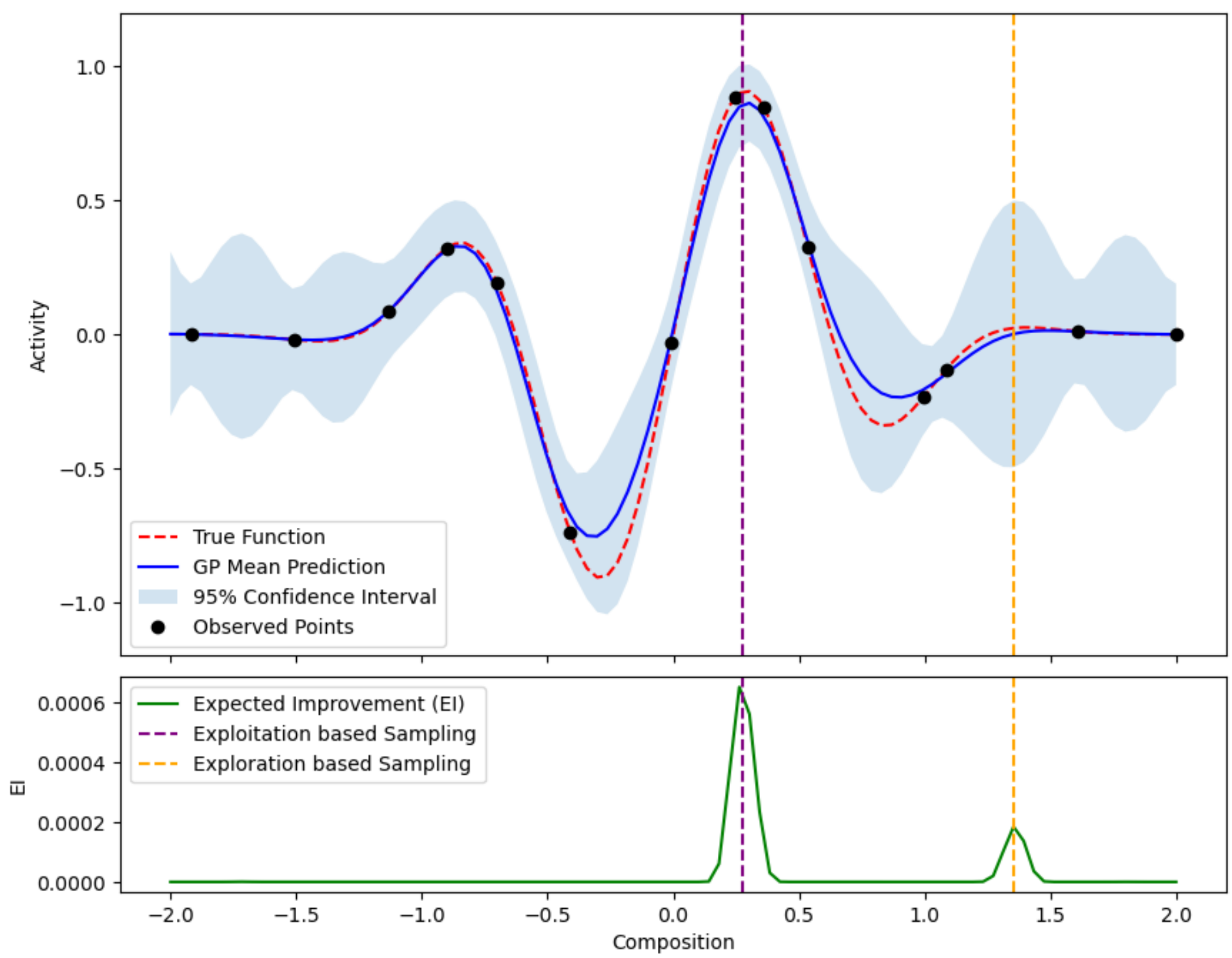

Figure S8: Schematic illustration of the Bayesian optimization workflow. The upper panel shows Gaussian process regression (GPR) used to construct the surrogate model from the experimental data. The lower panel illustrates candidate selection using the Expected Improvement (EI) acquisition

function, which balances exploration of uncertain regions and exploitation of compositions predicted to exhibit high performance.

2. Effect of glucose concentration on the measured current - time

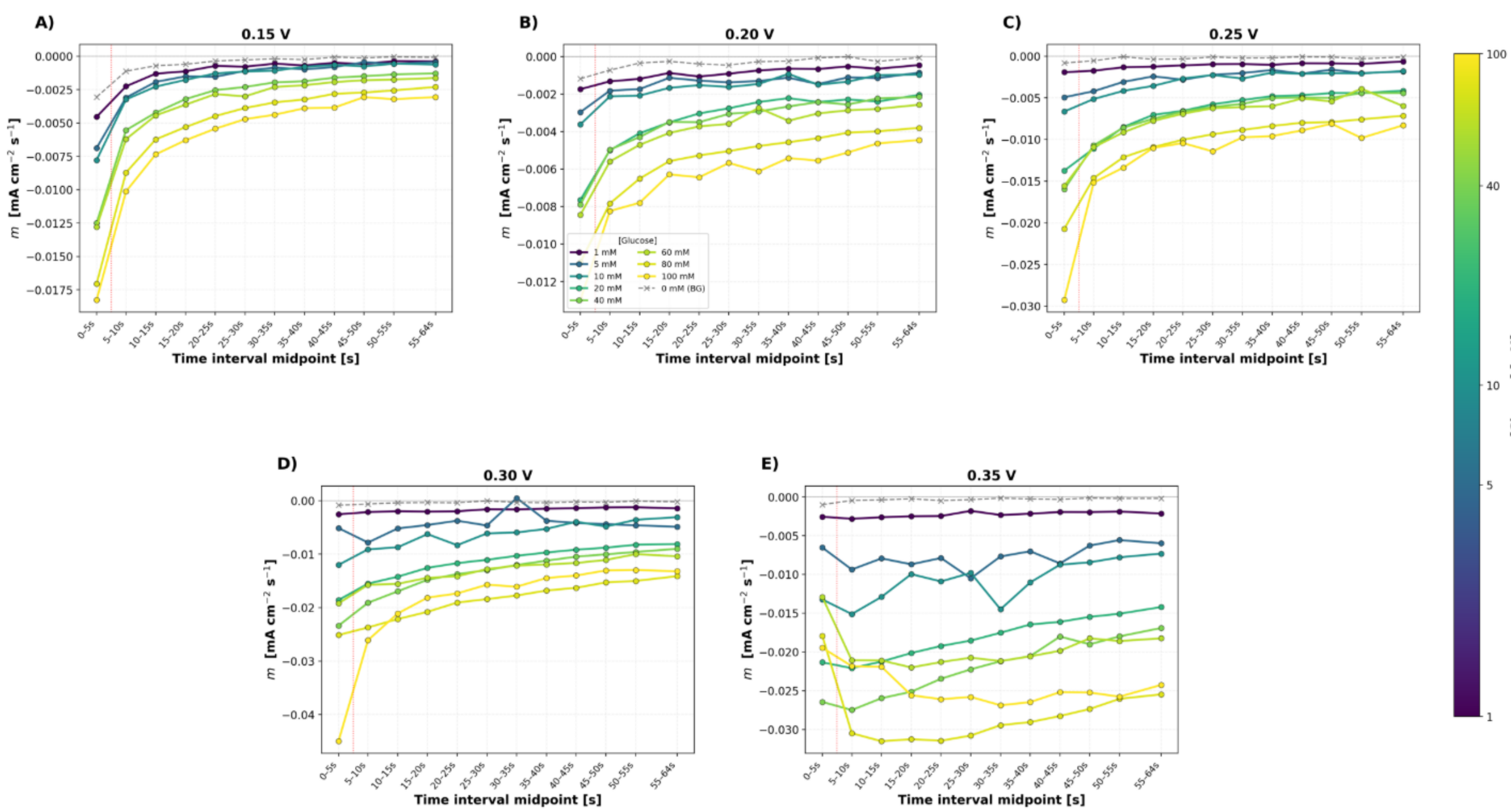

Figure S9. First derivative of the current–time response at different glucose concentrations. The first derivative of the faradaic current with respect to time is shown for the different potential steps for the catalyst composition $Au_{0.61}Pt_{0.34}Rh_{0.05}$. The measurements were performed at varying glucose concentrations using a glassy carbon (GC) rotating disk electrode operated at 2500 rpm in Ar-saturated 1 M $NaClO_4$ (pH 12).

3. *Effect of different synthesis times on the Activity objective*

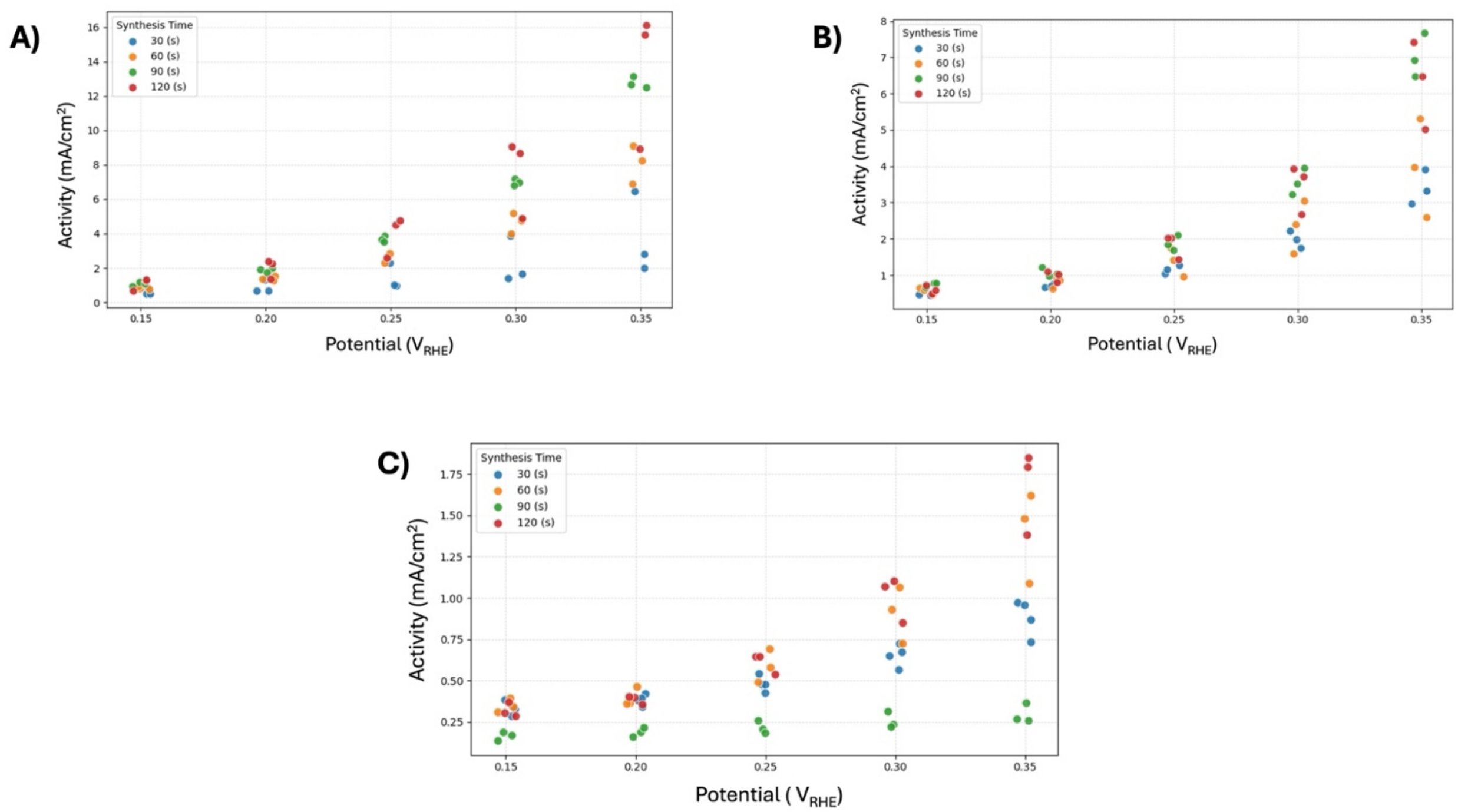

Figure S10: Influence of deposition time on the observed catalyst activity. Electrochemical activity of A) $Au_{0.61}Pt_{0.34}Rh_{0.05}$, B) $Au_{0.56}Pt_{0.24}Pd_{0.08}Rh_{0.12}$ and C) $Au_{0.15}Ir_{0.23}Pt_{0.15}Pd_{0.14}Rh_{0.12}Ru_{0.21}$ prepared using different electrodeposition times. For each deposition time, three independent samples were synthesized from the same precursor electrolyte composition.

4. *Catalyst sensitivity and analyte selectivity: Glucose electrochemical oxidation*

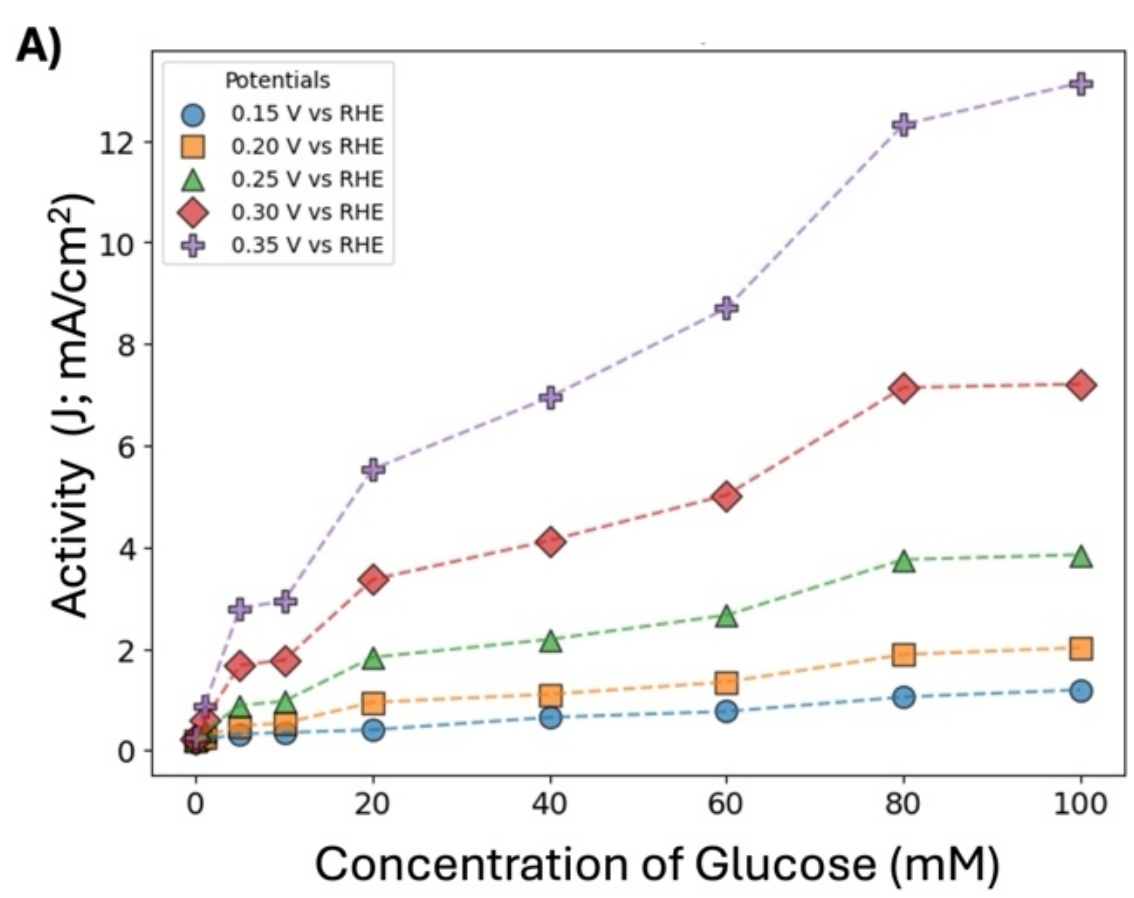

Figure S11: Glucose concentration dependence of the electrocatalytic GOR activity. Faradaic current responses were recorded at different glucose concentrations for the catalyst composition $Au_{0.61}Pt_{0.34}Rh_{0.05}$ at the five applied potentials. Measurements were performed using a glassy carbon rotating disk electrode operated at 2500 rpm in Ar-saturated 1 M $NaClO_4$ (pH 12).

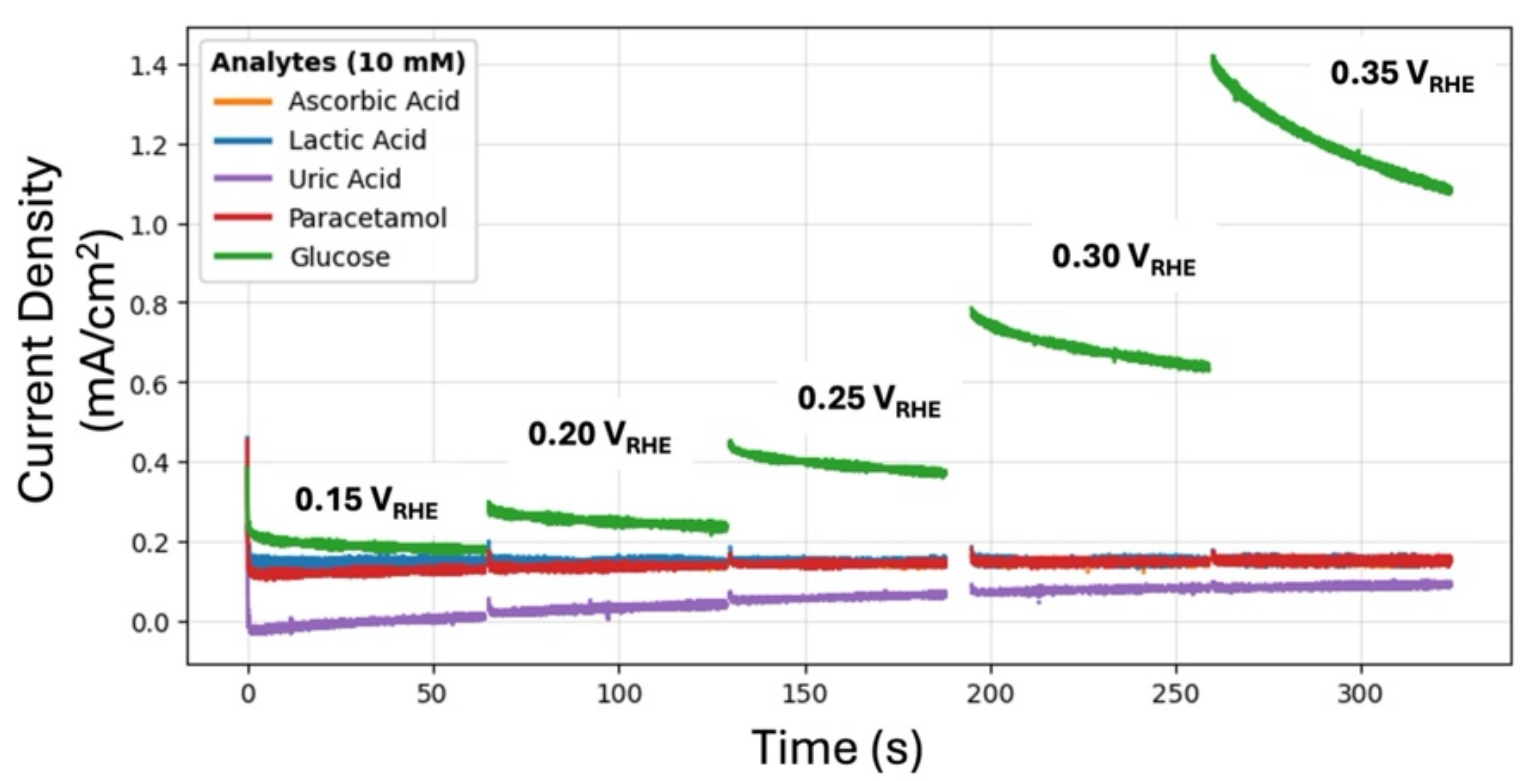

Figure S12: Selectivity of the catalyst toward glucose electrooxidation. Faradaic current responses were recorded for five analytes (10 mM each) at five applied potentials using the catalyst composition $Au_{0.61}Pt_{0.34}Rh_{0.05}$. Measurements were performed using a rotating disk electrode operated at 2500 rpm. Because several of the analytes are acidic, a phosphate buffer was used to maintain a constant electrolyte pH for all measurements. The supporting electrolyte contained 0.2 M total phosphate, prepared from trisodium phosphate dodecahydrate ($Na_3PO_4{\cdot}12\ H_2O$) and disodium hydrogen phosphate (Na_2HPO_4), corresponding to 60.2% PO_4^{3-} and 39.8% HPO_4^{2-}.

5. *Activity and Poisoning resistance surrogates*

1. *i_0.1 s ($mA.cm^{-2}$)*: Current after 0.1 second of the applied normalized by the nanoparticle coverage on a 5 mm GC disk
2. *Q_norm ($mC.cm^{-2}$)*: Integrated current over the time domain of 0.1 – 64 seconds
3. *Poisoning_resistance_1*: Ratio of current after 1 second to the current after 0.1 second (including penalty; see methods).
4. *Poisoning_resistance_10*: Ratio of current after 10 seconds to the current after 0.1 second.
5. *Poisoning_resistance_64*: Ratio of current after 64 seconds to the current after 0.1 second.

5.1 Activity surrogate

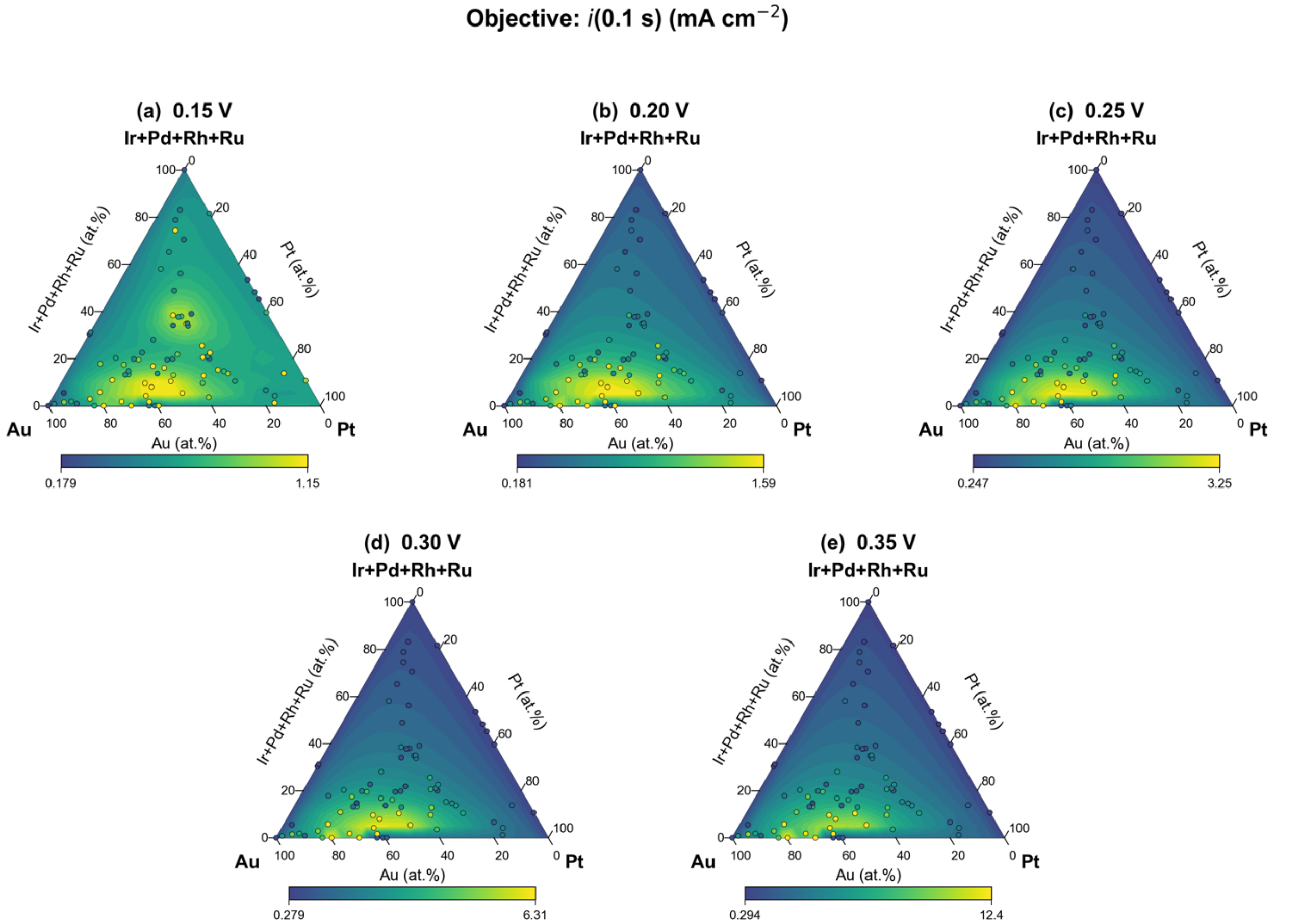

Figure S13: Gaussian process regression (GPR) surrogate models of the Au-Ir-Pt-Pd-Rh-Ru composition-activity space for the glucose oxidation reaction (GOR) activity descriptor (current density at 0.1 s) at five applied potentials. The circles indicate the experimentally measured catalyst compositions, whereas the contour maps represent the GPR-predicted activity across the projected compositional simplex. Independent surrogate models were trained for each applied potential.

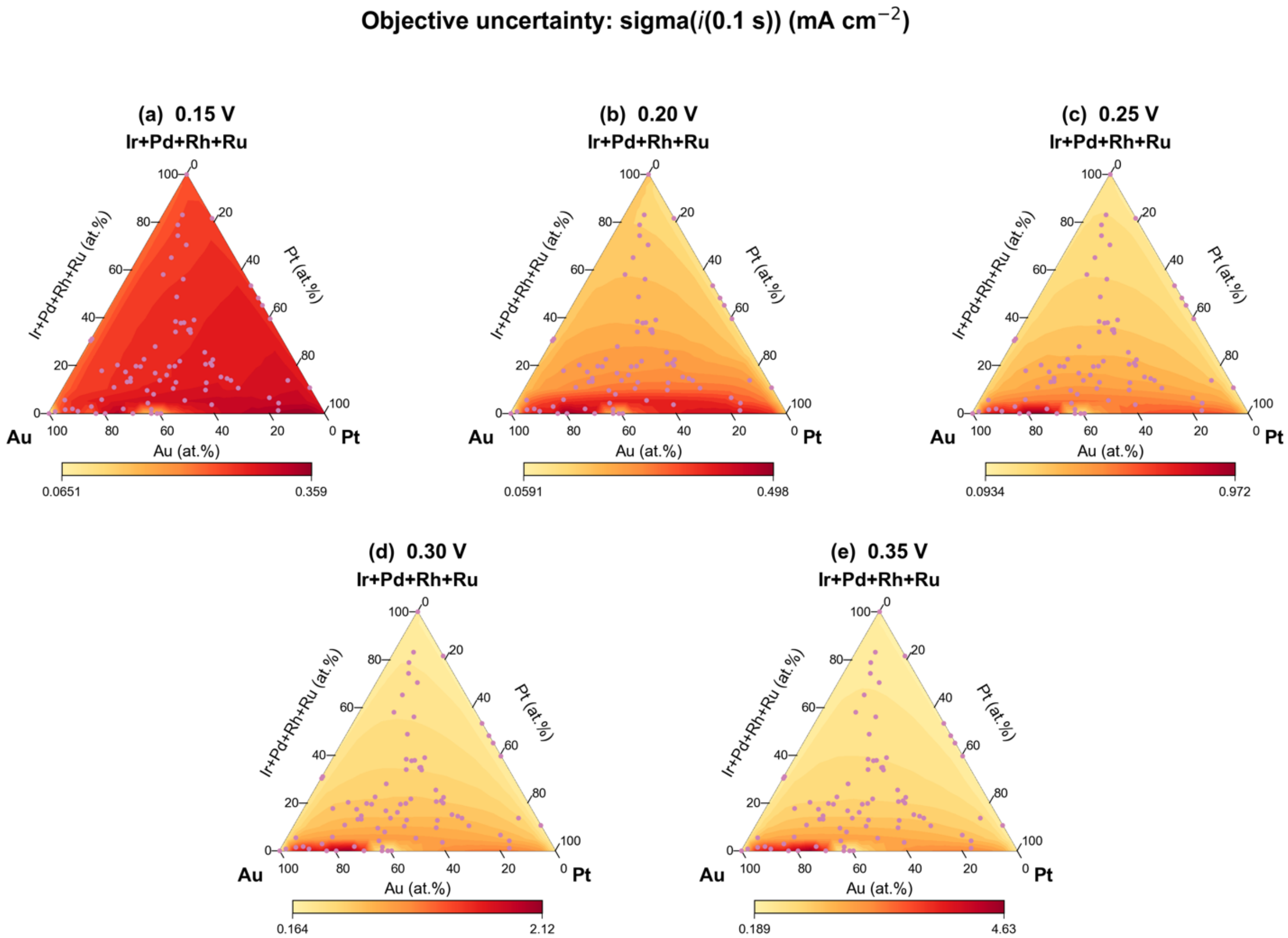

Figure S14: Gaussian process regression (GPR) uncertainty maps for the Au–Ir–Pt–Pd–Rh–Ru composition space at five applied potentials. Contour maps show the predicted absolute uncertainty associated with the glucose oxidation reaction (GOR) activity descriptor (current density after 0.1 s) across the projected compositional simplex.

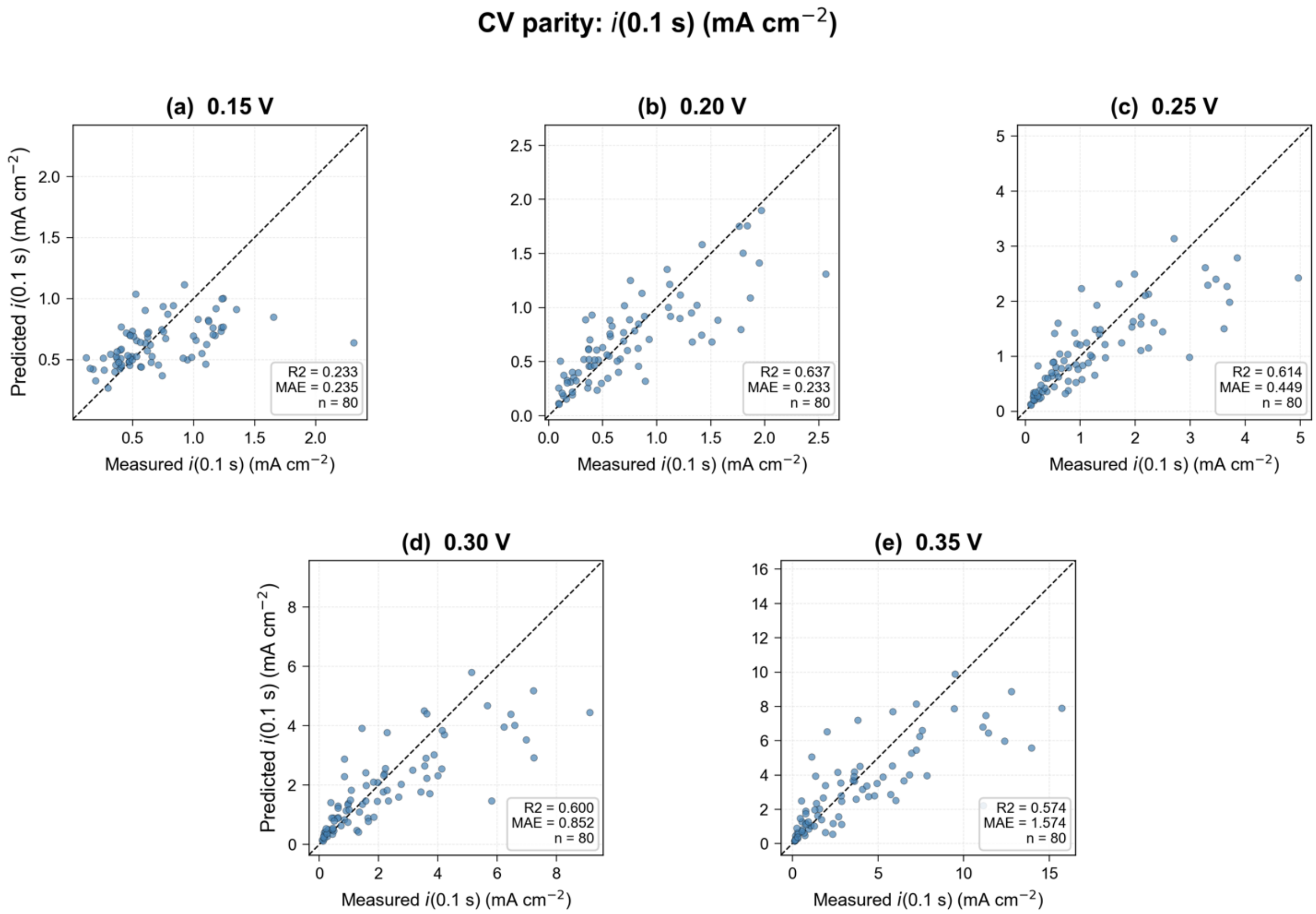

Figure S15: Leave-one-group-out cross-validation (LOGO-CV) parity plots evaluating the predictive performance of the Gaussian process regression (GPR) surrogate models for the activity descriptor (current density after 0.1 s) at the five applied potentials for glucose oxidation.

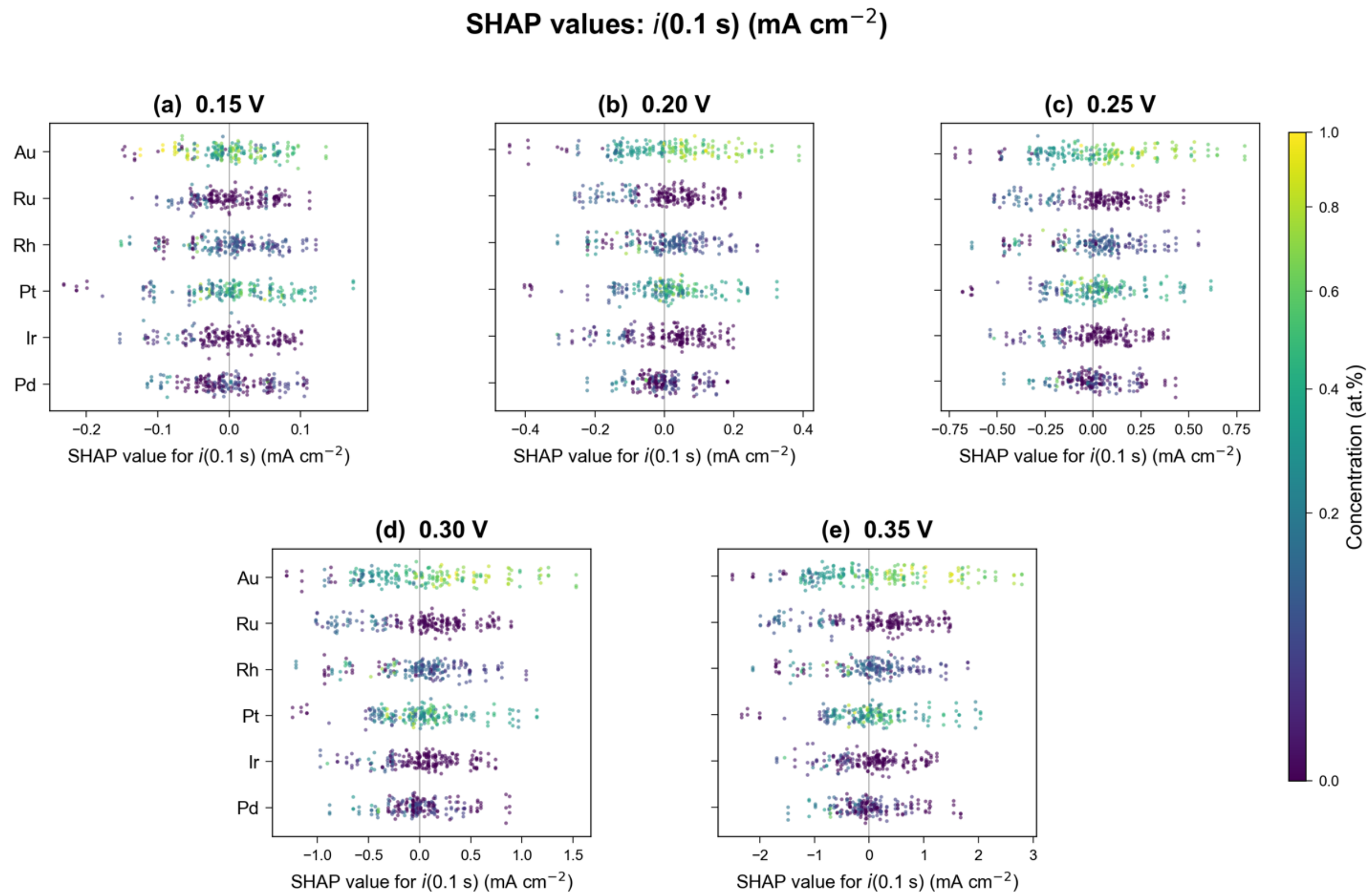

Figure S16: SHAP (Shapley additive explanations) summary plots showing the contributions of the six elements to the Gaussian process regression (GPR) activity surrogate models at the five applied potentials.

5.2 Poisoning resistance at different time domains

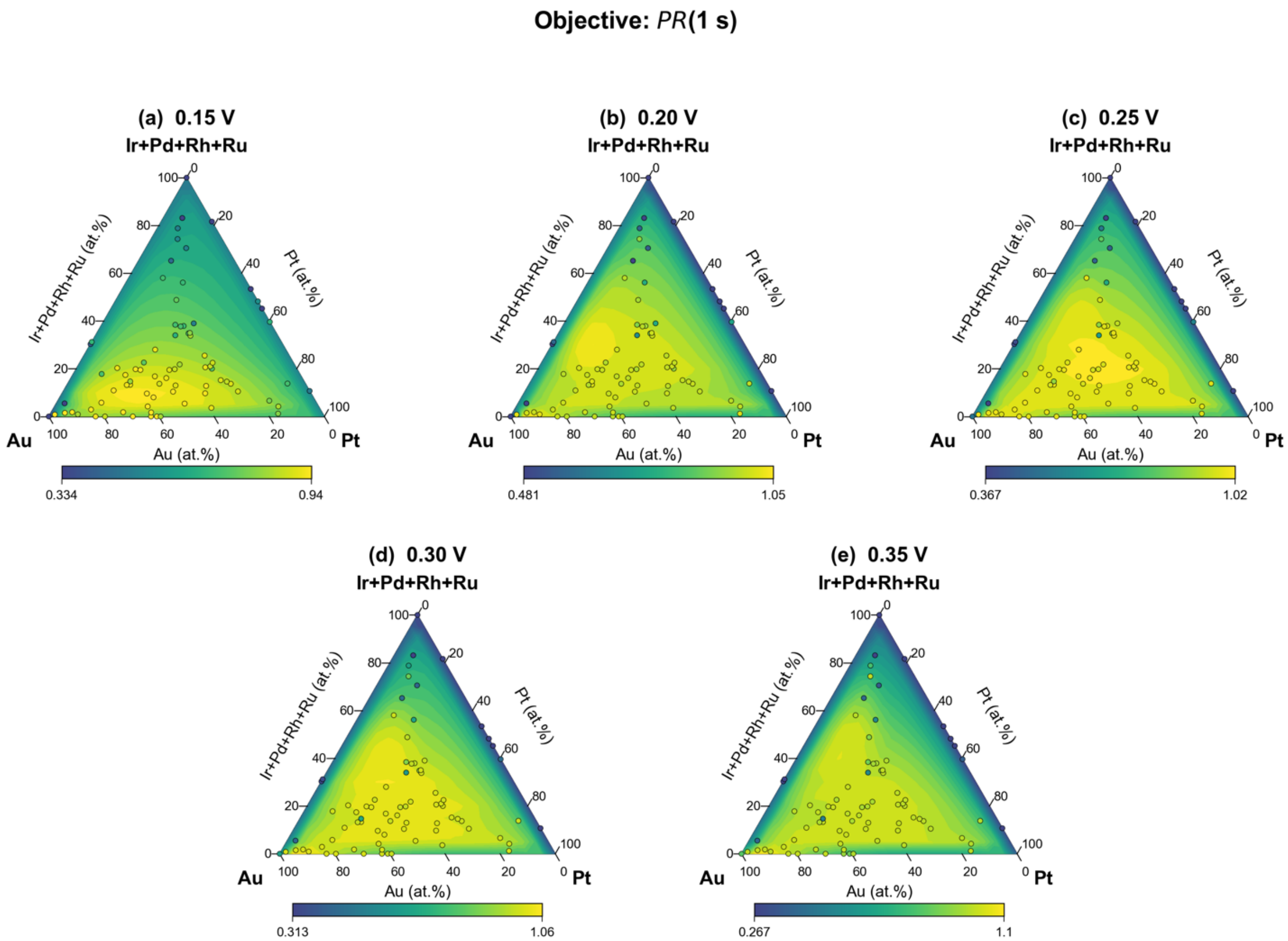

Figure S17: Gaussian process regression (GPR) surrogate maps of the Au–Ir–Pt–Pd–Rh–Ru composition–poisoning resistance landscape *PR*(1 s) at the five applied potentials for the glucose oxidation reaction (GOR).

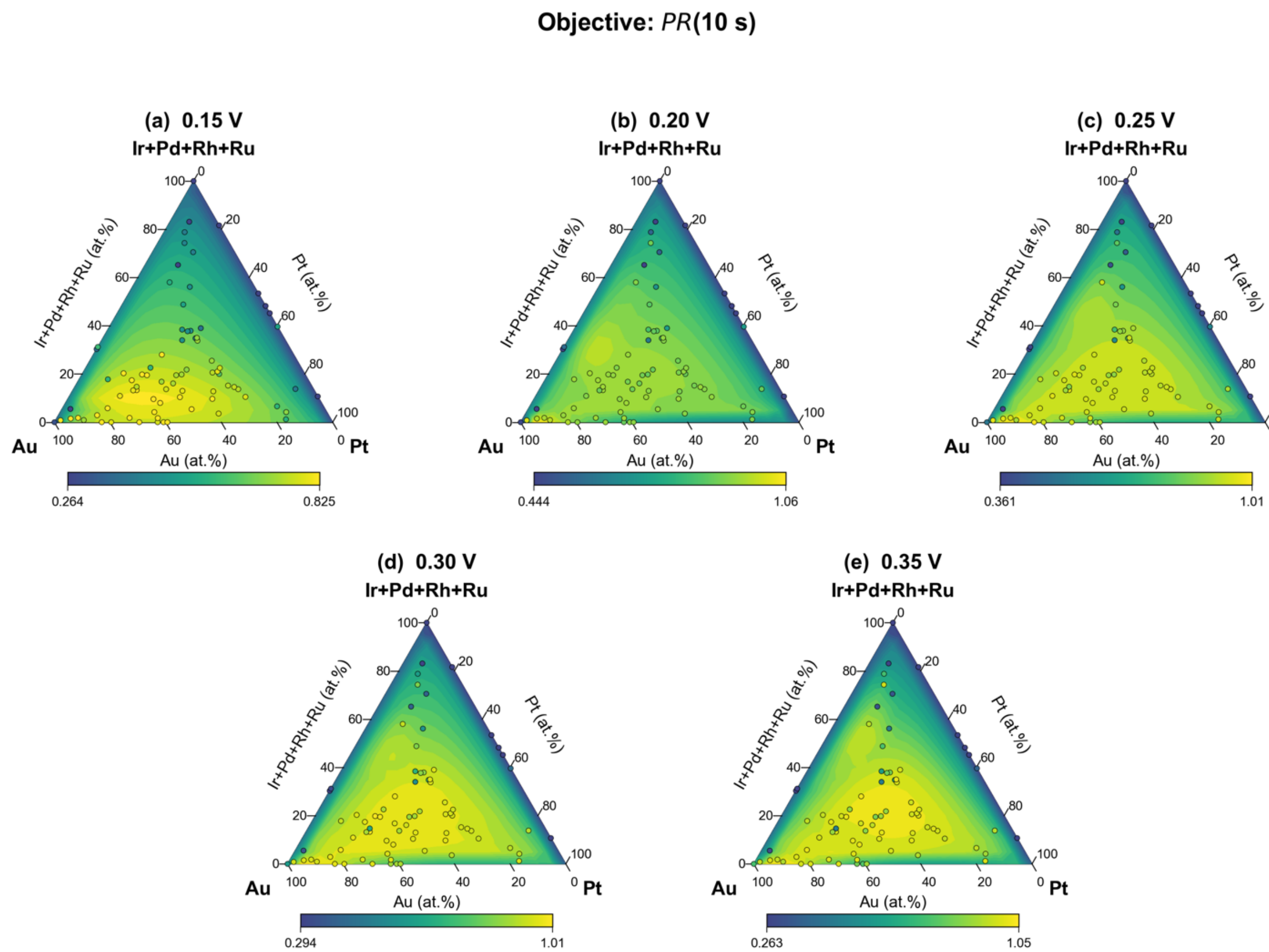

Figure S18: Gaussian process regression (GPR) surrogate maps of the Au–Ir–Pt–Pd–Rh–Ru composition–poisoning resistance landscape *PR*(10 s) at the five applied potentials for the glucose oxidation reaction (GOR).

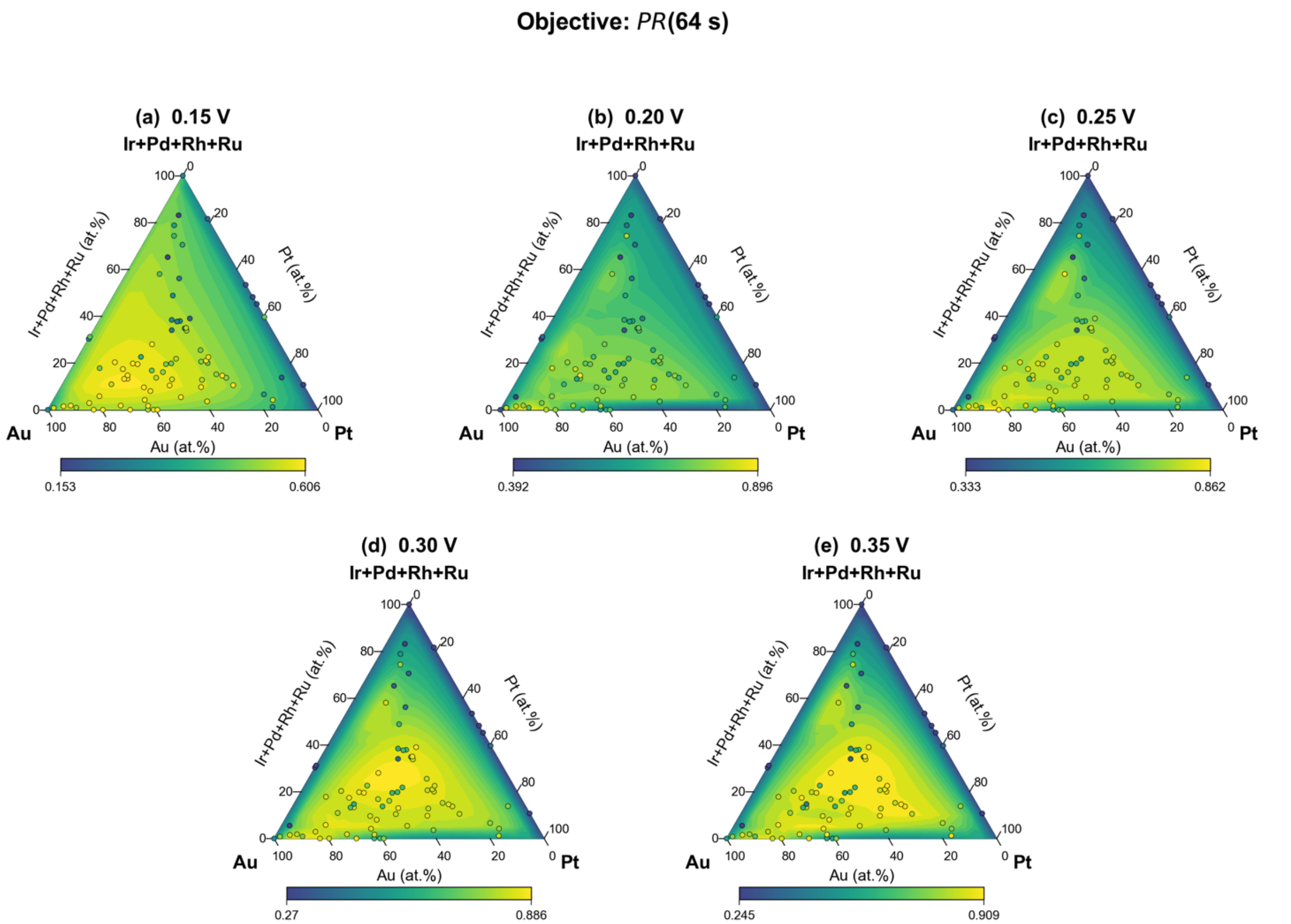

Figure S19: Gaussian process regression (GPR) surrogate maps of the Au–Ir–Pt–Pd–Rh–Ru composition–poisoning resistance landscape *PR*(64 s) at the five applied potentials for the glucose oxidation reaction (GOR).

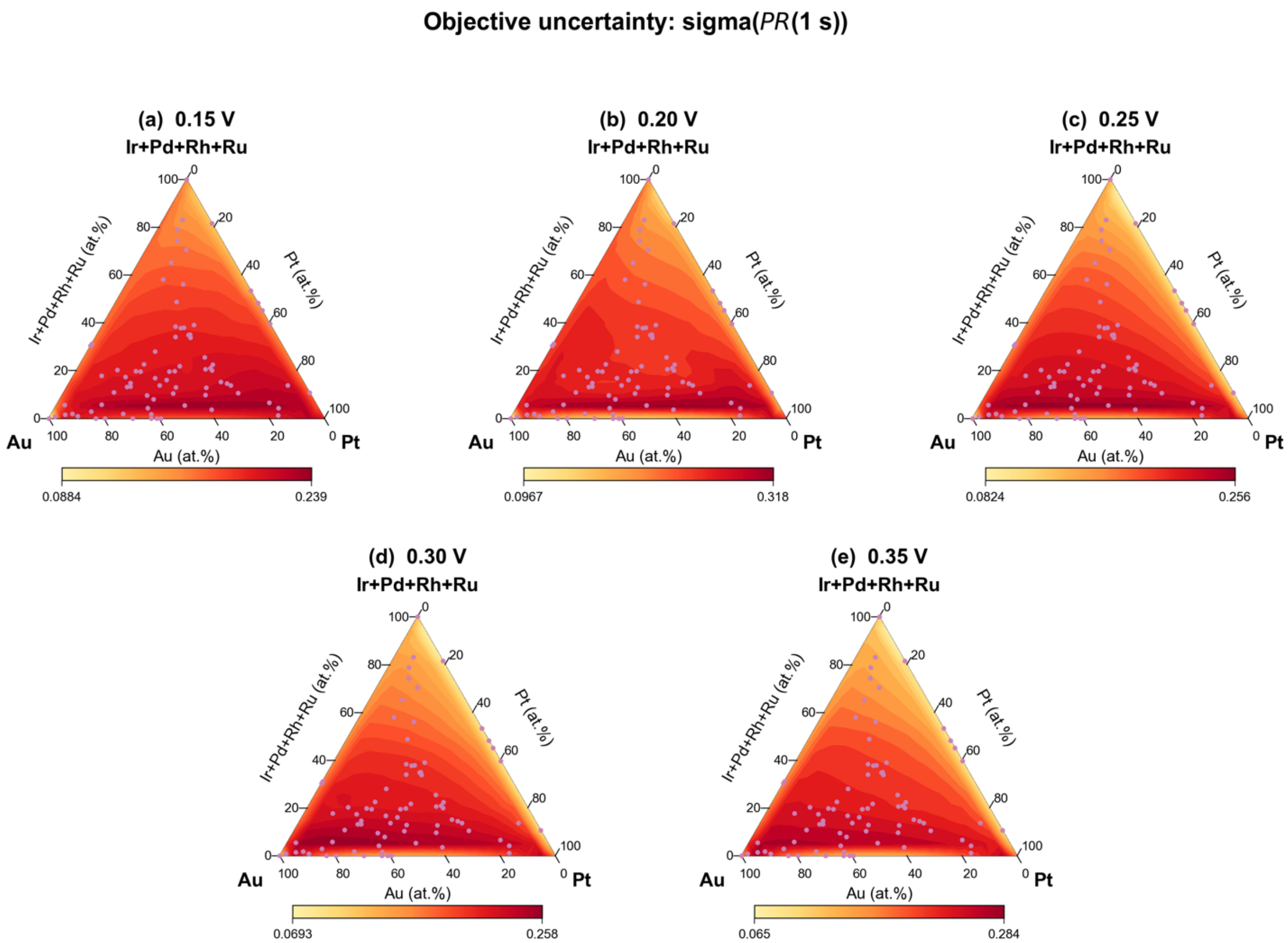

Figure S20: Gaussian process regression (GPR) uncertainty maps showing the predicted absolute uncertainty of the poisoning-resistance descriptor *PR*(1 s) across the Au–Ir–Pt–Pd–Rh–Ru compositional space at the five applied potentials for glucose oxidation reaction (GOR).

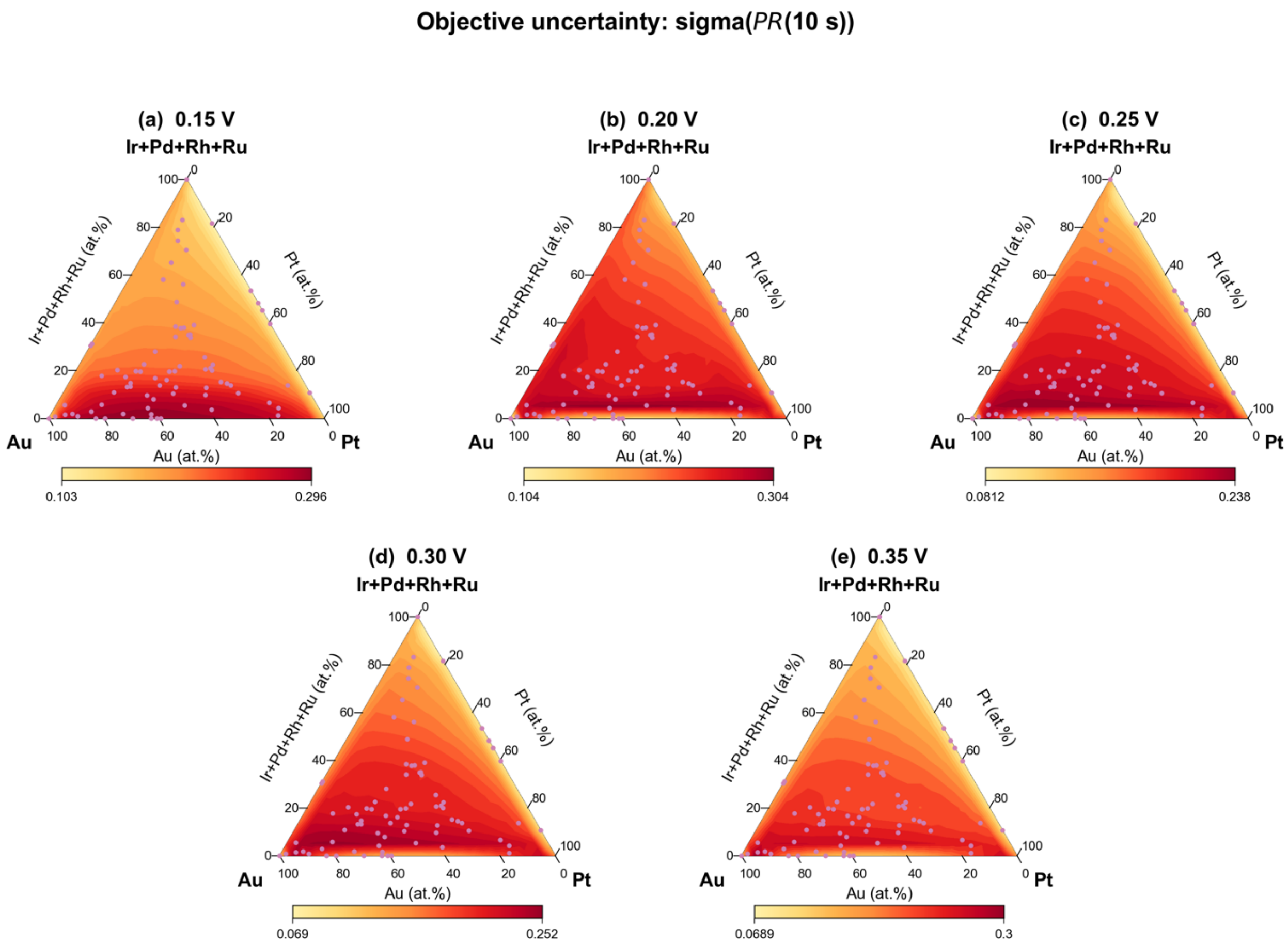

Figure S21: Gaussian process regression (GPR) uncertainty maps showing the predicted absolute uncertainty of the poisoning-resistance descriptor *PR*(10 s) across the Au–Ir–Pt–Pd–Rh–Ru compositional space at the five applied potentials for glucose oxidation reaction (GOR).

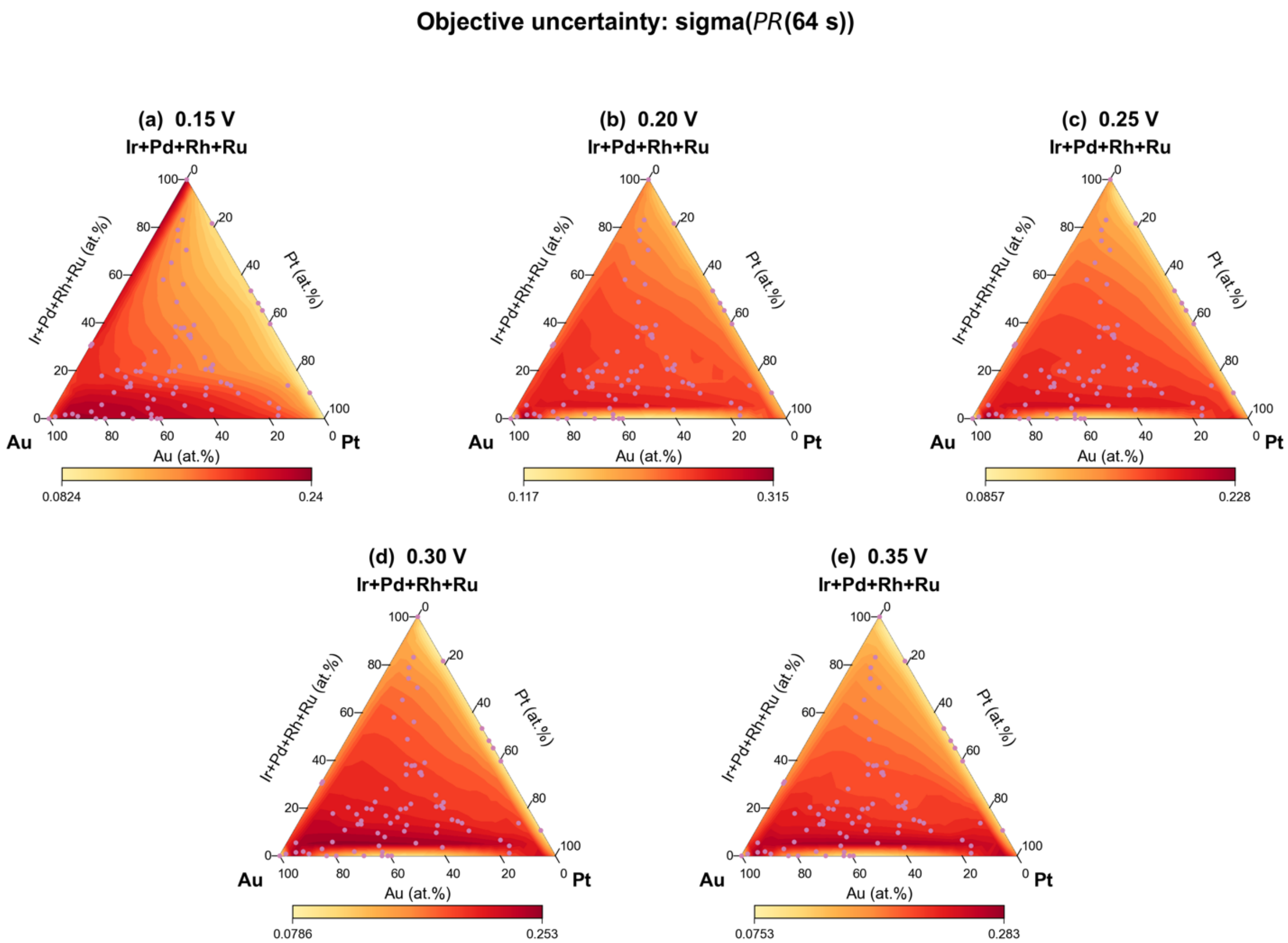

Figure S22: Gaussian process regression (GPR) uncertainty maps showing the predicted absolute uncertainty of the poisoning-resistance descriptor *PR*(64 s) across the Au–Ir–Pt–Pd–Rh–Ru compositional space at the five applied potentials for glucose oxidation reaction (GOR).

5.3 Cross-validation method

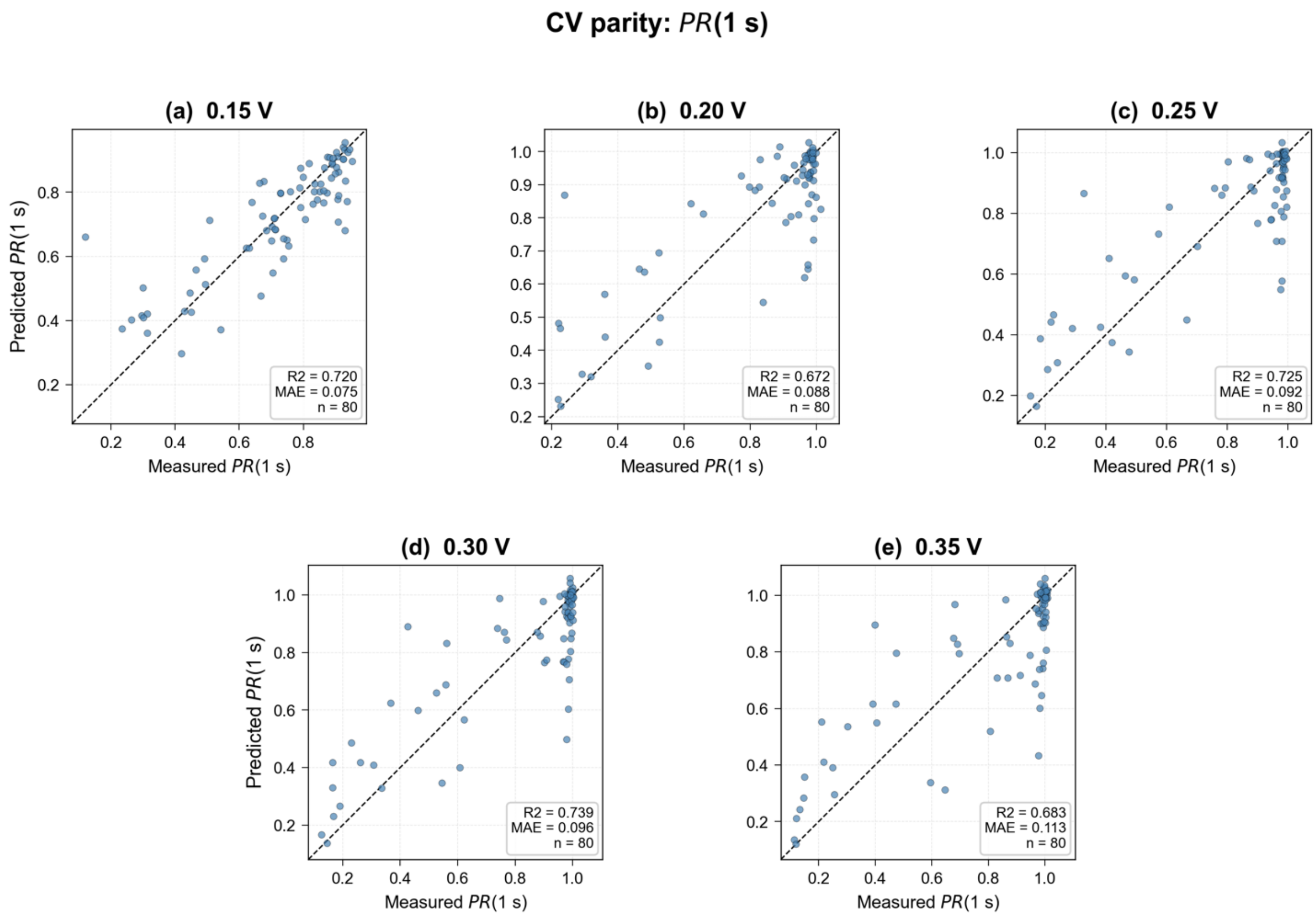

Figure S23: Leave-one-group-out cross-validation (LOGO-CV) parity plots evaluating the predictive performance of the Gaussian process regression (GPR) surrogate models for the poisoning-resistance descriptor *PR*(1 s) at the five applied potentials for glucose oxidation reaction (GOR).

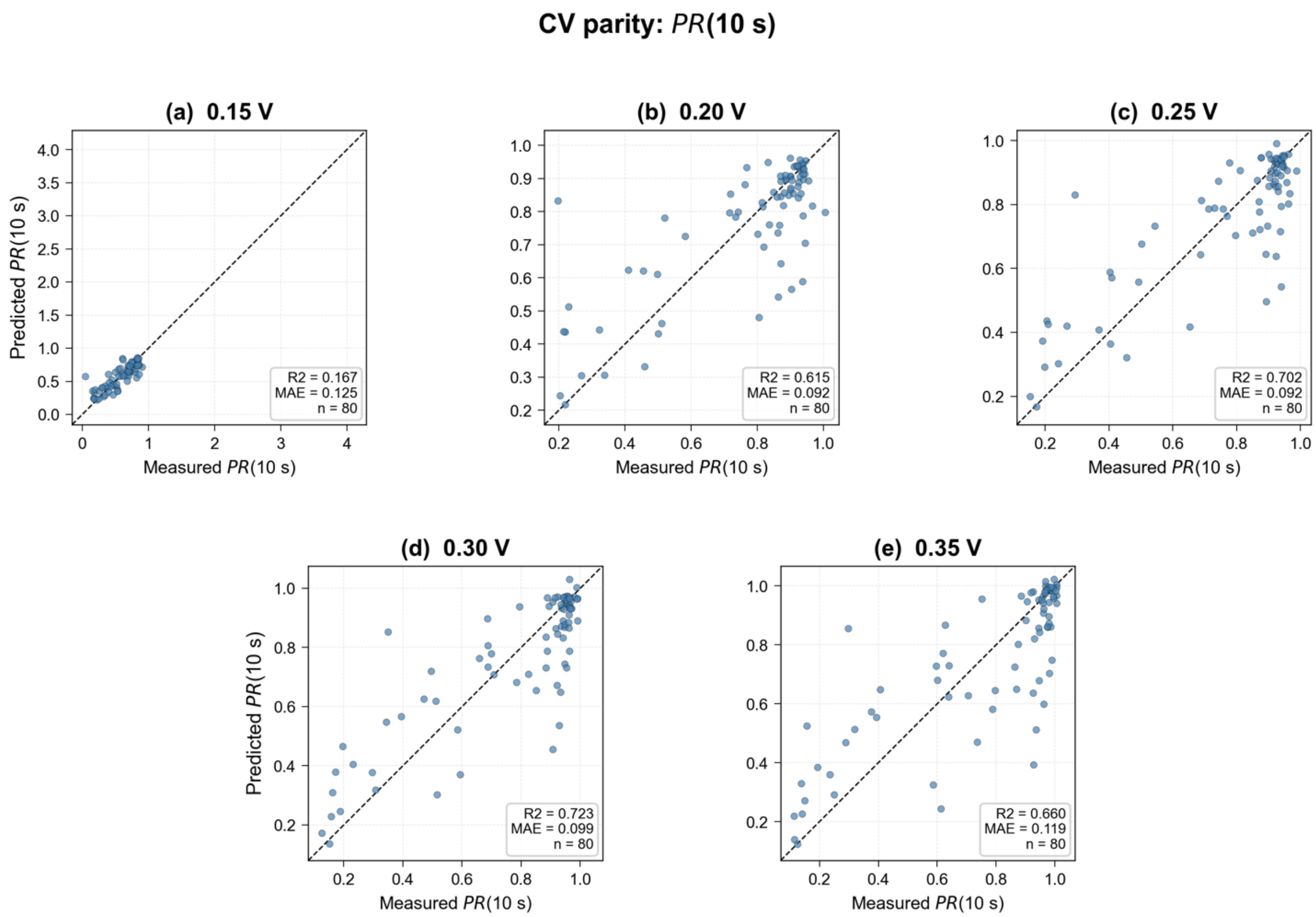

Figure S24: Leave-one-group-out cross-validation (LOGO-CV) parity plots evaluating the predictive performance of the Gaussian process regression (GPR) surrogate models for the poisoning-resistance descriptor *PR*(10 s) at the five applied potentials for glucose oxidation reaction (GOR).

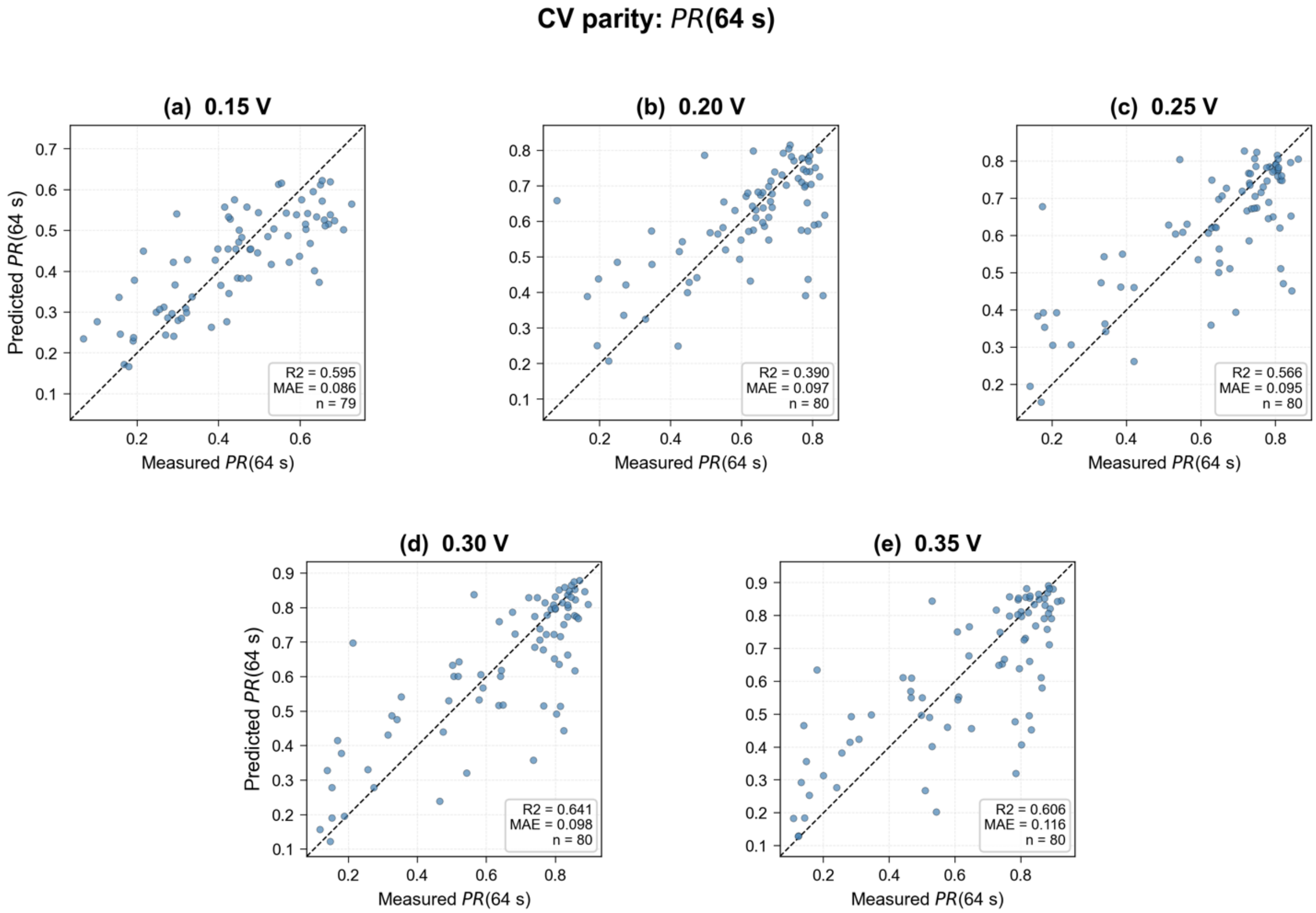

Figure S25: Leave-one-group-out cross-validation (LOGO-CV) parity plots evaluating the predictive performance of the Gaussian process regression (GPR) surrogate models for the poisoning-resistance descriptor *PR*(64 s) at the five applied potentials for glucose oxidation reaction (GOR).

5.4 *SHAP Values*

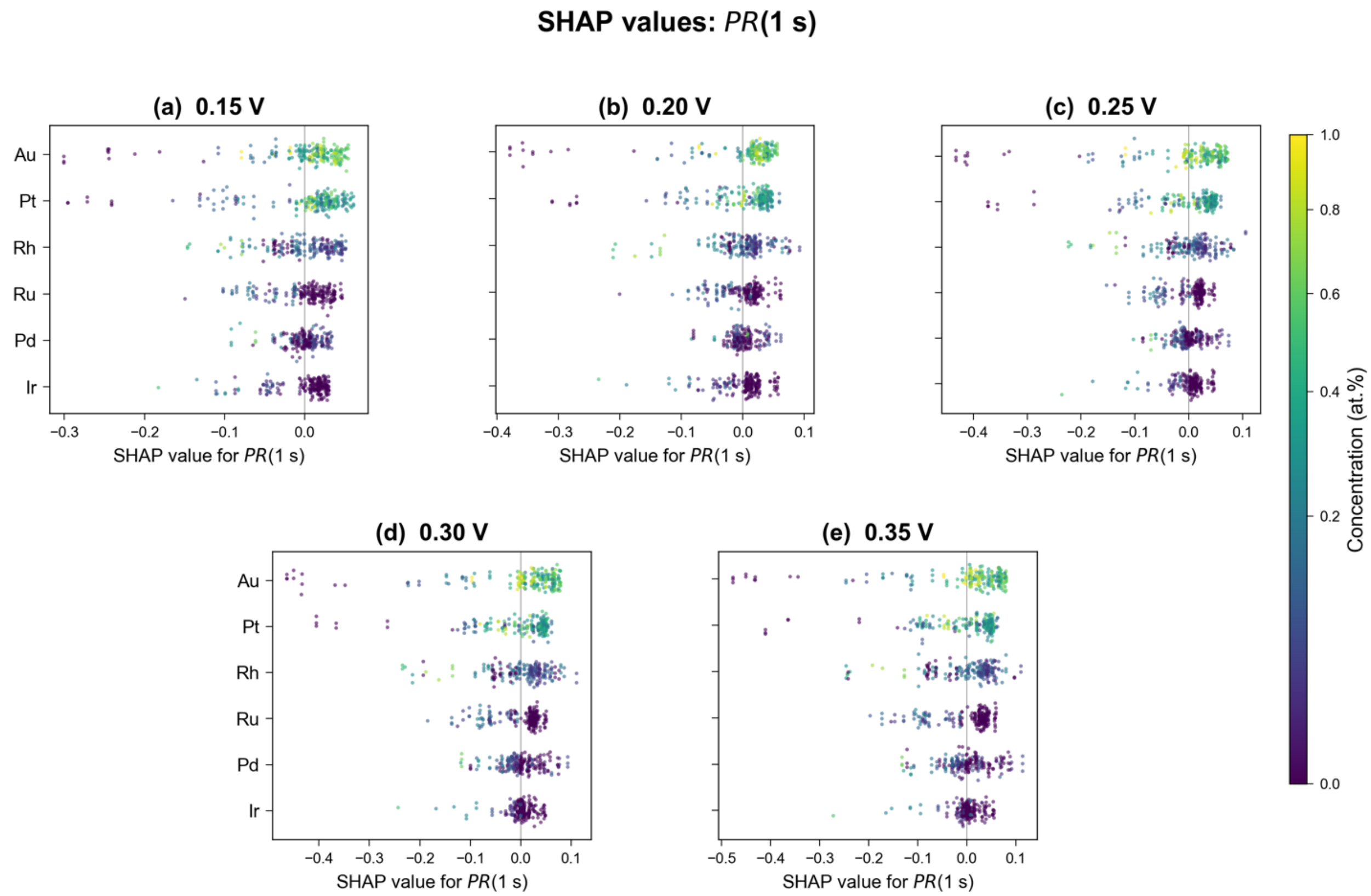

Figure S26: SHAP (Shapley additive explanations) summary plots illustrating the contributions of the six elemental descriptors to the Gaussian process regression (GPR) surrogate models for *PR*(1 s).

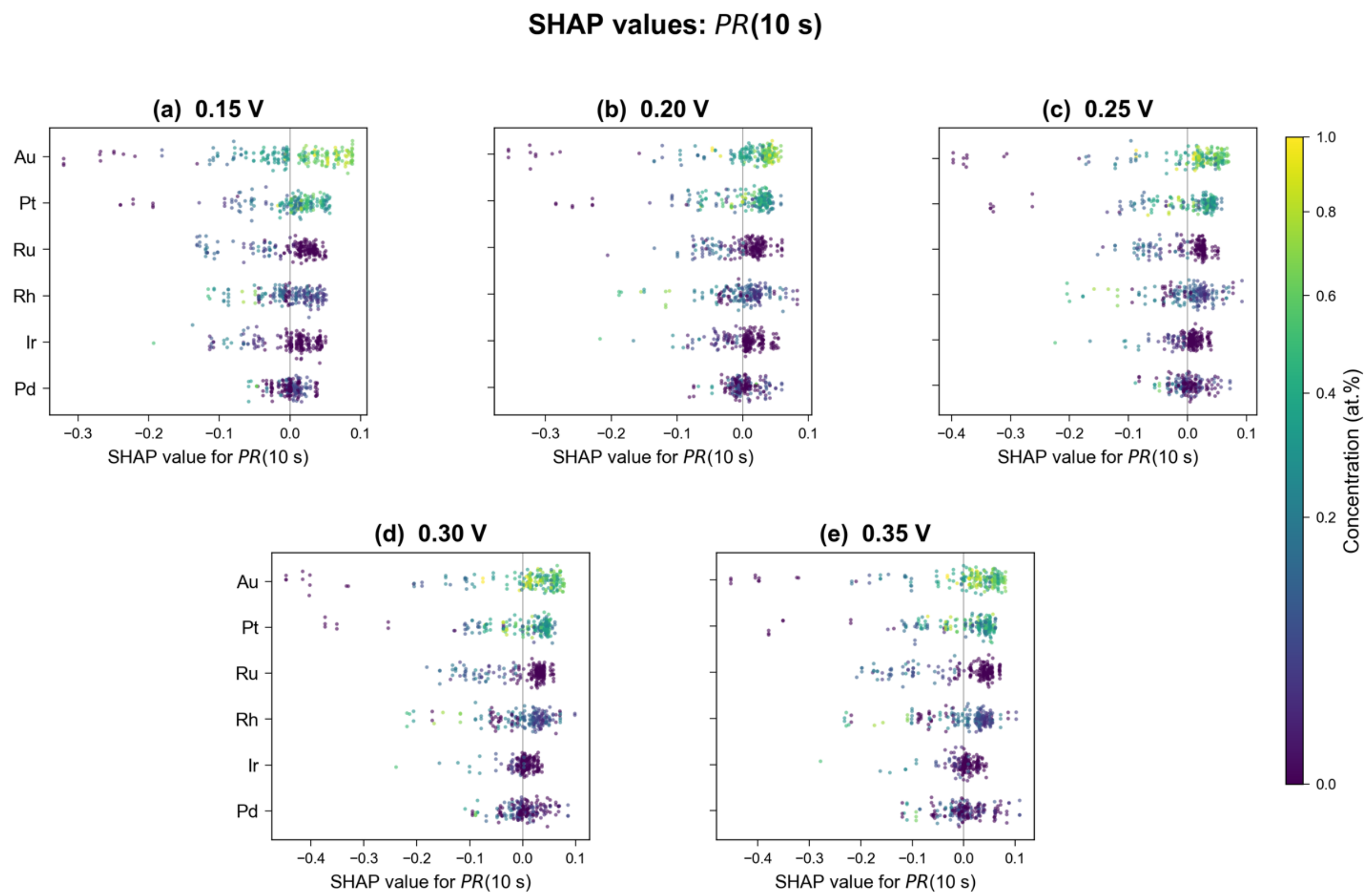

Figure S27: SHAP (Shapley additive explanations) summary plots illustrating the contributions of the six elemental descriptors to the Gaussian process regression (GPR) surrogate models for *PR*(10 s).

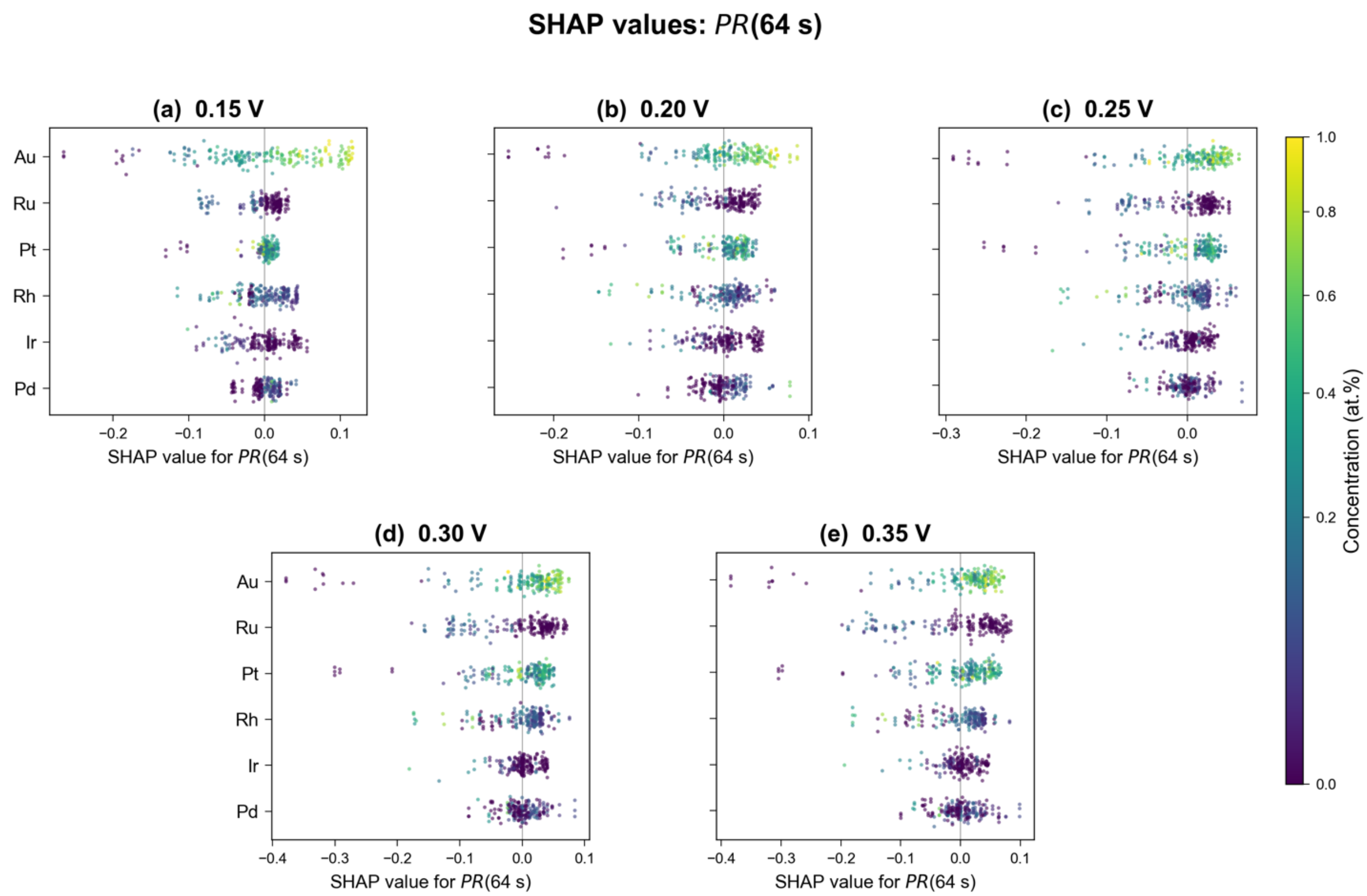

Figure S28: SHAP (Shapley additive explanations) summary plots illustrating the contributions of the six elemental descriptors to the Gaussian process regression (GPR) surrogate models for *PR*(64 s).

5.5 Top compositions at different potentials

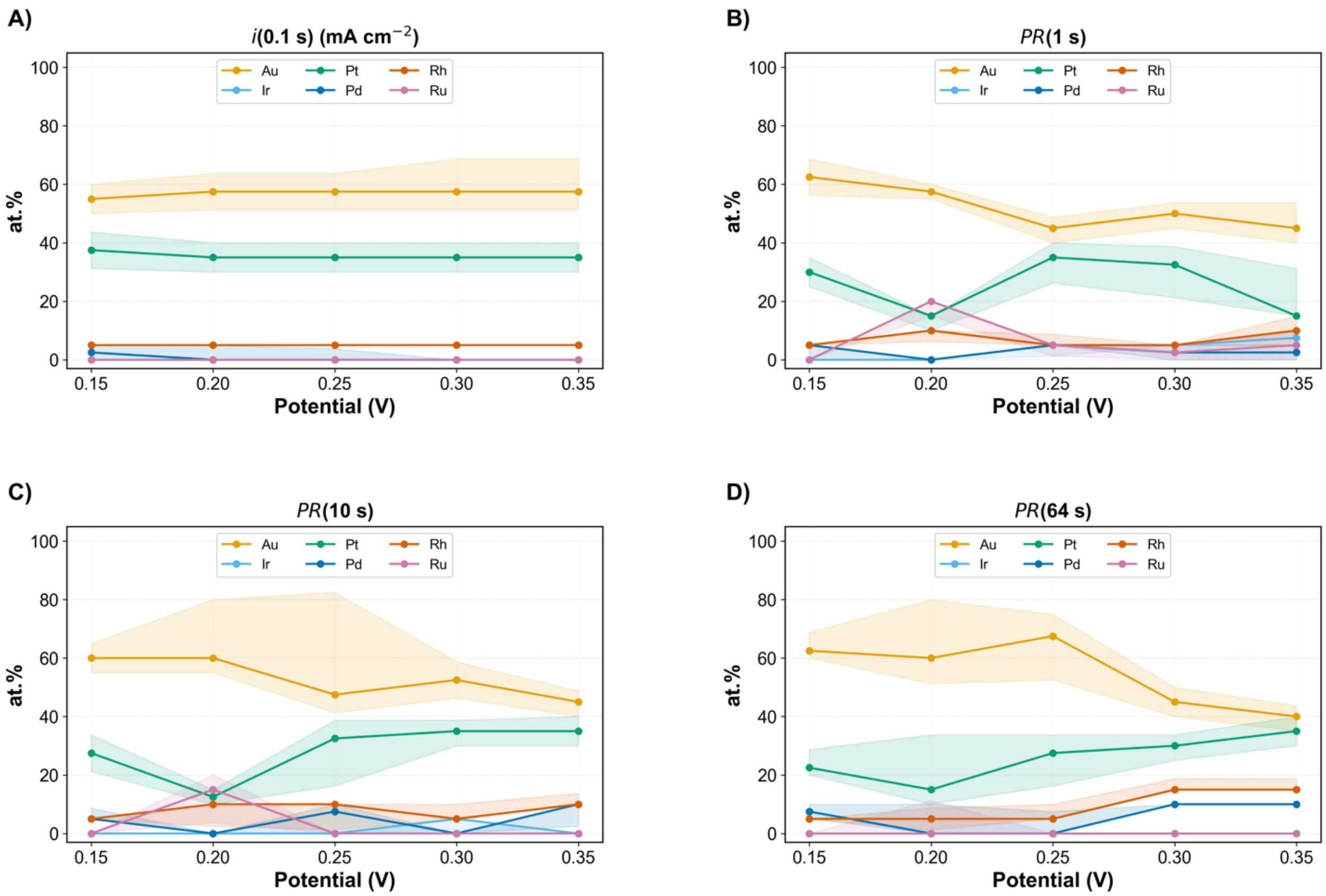

Figure S29: Potential-dependent elemental composition of the top Gaussian process regression (GPR)-predicted catalyst compositions for activity and poisoning resistance. Median elemental fractions and interquartile ranges (IQRs) of the ten highest-ranked compositions at each applied potential are shown for (A) initial activity, i(0.1 s), (B) poisoning resistance PR(1 s), (C) PR(10 s), and (D) PR(64 s). For each objective and applied potential, an independently optimized GPR surrogate model was evaluated over the six-component Au–Ir–Pt–Pd–Rh–Ru compositional simplex using a 5 at.% grid spacing. The ten compositions with the highest predicted response were subsequently identified. Solid lines represent the median elemental fraction, whereas shaded regions indicate the interquartile range (25th–75th percentile). The activity objective exhibits a nearly potential-independent Au–Pt-rich optimum, whereas the poisoning-resistance objectives display stronger potential and time dependence, with longer retention times favouring Au/Pt-rich compositions containing smaller fractions of Pd and Rh.

6. *Pareto optimals*

Table S1: Pareto-optimal catalyst compositions for glucose oxidation corresponding to Figure 5, together with the predicted objective values obtained from the Gaussian process regression (GPR) surrogate models evaluated on a 5 at.% compositional grid.

ID	Au	Ir	Pt	Pd	Rh	Ru	Potential	Pareto vs	GPR i	GPR PR1	GPR PR10	GPR PR64
1	55.0	0.0	35.0	5.0	5.0	0.0	0.15V	PR(1 s)	1.153	0.934	0.813	0.595
2	60.0	0.0	30.0	5.0	5.0	0.0	0.15V	PR(1 s)	1.114	0.939	0.816	0.601
3	65.0	0.0	25.0	5.0	5.0	0.0	0.15V	PR(1 s)	1.017	0.940	0.815	0.605
4	60.0	0.0	35.0	0.0	5.0	0.0	0.20V	PR(1 s)	1.588	0.991	0.938	0.783
5	65.0	0.0	30.0	0.0	5.0	0.0	0.20V	PR(1 s)	1.522	0.995	0.943	0.782
6	70.0	0.0	25.0	0.0	5.0	0.0	0.20V	PR(1 s)	1.400	0.999	0.948	0.781
7	75.0	0.0	20.0	0.0	5.0	0.0	0.20V	PR(1 s)	1.267	1.000	0.950	0.778
8	60.0	0.0	25.0	10.0	5.0	0.0	0.20V	PR(1 s)	1.054	1.004	0.943	0.785
9	55.0	0.0	30.0	10.0	5.0	0.0	0.20V	PR(1 s)	1.051	1.006	0.945	0.787
10	55.0	0.0	25.0	15.0	5.0	0.0	0.20V	PR(1 s)	0.876	1.009	0.945	0.783
11	50.0	0.0	30.0	15.0	5.0	0.0	0.20V	PR(1 s)	0.863	1.011	0.947	0.784
12	90.0	0.0	10.0	0.0	0.0	0.0	0.20V	PR(1 s)	0.809	1.037	1.024	0.896
13	95.0	0.0	5.0	0.0	0.0	0.0	0.20V	PR(1 s)	0.667	1.054	1.058	0.854
14	60.0	0.0	35.0	0.0	5.0	0.0	0.25V	PR(1 s)	3.248	0.992	0.939	0.810
15	65.0	0.0	30.0	0.0	5.0	0.0	0.25V	PR(1 s)	3.078	0.994	0.943	0.816
16	55.0	0.0	35.0	5.0	5.0	0.0	0.25V	PR(1 s)	2.838	0.994	0.938	0.774
17	70.0	0.0	25.0	0.0	5.0	0.0	0.25V	PR(1 s)	2.765	0.995	0.946	0.818
18	55.0	0.0	30.0	5.0	10.0	0.0	0.25V	PR(1 s)	2.075	0.999	0.943	0.778
19	50.0	0.0	35.0	5.0	10.0	0.0	0.25V	PR(1 s)	2.034	1.002	0.947	0.784
20	45.0	0.0	40.0	5.0	10.0	0.0	0.25V	PR(1 s)	1.939	1.003	0.947	0.791
21	90.0	0.0	10.0	0.0	0.0	0.0	0.25V	PR(1 s)	1.504	1.011	1.007	0.862
22	40.0	5.0	40.0	5.0	5.0	5.0	0.25V	PR(1 s)	0.618	1.020	0.898	0.619
23	80.0	0.0	20.0	0.0	0.0	0.0	0.30V	PR(1 s)	6.308	0.952	0.915	0.804
24	60.0	0.0	35.0	0.0	5.0	0.0	0.30V	PR(1 s)	6.121	0.998	0.956	0.844
25	65.0	0.0	30.0	0.0	5.0	0.0	0.30V	PR(1 s)	5.777	1.001	0.962	0.853
26	70.0	0.0	25.0	0.0	5.0	0.0	0.30V	PR(1 s)	5.144	1.003	0.967	0.855
27	75.0	0.0	20.0	0.0	5.0	0.0	0.30V	PR(1 s)	4.501	1.004	0.969	0.849
28	55.0	0.0	30.0	5.0	10.0	0.0	0.30V	PR(1 s)	3.664	1.005	0.969	0.821
29	50.0	0.0	35.0	5.0	10.0	0.0	0.30V	PR(1 s)	3.599	1.008	0.971	0.826
30	45.0	0.0	40.0	5.0	10.0	0.0	0.30V	PR(1 s)	3.432	1.008	0.970	0.833
31	90.0	0.0	10.0	0.0	0.0	0.0	0.30V	PR(1 s)	2.849	1.046	1.014	0.878
32	95.0	0.0	5.0	0.0	0.0	0.0	0.30V	PR(1 s)	2.323	1.056	1.009	0.825
33	80.0	0.0	20.0	0.0	0.0	0.0	0.35V	PR(1 s)	12.413	0.956	0.934	0.807
34	60.0	0.0	35.0	0.0	5.0	0.0	0.35V	PR(1 s)	10.724	1.006	0.992	0.890
35	65.0	0.0	30.0	0.0	5.0	0.0	0.35V	PR(1 s)	10.068	1.009	0.998	0.897
36	70.0	0.0	25.0	0.0	5.0	0.0	0.35V	PR(1 s)	8.897	1.012	1.001	0.892
37	75.0	0.0	20.0	0.0	5.0	0.0	0.35V	PR(1 s)	7.785	1.013	0.997	0.872
38	85.0	0.0	15.0	0.0	0.0	0.0	0.35V	PR(1 s)	6.362	1.025	1.003	0.860
39	90.0	0.0	10.0	0.0	0.0	0.0	0.35V	PR(1 s)	5.245	1.085	1.045	0.858
40	95.0	0.0	5.0	0.0	0.0	0.0	0.35V	PR(1 s)	4.298	1.100	1.024	0.793
41	55.0	0.0	35.0	5.0	5.0	0.0	0.15V	PR(10 s)	1.153	0.934	0.813	0.595
42	60.0	0.0	30.0	5.0	5.0	0.0	0.15V	PR(10 s)	1.114	0.939	0.816	0.601
43	60.0	0.0	35.0	0.0	5.0	0.0	0.20V	PR(10 s)	1.588	0.991	0.938	0.783
44	65.0	0.0	30.0	0.0	5.0	0.0	0.20V	PR(10 s)	1.522	0.995	0.943	0.782
45	70.0	0.0	25.0	0.0	5.0	0.0	0.20V	PR(10 s)	1.400	0.999	0.948	0.781
46	75.0	0.0	20.0	0.0	5.0	0.0	0.20V	PR(10 s)	1.267	1.000	0.950	0.778
47	85.0	0.0	15.0	0.0	0.0	0.0	0.20V	PR(10 s)	0.902	0.993	0.959	0.840
48	90.0	0.0	10.0	0.0	0.0	0.0	0.20V	PR(10 s)	0.809	1.037	1.024	0.896
49	95.0	0.0	5.0	0.0	0.0	0.0	0.20V	PR(10 s)	0.667	1.054	1.058	0.854
50	60.0	0.0	35.0	0.0	5.0	0.0	0.25V	PR(10 s)	3.248	0.992	0.939	0.810
51	65.0	0.0	30.0	0.0	5.0	0.0	0.25V	PR(10 s)	3.078	0.994	0.943	0.816
52	70.0	0.0	25.0	0.0	5.0	0.0	0.25V	PR(10 s)	2.765	0.995	0.946	0.818
53	75.0	0.0	20.0	0.0	5.0	0.0	0.25V	PR(10 s)	2.436	0.994	0.947	0.815
54	45.0	0.0	40.0	5.0	10.0	0.0	0.25V	PR(10 s)	1.939	1.003	0.947	0.791
55	85.0	0.0	15.0	0.0	0.0	0.0	0.25V	PR(10 s)	1.694	0.979	0.964	0.853
56	90.0	0.0	10.0	0.0	0.0	0.0	0.25V	PR(10 s)	1.504	1.011	1.007	0.862
57	80.0	0.0	20.0	0.0	0.0	0.0	0.30V	PR(10 s)	6.308	0.952	0.915	0.804
58	60.0	0.0	35.0	0.0	5.0	0.0	0.30V	PR(10 s)	6.121	0.998	0.956	0.844
59	65.0	0.0	30.0	0.0	5.0	0.0	0.30V	PR(10 s)	5.777	1.001	0.962	0.853
60	70.0	0.0	25.0	0.0	5.0	0.0	0.30V	PR(10 s)	5.144	1.003	0.967	0.855
61	75.0	0.0	20.0	0.0	5.0	0.0	0.30V	PR(10 s)	4.501	1.004	0.969	0.849
62	55.0	0.0	30.0	5.0	10.0	0.0	0.30V	PR(10 s)	3.664	1.005	0.969	0.821
63	50.0	0.0	35.0	5.0	10.0	0.0	0.30V	PR(10 s)	3.599	1.008	0.971	0.826
64	85.0	0.0	15.0	0.0	0.0	0.0	0.30V	PR(10 s)	3.344	1.001	0.971	0.861
65	50.0	0.0	35.0	10.0	5.0	0.0	0.30V	PR(10 s)	3.025	1.004	0.971	0.836
66	90.0	0.0	10.0	0.0	0.0	0.0	0.30V	PR(10 s)	2.849	1.046	1.014	0.878
67	80.0	0.0	20.0	0.0	0.0	0.0	0.35V	PR(10 s)	12.413	0.956	0.934	0.807
68	60.0	0.0	35.0	0.0	5.0	0.0	0.35V	PR(10 s)	10.724	1.006	0.992	0.890
69	65.0	0.0	30.0	0.0	5.0	0.0	0.35V	PR(10 s)	10.068	1.009	0.998	0.897
70	70.0	0.0	25.0	0.0	5.0	0.0	0.35V	PR(10 s)	8.897	1.012	1.001	0.892
71	85.0	0.0	15.0	0.0	0.0	0.0	0.35V	PR(10 s)	6.362	1.025	1.003	0.860
72	90.0	0.0	10.0	0.0	0.0	0.0	0.35V	PR(10 s)	5.245	1.085	1.045	0.858
73	55.0	0.0	35.0	5.0	5.0	0.0	0.15V	PR(64 s)	1.153	0.934	0.813	0.595
74	60.0	0.0	30.0	5.0	5.0	0.0	0.15V	PR(64 s)	1.114	0.939	0.816	0.601
75	65.0	0.0	25.0	5.0	5.0	0.0	0.15V	PR(64 s)	1.017	0.940	0.815	0.605
76	70.0	0.0	20.0	5.0	5.0	0.0	0.15V	PR(64 s)	0.925	0.936	0.809	0.606
77	60.0	0.0	35.0	0.0	5.0	0.0	0.20V	PR(64 s)	1.588	0.991	0.938	0.783
78	55.0	0.0	40.0	0.0	5.0	0.0	0.20V	PR(64 s)	1.566	0.987	0.933	0.784
79	60.0	0.0	25.0	10.0	5.0	0.0	0.20V	PR(64 s)	1.054	1.004	0.943	0.785
80	55.0	0.0	30.0	10.0	5.0	0.0	0.20V	PR(64 s)	1.051	1.006	0.945	0.787
81	50.0	0.0	35.0	10.0	5.0	0.0	0.20V	PR(64 s)	1.029	1.006	0.945	0.788
82	45.0	0.0	40.0	10.0	5.0	0.0	0.20V	PR(64 s)	0.991	1.004	0.943	0.788
83	85.0	0.0	15.0	0.0	0.0	0.0	0.20V	PR(64 s)	0.902	0.993	0.959	0.840
84	90.0	0.0	10.0	0.0	0.0	0.0	0.20V	PR(64 s)	0.809	1.037	1.024	0.896
85	60.0	0.0	35.0	0.0	5.0	0.0	0.25V	PR(64 s)	3.248	0.992	0.939	0.810
86	65.0	0.0	30.0	0.0	5.0	0.0	0.25V	PR(64 s)	3.078	0.994	0.943	0.816
87	70.0	0.0	25.0	0.0	5.0	0.0	0.25V	PR(64 s)	2.765	0.995	0.946	0.818
88	85.0	0.0	15.0	0.0	0.0	0.0	0.25V	PR(64 s)	1.694	0.979	0.964	0.853
89	90.0	0.0	10.0	0.0	0.0	0.0	0.25V	PR(64 s)	1.504	1.011	1.007	0.862
90	80.0	0.0	20.0	0.0	0.0	0.0	0.30V	PR(64 s)	6.308	0.952	0.915	0.804
91	60.0	0.0	35.0	0.0	5.0	0.0	0.30V	PR(64 s)	6.121	0.998	0.956	0.844
92	65.0	0.0	30.0	0.0	5.0	0.0	0.30V	PR(64 s)	5.777	1.001	0.962	0.853
93	70.0	0.0	25.0	0.0	5.0	0.0	0.30V	PR(64 s)	5.144	1.003	0.967	0.855
94	85.0	0.0	15.0	0.0	0.0	0.0	0.30V	PR(64 s)	3.344	1.001	0.971	0.861
95	90.0	0.0	10.0	0.0	0.0	0.0	0.30V	PR(64 s)	2.849	1.046	1.014	0.878
96	50.0	0.0	25.0	10.0	15.0	0.0	0.30V	PR(64 s)	2.241	0.998	0.978	0.882
97	45.0	0.0	30.0	10.0	15.0	0.0	0.30V	PR(64 s)	2.192	1.007	0.985	0.886
98	80.0	0.0	20.0	0.0	0.0	0.0	0.35V	PR(64 s)	12.413	0.956	0.934	0.807
99	60.0	0.0	35.0	0.0	5.0	0.0	0.35V	PR(64 s)	10.724	1.006	0.992	0.890
100	65.0	0.0	30.0	0.0	5.0	0.0	0.35V	PR(64 s)	10.068	1.009	0.998	0.897
101	50.0	0.0	25.0	10.0	15.0	0.0	0.35V	PR(64 s)	4.255	1.008	1.010	0.897
102	45.0	0.0	30.0	10.0	15.0	0.0	0.35V	PR(64 s)	4.125	1.019	1.022	0.905
103	40.0	0.0	35.0	10.0	15.0	0.0	0.35V	PR(64 s)	3.933	1.022	1.026	0.909
104	35.0	0.0	35.0	10.0	20.0	0.0	0.35V	PR(64 s)	3.274	1.001	1.008	0.909

7. Appendix

7.1 Distribution of elements in the samples

This section summarizes the distribution of elemental compositions determined by SEM-EDX analysis and compares them with the nominal precursor electrolyte compositions. The data represent the complete dataset used to construct the averaged compositions presented in Figure S6.

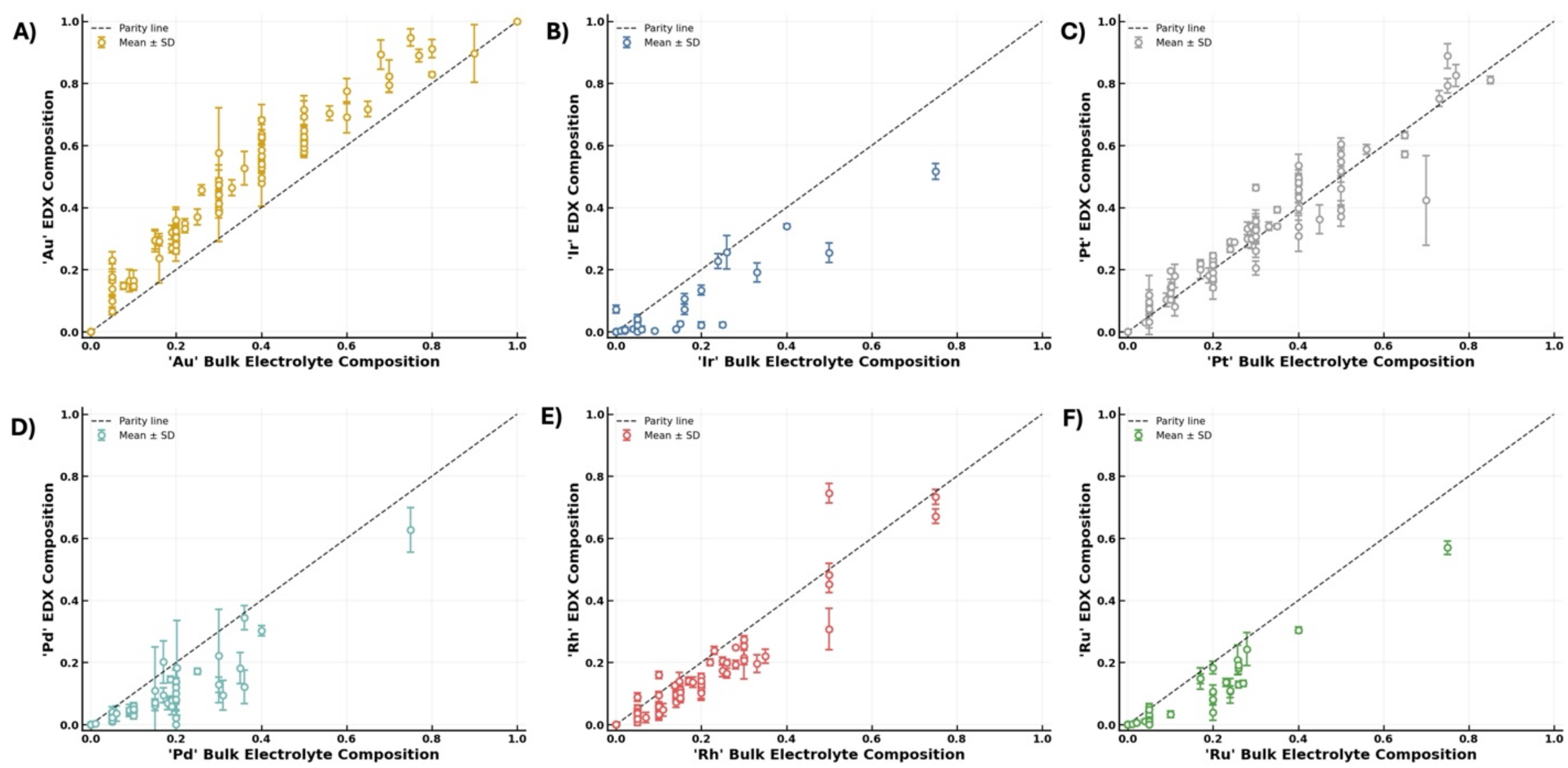

Figure S30: Compositional distribution of samples prepared using pulsed electrodeposition on glassy carbon (GC), measurements were performed using SEM-EDX. The same data were used to compute CLR variance and Aitchison variation ratio as shown in Figure S7.

7.2 Hyperspectral data: distribution of elements in the nanoparticles

Figures S31–S33 examine the compositional heterogeneity of a highly active catalyst $Au_{0.61}Pt_{0.34}Rh_{0.05}$ across multiple length scales. Hyperspectral S/TEM-EDX analysis combined with non-negative matrix factorization (NMF) reveals nanoscale variations in elemental distribution, whereas SEM-EDX provides the corresponding mesoscale average composition. The SEM-EDX measurements indicate a compositionally homogeneous catalyst on the mesoscale, while S/TEM-EDX reveals local Au/Pt segregation and nanoscale compositional fluctuations. These observations suggest that compositional heterogeneity contributes to the experimental variability captured by the Gaussian process surrogate models and may partly explain the uncertainty observed in regions of high catalytic activity.

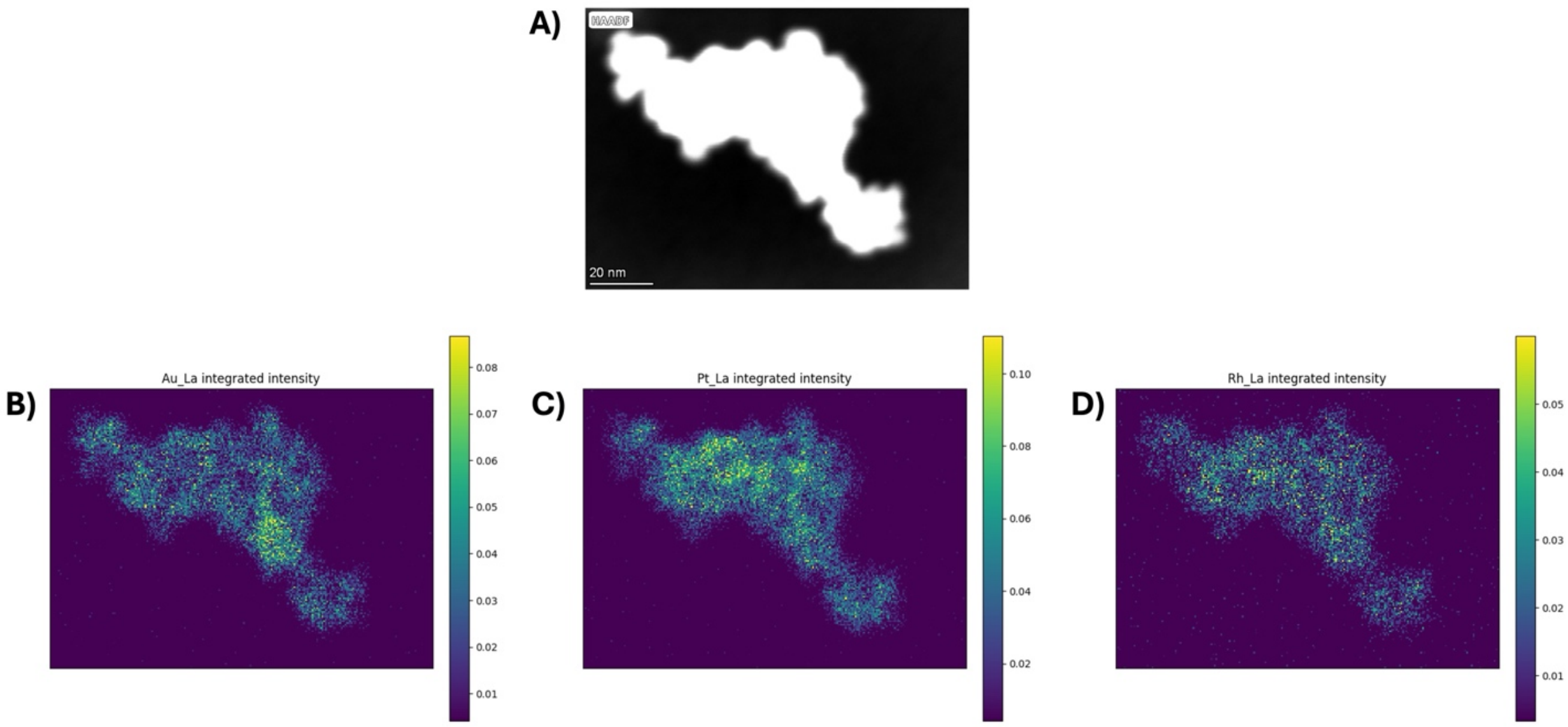

Figure S31: Representative hyperspectral S/TEM-EDX dataset of $Au_{0.61}Pt_{0.34}Rh_{0.05}$. A) STEM image of the analysed region. Elemental distribution maps for B) Au, C) Pt, and D) Rh are shown using 2 × 2 spatial binning.

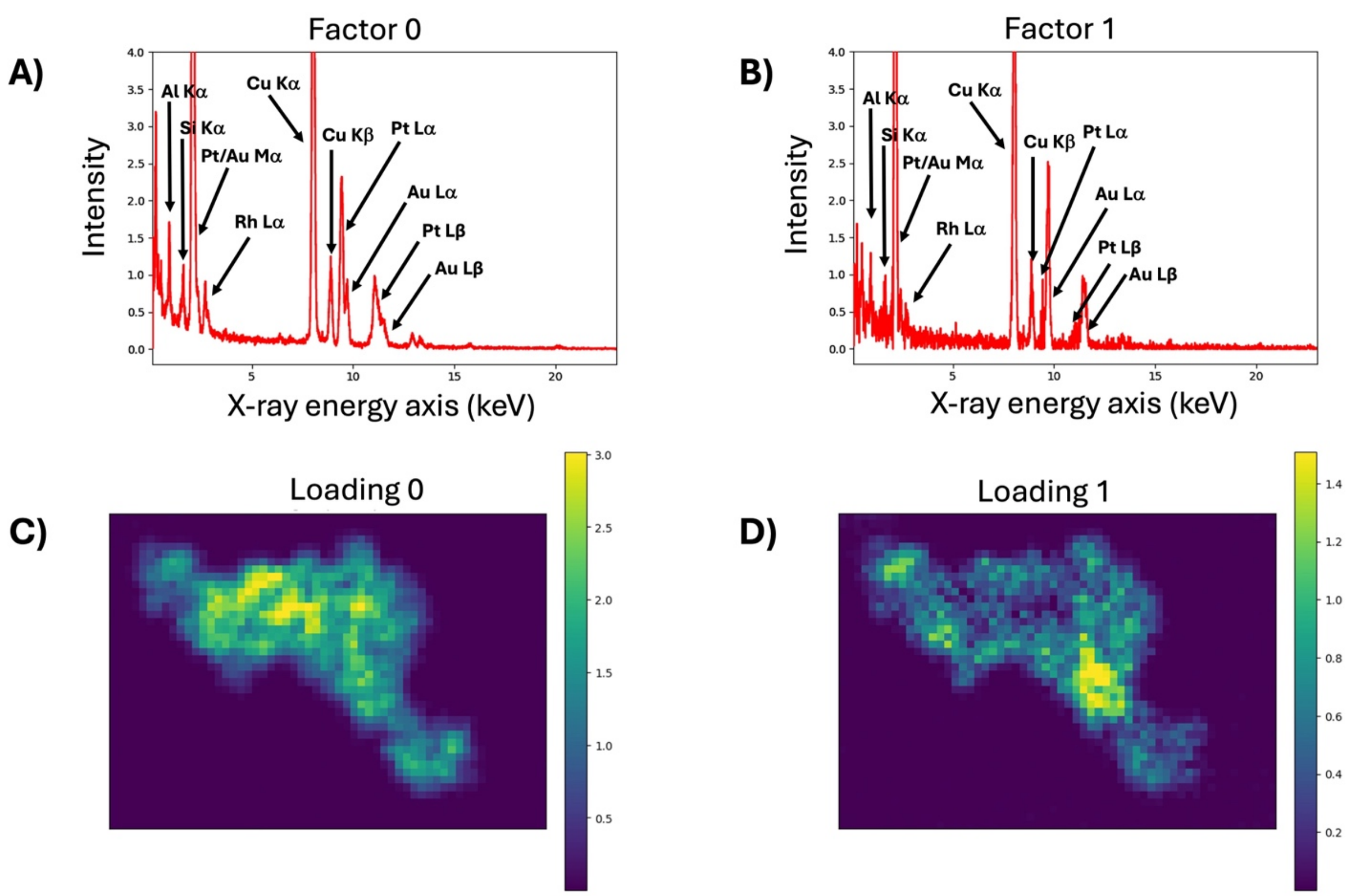

Figure S32: Non-negative matrix factorization (NMF) of the hyperspectral dataset shown in Figure S31 using 8 × 8 spatial binning (scale bar can be read by the raw file). The spectral factors and corresponding loading maps indicate partial mixing of Pt and Rh in Factor 0, whereas Au enrichment is predominantly captured by Factor 1, suggesting nanoscale Au segregation.

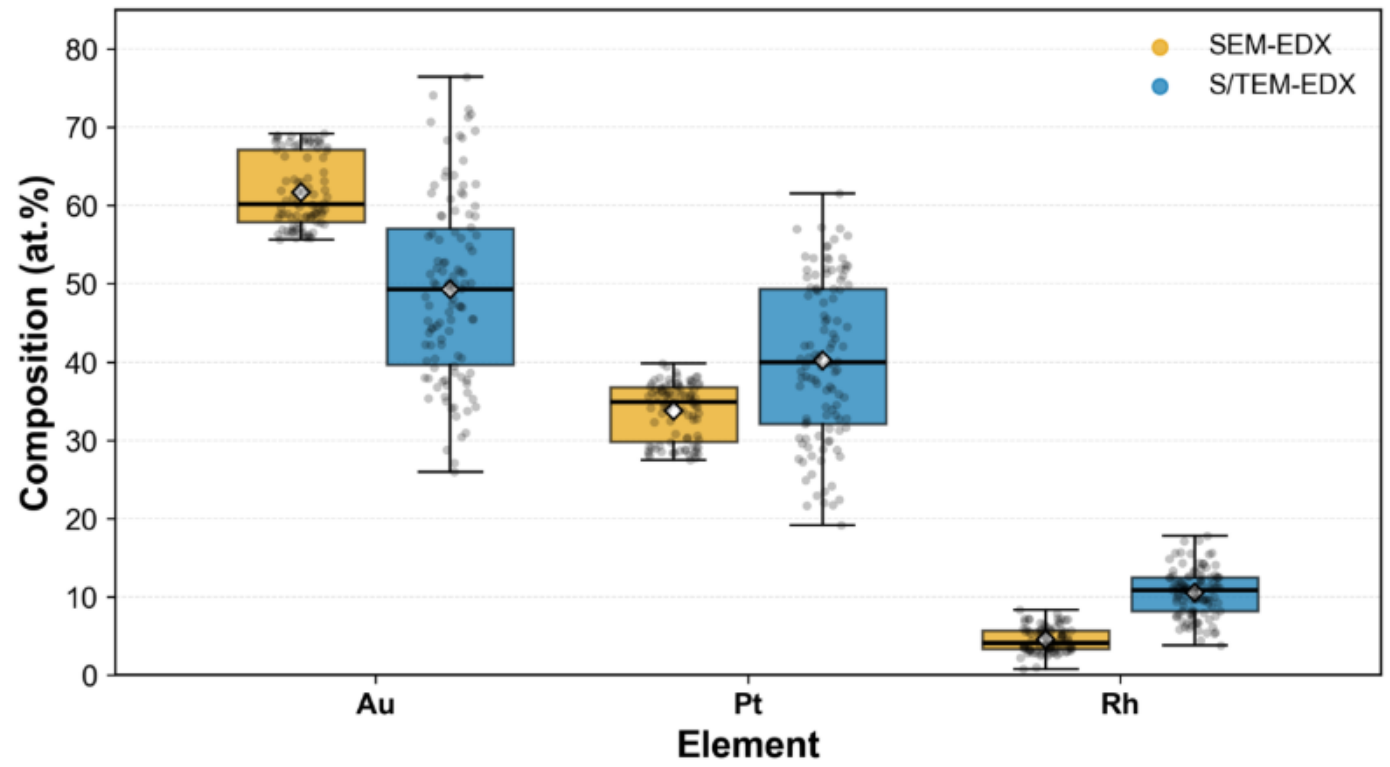

Figure S33: Statistical distribution of the elemental compositions in an active catalyst ($Au_{0.61}Pt_{0.34}Rh_{0.05}$) measured by SEM–EDX (mesoscale) and S/TEM–EDX (nanoscale). Data were acquired from the same sample across multiple regions, comprising 91 SEM–EDX and 110 S/TEM–EDX measurements.

7.3 Optimized Kernel

Table S2: Optimized Gaussian process regression covariance kernels and fitted hyperparameters. Length scales are reported in centered log-ratio (CLR)-transformed compositional space and therefore do not correspond directly to atomic percent. Predictive performance is reported as leave-one-group-out cross-validation (LOGO-CV) R^2.

objective	Objective label	potential	n_samples	R2_LOGO	y_log	optimized_kernel	length_scales	RQ_alpha	noise_level
Q_norm_mC_cm2	Q_{norm} (mC cm^{-2})	0.15V	210	0.612	TRUE	1.6**2 * RBF(length_scale=22.8) * RationalQuadratic(alpha=0.0434, length_scale=1.37) + WhiteKernel(noise_level=0.278)	22.8; 1.37	0.0434	0.278
Q_norm_mC_cm2	Q_{norm} (mC cm^{-2})	0.20V	213	0.613	TRUE	1.25**2 + Matern(length_scale=3.95, nu=1.5) * RationalQuadratic(alpha=0.0304, length_scale=0.079) + WhiteKernel(noise_level=0.132)	3.95; 0.079	0.0304	0.132
Q_norm_mC_cm2	Q_{norm} (mC cm^{-2})	0.25V	213	0.602	TRUE	1.4**2 + Matern(length_scale=5.55, nu=1.5) * RationalQuadratic(alpha=0.0442, length_scale=0.149) + WhiteKernel(noise_level=0.0852)	5.55; 0.149	0.0442	0.0852
Q_norm_mC_cm2	Q_{norm} (mC cm$^{-2})	0.30V	213	0.580	TRUE	1.45**2 + Matern(length_scale=6.16, nu=1.5) * RationalQuadratic(alpha=0.0482, length_scale=0.159) + WhiteKernel(noise_level=0.0649)	6.16; 0.159	0.0482	0.0649
Q_norm_mC_cm2	Q_{norm} (mC cm^{-2})	0.35V	213	0.549	TRUE	1.42**2 + Matern(length_scale=6.47, nu=1.5) * RationalQuadratic(alpha=0.051, length_scale=0.149) + WhiteKernel(noise_level=0.0543)	6.47; 0.149	0.051	0.0543
i_top1_norm	i(0.1 s) (mA cm^{-2})	0.15V	213	0.233	TRUE	1.52**2 * RationalQuadratic(alpha=0.0499, length_scale=0.414) + WhiteKernel(noise_level=0.308)	0.414	0.0499	0.308
i_top1_norm	i(0.1 s) (mA cm^{-2})	0.20V	213	0.637	TRUE	1.52**2 * Matern(length_scale=8.79, nu=1.5) * RationalQuadratic(alpha=0.0127, length_scale=0.0893) + WhiteKernel(noise_level=0.121)	8.79; 0.0893	0.0127	0.121
i_top1_norm	i(0.1 s) (mA cm^{-2})	0.25V	213	0.614	TRUE	1.46**2 + Matern(length_scale=5.43, nu=1.5) * RationalQuadratic(alpha=0.0386, length_scale=0.139) + WhiteKernel(noise_level=0.0837)	5.43; 0.139	0.0386	0.0837
i_top1_norm	i(0.1 s) (mA cm^{-2})	0.30V	213	0.600	TRUE	1.53**2 + Matern(length_scale=6.05, nu=1.5) * RationalQuadratic(alpha=0.043, length_scale=0.155) + WhiteKernel(noise_level=0.0641)	6.05; 0.155	0.043	0.0641

**i_top1_norm**	i(0.1 s) (mA cm^{-2})	0.35V	213	0.574	TRUE	1.61**2 + RBF(length_scale=5.62) + RationalQuadratic(alpha=0.0506, length_scale=0.157) + WhiteKernel(noise_level=0.0549)	5.62; 0.157	0.0506	0.0549
**poisoning_resistance_1sec**	PR(1 s)	0.15V	213	0.720	TRUE	1.86**2 * RationalQuadratic(alpha=0.151, length_scale=4.26) + WhiteKernel(noise_level=0.365)	4.26	0.151	0.365
**poisoning_resistance_1sec**	PR(1 s)	0.20V	213	0.671	TRUE	1.59**2 + RBF(length_scale=1.83) + Matern(length_scale=7.53, nu=1.5) + WhiteKernel(noise_level=0.198)	1.83; 7.53		0.198
**poisoning_resistance_1sec**	PR(1 s)	0.25V	213	0.725	TRUE	1.87**2 + RBF(length_scale=4.71) * RationalQuadratic(alpha=0.291, length_scale=2.67) + WhiteKernel(noise_level=0.149)	4.71; 2.67	0.291	0.149
**poisoning_resistance_1sec**	PR(1 s)	0.30V	213	0.739	TRUE	2.03**2 + RBF(length_scale=4.74) * RationalQuadratic(alpha=0.26, length_scale=2.77) + WhiteKernel(noise_level=0.117)	4.74; 2.77	0.26	0.117
**poisoning_resistance_1sec**	PR(1 s)	0.35V	213	0.683	TRUE	2.01**2 + RBF(length_scale=4.94) * RationalQuadratic(alpha=0.294, length_scale=2.35) + WhiteKernel(noise_level=0.104)	4.94; 2.35	0.294	0.104
**poisoning_resistance_10sec**	PR(10 s)	0.15V	213	0.189	TRUE	1.23**2 * RBF(length_scale=5) + WhiteKernel(noise_level=0.522)	5		0.522
**poisoning_resistance_10sec**	PR(10 s)	0.20V	213	0.615	TRUE	1.51**2 + RBF(length_scale=1.73) + Matern(length_scale=7.61, nu=1.5) + WhiteKernel(noise_level=0.231)	1.73; 7.61		0.231
**poisoning_resistance_10sec**	PR(10 s)	0.25V	213	0.702	TRUE	1.83**2 + RBF(length_scale=4.82) * RationalQuadratic(alpha=0.286, length_scale=2.37) + WhiteKernel(noise_level=0.152)	4.82; 2.37	0.286	0.152
**poisoning_resistance_10sec**	PR(10 s)	0.30V	213	0.723	TRUE	1.99**2 + RBF(length_scale=4.84) * RationalQuadratic(alpha=0.223, length_scale=2.47) + WhiteKernel(noise_level=0.129)	4.84; 2.47	0.223	0.129
**poisoning_resistance_10sec**	PR(10 s)	0.35V	213	0.660	TRUE	1.92**2 + RBF(length_scale=5) * RationalQuadratic(alpha=0.269, length_scale=1.94) + WhiteKernel(noise_level=0.107)	5; 1.94	0.269	0.107
**poisoning_resistance_64sec**	PR(64 s)	0.15V	210	0.595	TRUE	RBF(length_scale=4.39) + WhiteKernel(noise_level=0.597)	4.39		0.597
**poisoning_resistance_64sec**	PR(64 s)	0.20V	213	0.390	TRUE	1.14**2 + RBF(length_scale=5.84) + RationalQuadratic(alpha=1e+05, length_scale=1.16) + WhiteKernel(noise_level=0.318)	5.84; 1.16	100000.0	0.318
**poisoning_resistance_64sec**	PR(64 s)	0.25V	213	0.566	TRUE	1.52**2 + RBF(length_scale=4.82) * RationalQuadratic(alpha=0.306, length_scale=1.42) + WhiteKernel(noise_level=0.166)	4.82; 1.42	0.306	0.166
**poisoning_resistance_64sec**	PR(64 s)	0.30V	213	0.641	TRUE	1.99**2 + RBF(length_scale=5.47) + RationalQuadratic(alpha=0.49, length_scale=1.47) + WhiteKernel(noise_level=0.118)	5.47; 1.47	0.49	0.118
**poisoning_resistance_64sec**	PR(64 s)	0.35V	213	0.606	TRUE	1.84**2 + Matern(length_scale=7.53, nu=1.5) + RationalQuadratic(alpha=0.482, length_scale=1.34) + WhiteKernel(noise_level=0.106)	7.53; 1.34	0.482	0.106

7.4 Measured Charge as an 'activity' objective

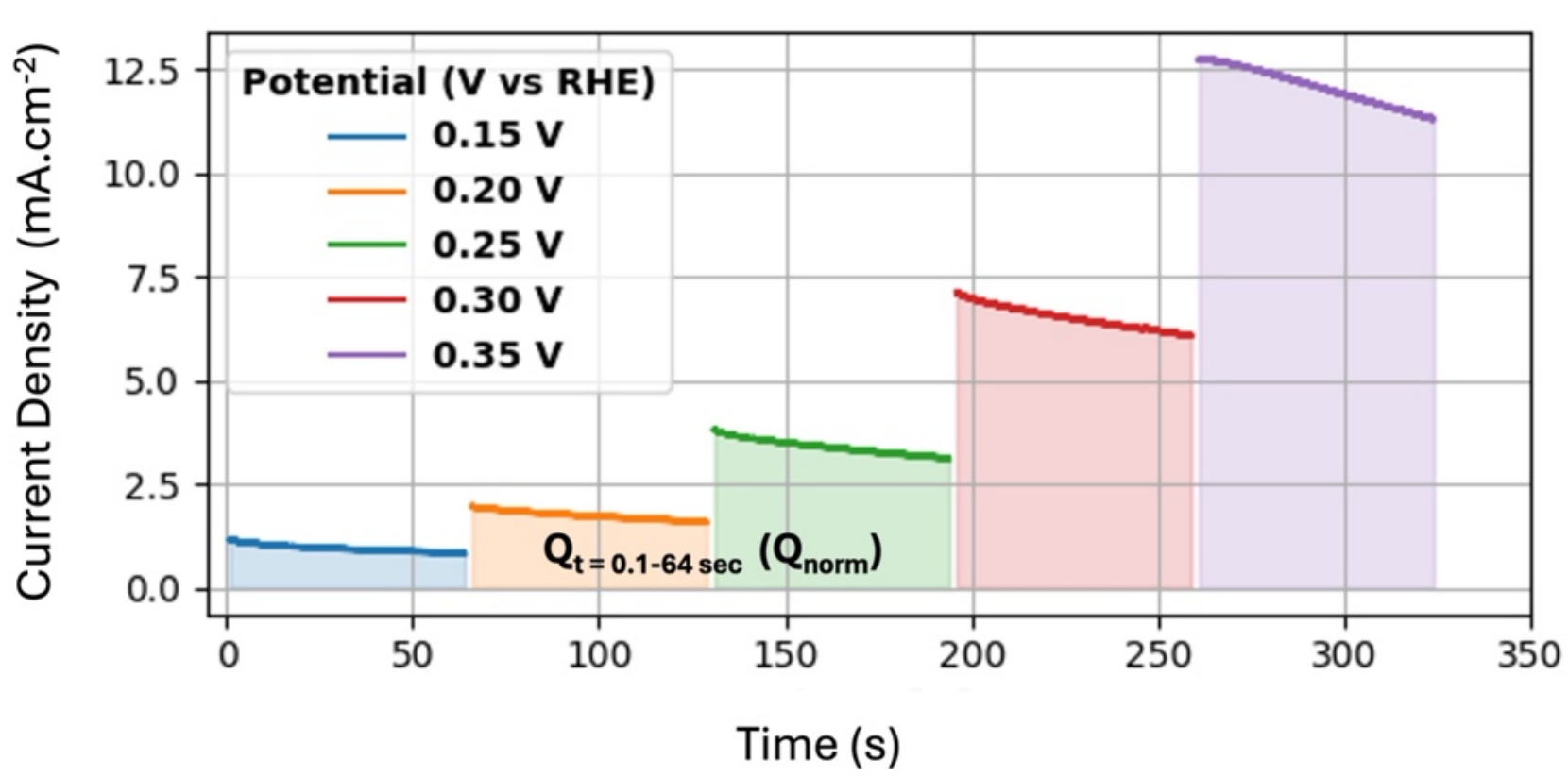

Figure S34: Nanoparticle coverage normalized integrated charge (Q_{norm}), calculated by integrating the current response between 0.1 and 64 s, used as an alternative activity descriptor.

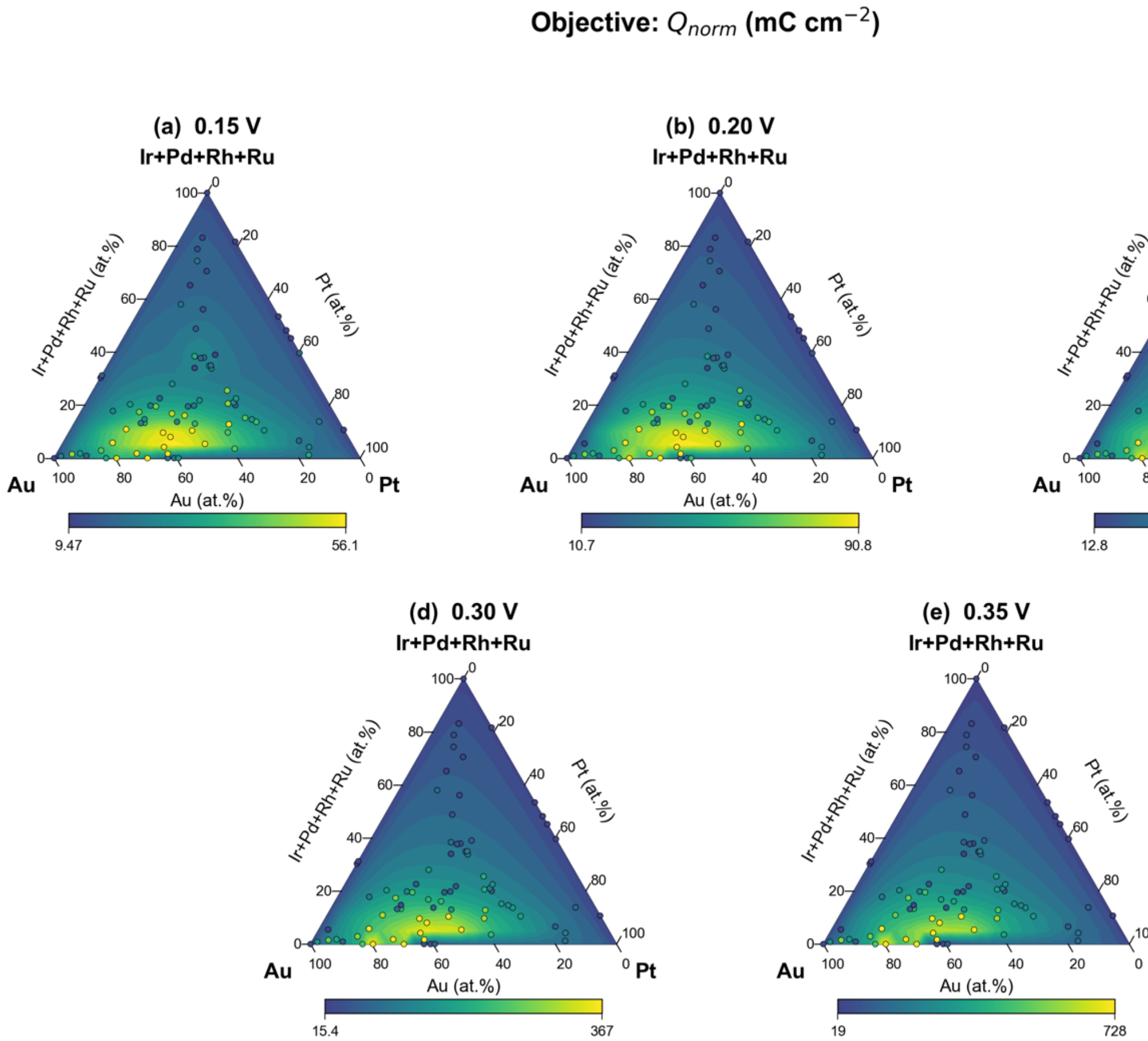

Figure S35: Gaussian process regression (GPR) surrogate maps of the Au–Ir–Pt–Pd–Rh–Ru composition–activity landscape using the normalized integrated charge (Q_{norm}) as the activity descriptor. The integrated charge was calculated over the time interval 0.1–64 s.

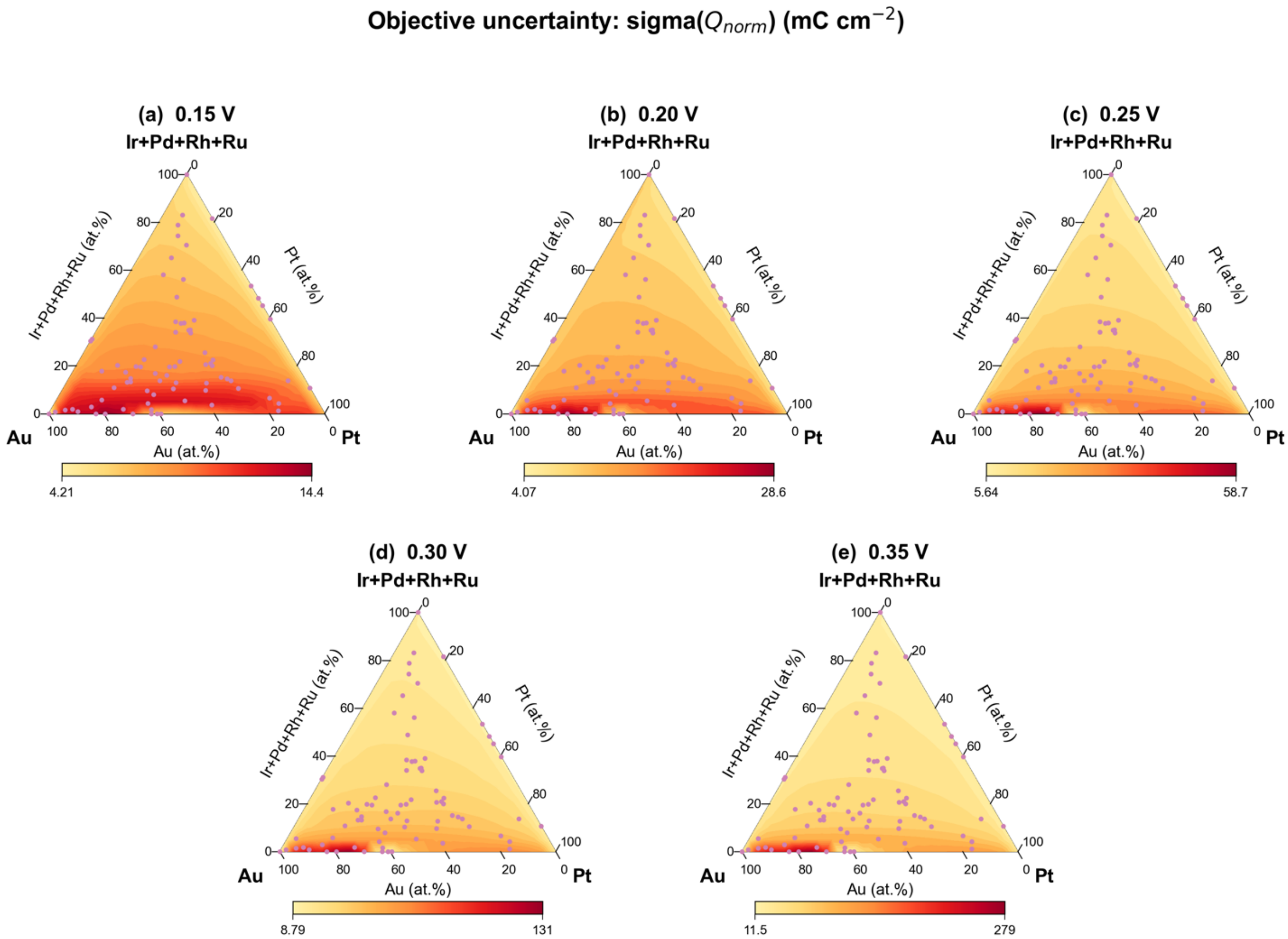

Figure S36: Gaussian process regression (GPR) uncertainty maps showing the predicted absolute uncertainty of the normalized integrated charge (Q_{norm}) across the Au–Ir–Pt–Pd–Rh–Ru compositional space.

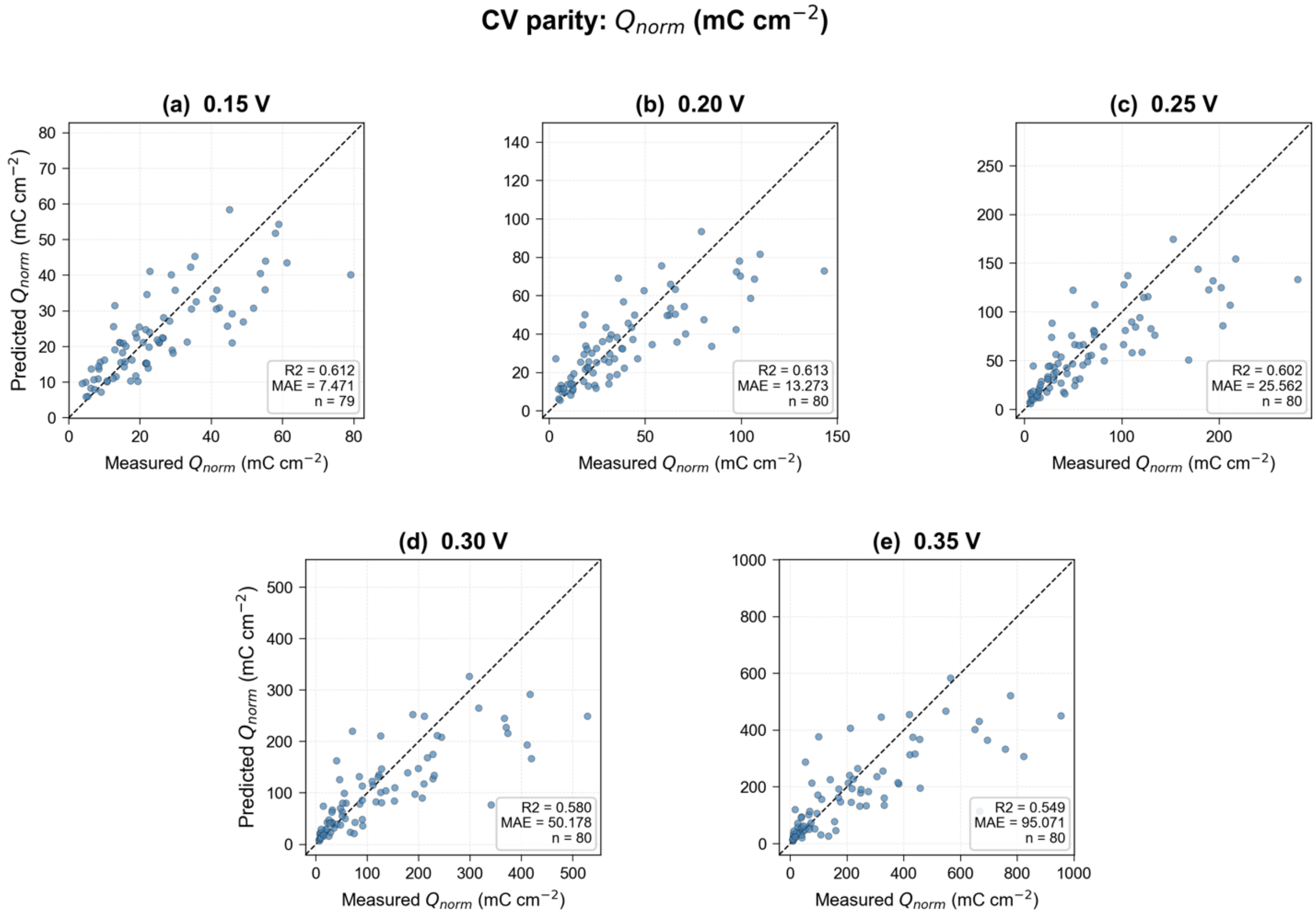

Figure S37: Leave-one-group-out cross-validation (LOGO-CV) parity plots evaluating the predictive performance of the Gaussian process regression surrogate models for the normalized integrated charge (Q_{norm}).

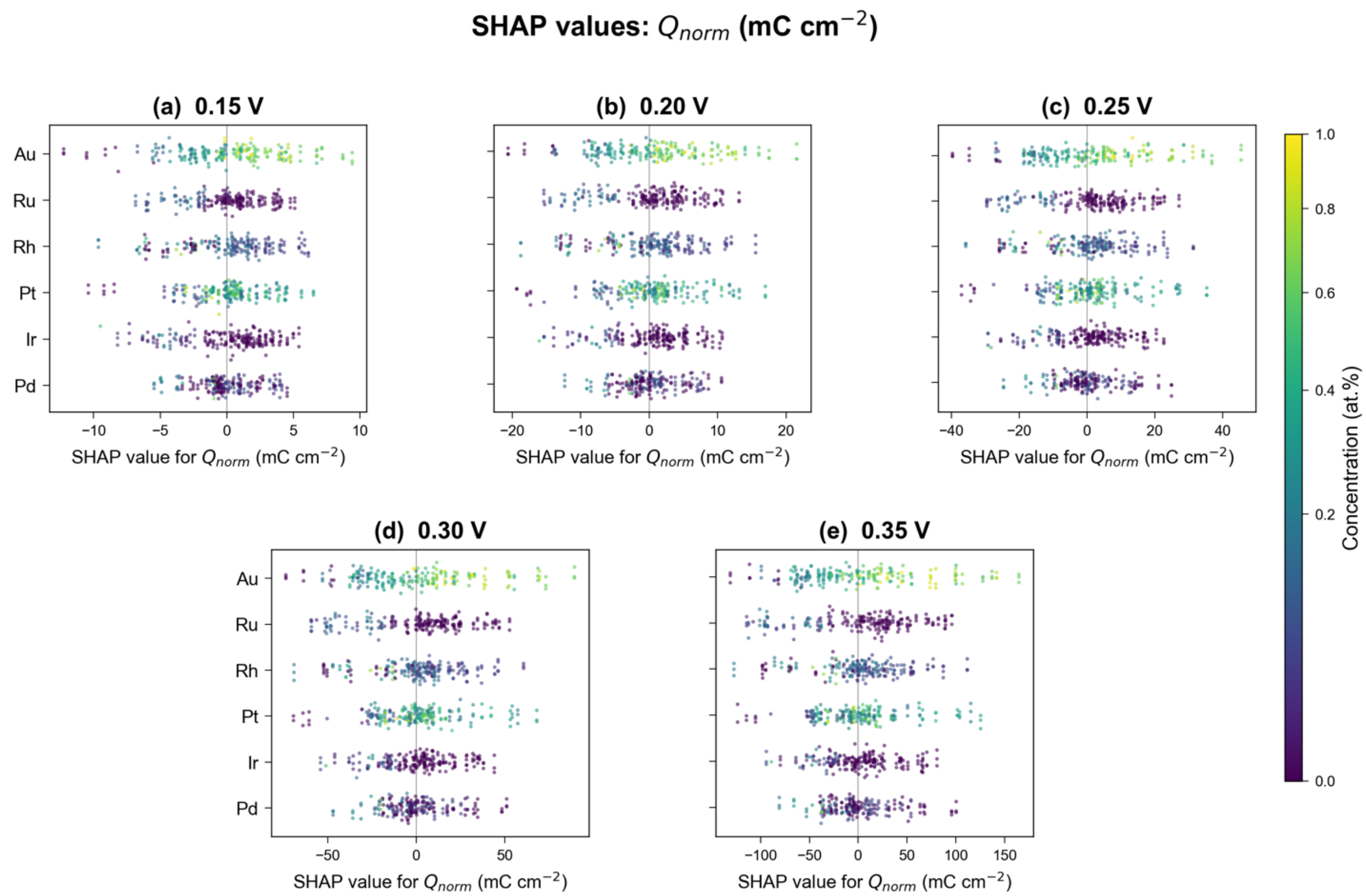

Figure S38: SHAP (Shapley additive explanations) summary plots illustrating the contributions of the six elemental descriptors to the Gaussian process regression surrogate models for the normalized integrated charge (Q_{norm}).

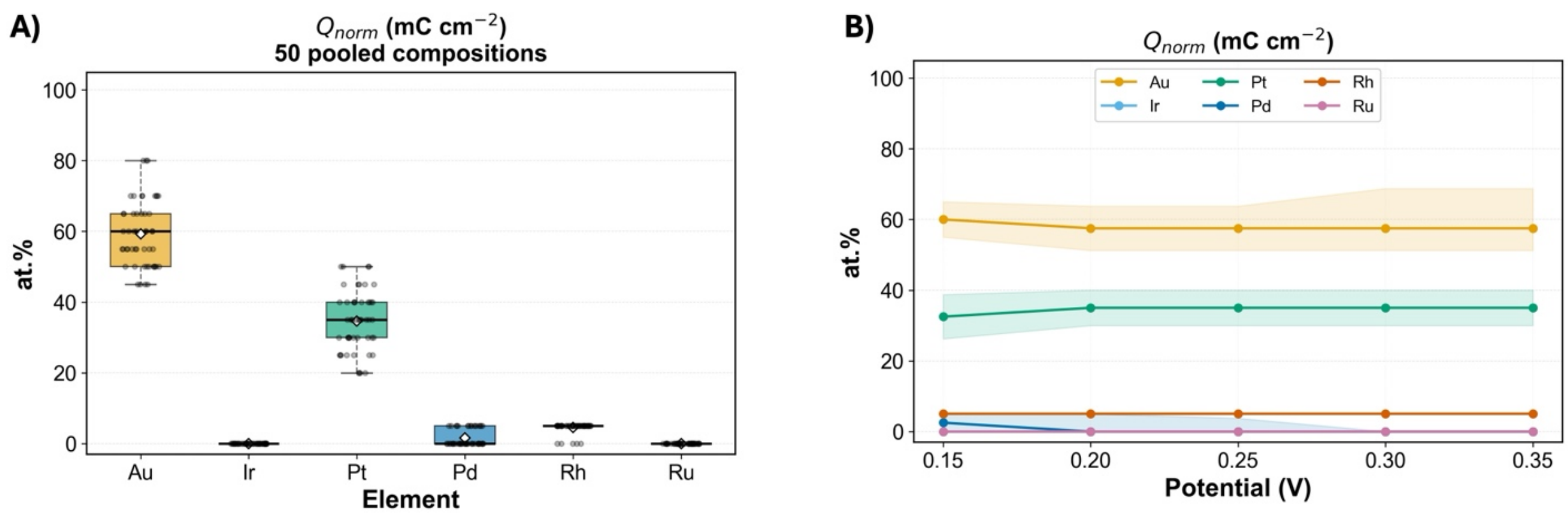

Figure S39: A) Elemental compositions found as Q_{norm} being the objective for the 50 highest-ranked candidate compositions pooled across all applied potentials. B) Potential-dependent elemental compositions of the top Gaussian process regression (GPR)-predicted candidates based on the Q_{norm} activity descriptor.

7.5 *Multi-objective analysis for Q_{norm}*

Table S3: Pareto-optimal catalyst compositions corresponding to Figure S40 using the normalized integrated charge (Q_{norm}) as the activity descriptor.

ID	Au	Ir	Pt	Pd	Rh	Ru	Potential	Pareto vs	GPR Q	GPR PR1	GPR PR10	GPR PR64
1	60.0	0.0	35.0	0.0	5.0	0.0	0.15V	PR(1 s)	56.053	0.927	0.793	0.566
2	65.0	0.0	30.0	0.0	5.0	0.0	0.15V	PR(1 s)	54.393	0.929	0.788	0.571
3	55.0	0.0	35.0	5.0	5.0	0.0	0.15V	PR(1 s)	53.495	0.934	0.813	0.595
4	60.0	0.0	30.0	5.0	5.0	0.0	0.15V	PR(1 s)	53.199	0.939	0.816	0.601
5	65.0	0.0	25.0	5.0	5.0	0.0	0.15V	PR(1 s)	50.459	0.940	0.815	0.605
6	60.0	0.0	35.0	0.0	5.0	0.0	0.20V	PR(1 s)	90.819	0.991	0.938	0.783
7	65.0	0.0	30.0	0.0	5.0	0.0	0.20V	PR(1 s)	87.448	0.995	0.943	0.782
8	70.0	0.0	25.0	0.0	5.0	0.0	0.20V	PR(1 s)	80.448	0.999	0.948	0.781
9	75.0	0.0	20.0	0.0	5.0	0.0	0.20V	PR(1 s)	72.095	1.000	0.950	0.778
10	60.0	0.0	25.0	10.0	5.0	0.0	0.20V	PR(1 s)	57.313	1.004	0.943	0.785
11	55.0	0.0	30.0	10.0	5.0	0.0	0.20V	PR(1 s)	57.033	1.006	0.945	0.787
12	55.0	0.0	25.0	15.0	5.0	0.0	0.20V	PR(1 s)	45.967	1.009	0.945	0.783
13	90.0	0.0	10.0	0.0	0.0	0.0	0.20V	PR(1 s)	45.266	1.037	1.024	0.896
14	95.0	0.0	5.0	0.0	0.0	0.0	0.20V	PR(1 s)	36.955	1.054	1.058	0.854
15	60.0	0.0	35.0	0.0	5.0	0.0	0.25V	PR(1 s)	182.470	0.992	0.939	0.810
16	65.0	0.0	30.0	0.0	5.0	0.0	0.25V	PR(1 s)	172.366	0.994	0.943	0.816
17	55.0	0.0	35.0	5.0	5.0	0.0	0.25V	PR(1 s)	156.061	0.994	0.938	0.774
18	70.0	0.0	25.0	0.0	5.0	0.0	0.25V	PR(1 s)	153.649	0.995	0.946	0.818
19	55.0	0.0	30.0	5.0	10.0	0.0	0.25V	PR(1 s)	113.518	0.999	0.943	0.778
20	50.0	0.0	35.0	5.0	10.0	0.0	0.25V	PR(1 s)	111.426	1.002	0.947	0.784
21	45.0	0.0	40.0	5.0	10.0	0.0	0.25V	PR(1 s)	106.338	1.003	0.947	0.791
22	90.0	0.0	10.0	0.0	0.0	0.0	0.25V	PR(1 s)	83.904	1.011	1.007	0.862
23	40.0	5.0	40.0	5.0	5.0	5.0	0.25V	PR(1 s)	30.763	1.020	0.898	0.619
24	80.0	0.0	20.0	0.0	0.0	0.0	0.30V	PR(1 s)	367.022	0.952	0.915	0.804
25	60.0	0.0	35.0	0.0	5.0	0.0	0.30V	PR(1 s)	351.894	0.998	0.956	0.844
26	65.0	0.0	30.0	0.0	5.0	0.0	0.30V	PR(1 s)	331.027	1.001	0.962	0.853
27	70.0	0.0	25.0	0.0	5.0	0.0	0.30V	PR(1 s)	292.099	1.003	0.967	0.855
28	75.0	0.0	20.0	0.0	5.0	0.0	0.30V	PR(1 s)	253.222	1.004	0.969	0.849
29	55.0	0.0	30.0	5.0	10.0	0.0	0.30V	PR(1 s)	209.125	1.005	0.969	0.821
30	50.0	0.0	35.0	5.0	10.0	0.0	0.30V	PR(1 s)	205.437	1.008	0.971	0.826
31	45.0	0.0	40.0	5.0	10.0	0.0	0.30V	PR(1 s)	196.080	1.008	0.970	0.833
32	50.0	0.0	30.0	10.0	10.0	0.0	0.30V	PR(1 s)	160.445	1.012	0.986	0.869
33	90.0	0.0	10.0	0.0	0.0	0.0	0.30V	PR(1 s)	157.744	1.046	1.014	0.878
34	95.0	0.0	5.0	0.0	0.0	0.0	0.30V	PR(1 s)	126.524	1.056	1.009	0.825
35	80.0	0.0	20.0	0.0	0.0	0.0	0.35V	PR(1 s)	727.953	0.956	0.934	0.807
36	60.0	0.0	35.0	0.0	5.0	0.0	0.35V	PR(1 s)	640.945	1.006	0.992	0.890
37	65.0	0.0	30.0	0.0	5.0	0.0	0.35V	PR(1 s)	600.764	1.009	0.998	0.897
38	70.0	0.0	25.0	0.0	5.0	0.0	0.35V	PR(1 s)	525.540	1.012	1.001	0.892
39	75.0	0.0	20.0	0.0	5.0	0.0	0.35V	PR(1 s)	452.046	1.013	0.997	0.872
40	50.0	0.0	35.0	5.0	10.0	0.0	0.35V	PR(1 s)	370.204	1.014	0.994	0.842
41	85.0	0.0	15.0	0.0	0.0	0.0	0.35V	PR(1 s)	359.309	1.025	1.003	0.860
42	90.0	0.0	10.0	0.0	0.0	0.0	0.35V	PR(1 s)	286.959	1.085	1.045	0.858
43	95.0	0.0	5.0	0.0	0.0	0.0	0.35V	PR(1 s)	226.693	1.100	1.024	0.793
44	60.0	0.0	35.0	0.0	5.0	0.0	0.15V	PR(10 s)	56.053	0.927	0.793	0.566
45	55.0	0.0	40.0	0.0	5.0	0.0	0.15V	PR(10 s)	54.949	0.922	0.794	0.560
46	55.0	0.0	35.0	5.0	5.0	0.0	0.15V	PR(10 s)	53.495	0.934	0.813	0.595
47	60.0	0.0	30.0	5.0	5.0	0.0	0.15V	PR(10 s)	53.199	0.939	0.816	0.601
48	60.0	0.0	35.0	0.0	5.0	0.0	0.20V	PR(10 s)	90.819	0.991	0.938	0.783
49	65.0	0.0	30.0	0.0	5.0	0.0	0.20V	PR(10 s)	87.448	0.995	0.943	0.782
50	70.0	0.0	25.0	0.0	5.0	0.0	0.20V	PR(10 s)	80.448	0.999	0.948	0.781
51	75.0	0.0	20.0	0.0	5.0	0.0	0.20V	PR(10 s)	72.095	1.000	0.950	0.778
52	85.0	0.0	15.0	0.0	0.0	0.0	0.20V	PR(10 s)	50.480	0.993	0.959	0.840
53	90.0	0.0	10.0	0.0	0.0	0.0	0.20V	PR(10 s)	45.266	1.037	1.024	0.896
54	95.0	0.0	5.0	0.0	0.0	0.0	0.20V	PR(10 s)	36.955	1.054	1.058	0.854
55	60.0	0.0	35.0	0.0	5.0	0.0	0.25V	PR(10 s)	182.470	0.992	0.939	0.810
56	65.0	0.0	30.0	0.0	5.0	0.0	0.25V	PR(10 s)	172.366	0.994	0.943	0.816
57	70.0	0.0	25.0	0.0	5.0	0.0	0.25V	PR(10 s)	153.649	0.995	0.946	0.818
58	75.0	0.0	20.0	0.0	5.0	0.0	0.25V	PR(10 s)	134.439	0.994	0.947	0.815
59	45.0	0.0	40.0	5.0	10.0	0.0	0.25V	PR(10 s)	106.338	1.003	0.947	0.791
60	85.0	0.0	15.0	0.0	0.0	0.0	0.25V	PR(10 s)	96.894	0.979	0.964	0.853
61	90.0	0.0	10.0	0.0	0.0	0.0	0.25V	PR(10 s)	83.904	1.011	1.007	0.862
62	80.0	0.0	20.0	0.0	0.0	0.0	0.30V	PR(10 s)	367.022	0.952	0.915	0.804
63	60.0	0.0	35.0	0.0	5.0	0.0	0.30V	PR(10 s)	351.894	0.998	0.956	0.844
64	65.0	0.0	30.0	0.0	5.0	0.0	0.30V	PR(10 s)	331.027	1.001	0.962	0.853
65	70.0	0.0	25.0	0.0	5.0	0.0	0.30V	PR(10 s)	292.099	1.003	0.967	0.855
66	75.0	0.0	20.0	0.0	5.0	0.0	0.30V	PR(10 s)	253.222	1.004	0.969	0.849
67	55.0	0.0	30.0	5.0	10.0	0.0	0.30V	PR(10 s)	209.125	1.005	0.969	0.821
68	50.0	0.0	35.0	5.0	10.0	0.0	0.30V	PR(10 s)	205.437	1.008	0.971	0.826
69	85.0	0.0	15.0	0.0	0.0	0.0	0.30V	PR(10 s)	189.547	1.001	0.971	0.861
70	50.0	0.0	35.0	10.0	5.0	0.0	0.30V	PR(10 s)	172.863	1.004	0.971	0.836
71	55.0	0.0	25.0	10.0	10.0	0.0	0.30V	PR(10 s)	165.263	1.004	0.980	0.868
72	50.0	0.0	30.0	10.0	10.0	0.0	0.30V	PR(10 s)	160.445	1.012	0.986	0.869
73	90.0	0.0	10.0	0.0	0.0	0.0	0.30V	PR(10 s)	157.744	1.046	1.014	0.878
74	80.0	0.0	20.0	0.0	0.0	0.0	0.35V	PR(10 s)	727.953	0.956	0.934	0.807
75	60.0	0.0	35.0	0.0	5.0	0.0	0.35V	PR(10 s)	640.945	1.006	0.992	0.890
76	65.0	0.0	30.0	0.0	5.0	0.0	0.35V	PR(10 s)	600.764	1.009	0.998	0.897
77	70.0	0.0	25.0	0.0	5.0	0.0	0.35V	PR(10 s)	525.540	1.012	1.001	0.892
78	85.0	0.0	15.0	0.0	0.0	0.0	0.35V	PR(10 s)	359.309	1.025	1.003	0.860
79	55.0	0.0	25.0	10.0	10.0	0.0	0.35V	PR(10 s)	309.747	1.013	1.008	0.885
80	50.0	0.0	30.0	10.0	10.0	0.0	0.35V	PR(10 s)	300.271	1.023	1.019	0.890
81	90.0	0.0	10.0	0.0	0.0	0.0	0.35V	PR(10 s)	286.959	1.085	1.045	0.858
82	60.0	0.0	35.0	0.0	5.0	0.0	0.15V	PR(64 s)	56.053	0.927	0.793	0.566
83	65.0	0.0	30.0	0.0	5.0	0.0	0.15V	PR(64 s)	54.393	0.929	0.788	0.571
84	55.0	0.0	35.0	5.0	5.0	0.0	0.15V	PR(64 s)	53.495	0.934	0.813	0.595
85	60.0	0.0	30.0	5.0	5.0	0.0	0.15V	PR(64 s)	53.199	0.939	0.816	0.601
86	65.0	0.0	25.0	5.0	5.0	0.0	0.15V	PR(64 s)	50.459	0.940	0.815	0.605
87	70.0	0.0	20.0	5.0	5.0	0.0	0.15V	PR(64 s)	46.254	0.936	0.809	0.606
88	60.0	0.0	35.0	0.0	5.0	0.0	0.20V	PR(64 s)	90.819	0.991	0.938	0.783
89	55.0	0.0	40.0	0.0	5.0	0.0	0.20V	PR(64 s)	88.996	0.987	0.933	0.784
90	60.0	0.0	25.0	10.0	5.0	0.0	0.20V	PR(64 s)	57.313	1.004	0.943	0.785
91	55.0	0.0	30.0	10.0	5.0	0.0	0.20V	PR(64 s)	57.033	1.006	0.945	0.787
92	50.0	0.0	35.0	10.0	5.0	0.0	0.20V	PR(64 s)	55.548	1.006	0.945	0.788
93	45.0	0.0	40.0	10.0	5.0	0.0	0.20V	PR(64 s)	53.119	1.004	0.943	0.788
94	85.0	0.0	15.0	0.0	0.0	0.0	0.20V	PR(64 s)	50.480	0.993	0.959	0.840
95	90.0	0.0	10.0	0.0	0.0	0.0	0.20V	PR(64 s)	45.266	1.037	1.024	0.896
96	60.0	0.0	35.0	0.0	5.0	0.0	0.25V	PR(64 s)	182.470	0.992	0.939	0.810
97	65.0	0.0	30.0	0.0	5.0	0.0	0.25V	PR(64 s)	172.366	0.994	0.943	0.816
98	70.0	0.0	25.0	0.0	5.0	0.0	0.25V	PR(64 s)	153.649	0.995	0.946	0.818
99	85.0	0.0	15.0	0.0	0.0	0.0	0.25V	PR(64 s)	96.894	0.979	0.964	0.853
100	90.0	0.0	10.0	0.0	0.0	0.0	0.25V	PR(64 s)	83.904	1.011	1.007	0.862
101	80.0	0.0	20.0	0.0	0.0	0.0	0.30V	PR(64 s)	367.022	0.952	0.915	0.804
102	60.0	0.0	35.0	0.0	5.0	0.0	0.30V	PR(64 s)	351.894	0.998	0.956	0.844
103	65.0	0.0	30.0	0.0	5.0	0.0	0.30V	PR(64 s)	331.027	1.001	0.962	0.853
104	70.0	0.0	25.0	0.0	5.0	0.0	0.30V	PR(64 s)	292.099	1.003	0.967	0.855
105	85.0	0.0	15.0	0.0	0.0	0.0	0.30V	PR(64 s)	189.547	1.001	0.971	0.861
106	55.0	0.0	25.0	10.0	10.0	0.0	0.30V	PR(64 s)	165.263	1.004	0.980	0.868
107	50.0	0.0	30.0	10.0	10.0	0.0	0.30V	PR(64 s)	160.445	1.012	0.986	0.869
108	90.0	0.0	10.0	0.0	0.0	0.0	0.30V	PR(64 s)	157.744	1.046	1.014	0.878
109	50.0	0.0	25.0	10.0	15.0	0.0	0.30V	PR(64 s)	132.266	0.998	0.978	0.882
110	45.0	0.0	30.0	10.0	15.0	0.0	0.30V	PR(64 s)	128.692	1.007	0.985	0.886
111	80.0	0.0	20.0	0.0	0.0	0.0	0.35V	PR(64 s)	727.953	0.956	0.934	0.807
112	60.0	0.0	35.0	0.0	5.0	0.0	0.35V	PR(64 s)	640.945	1.006	0.992	0.890
113	65.0	0.0	30.0	0.0	5.0	0.0	0.35V	PR(64 s)	600.764	1.009	0.998	0.897
114	50.0	0.0	25.0	10.0	15.0	0.0	0.35V	PR(64 s)	255.384	1.008	1.010	0.897
115	45.0	0.0	30.0	10.0	15.0	0.0	0.35V	PR(64 s)	247.872	1.019	1.022	0.905
116	40.0	0.0	35.0	10.0	15.0	0.0	0.35V	PR(64 s)	235.695	1.022	1.026	0.909
117	35.0	0.0	35.0	10.0	20.0	0.0	0.35V	PR(64 s)	195.415	1.001	1.008	0.909

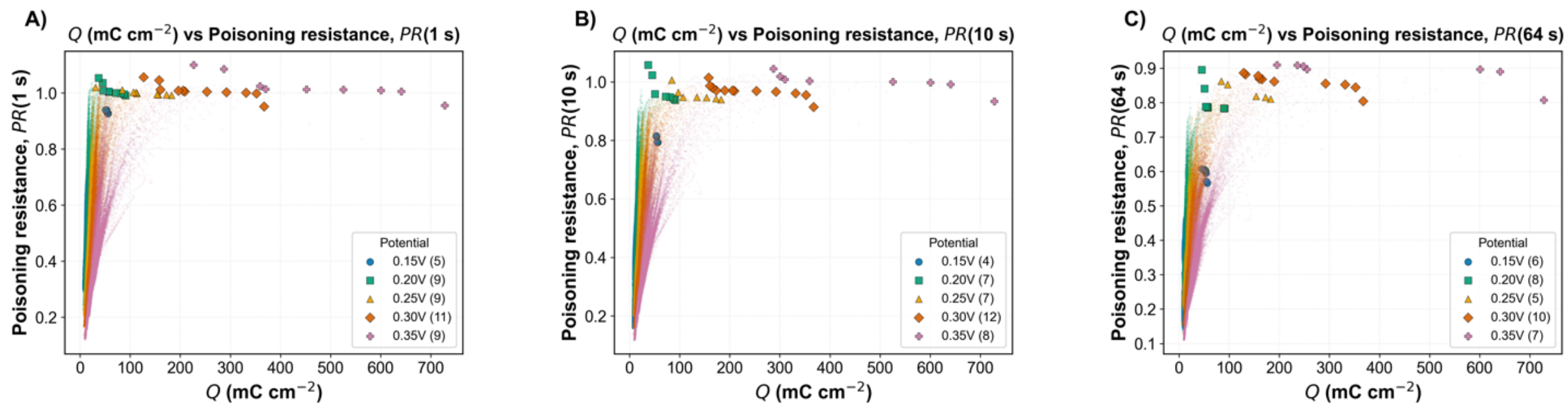

Figure S40: Pareto front for the trade-off between activity (Q_{norm}) and poisoning resistance at all the potentials. Small points represent Gaussian process regression (GPR) predictions evaluated on the Au–Ir–Pt–Pd–Rh–Ru compositional grid (5 at. % spacing), whereas the markers denote the corresponding Pareto-optimal compositions identified by the bi-objective analysis.

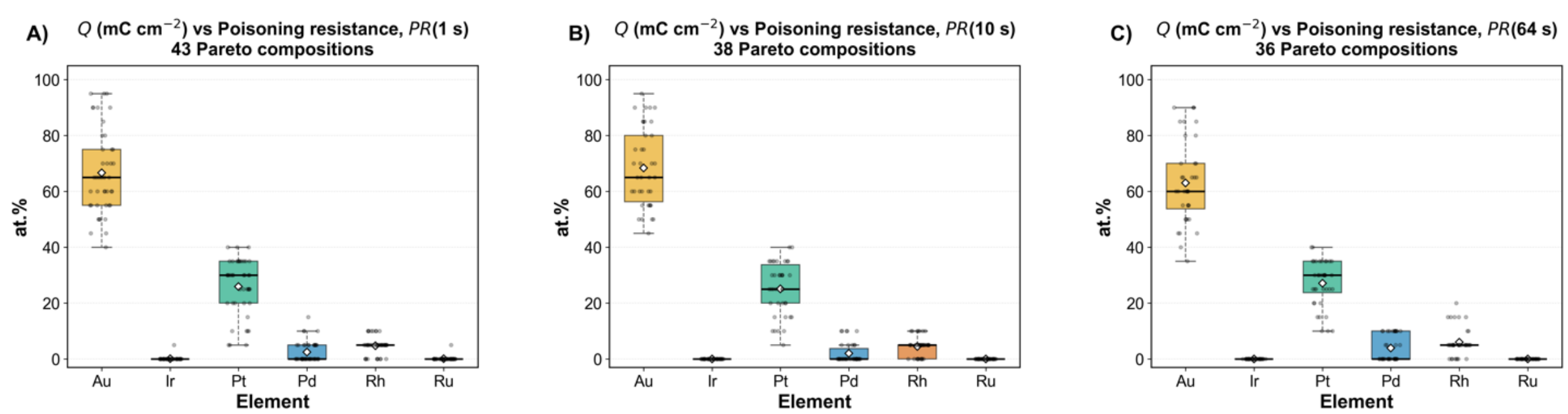

Figure S41: Elemental composition distributions of the Pareto-optimal catalyst compositions identified in Figure S40. The distribution pool (union subset) the pareto optimal entries across all the five potentials.

References

1. Gautam D., Wiberg G. K. H., Quinson J., Wang D., Clausen C. M., Rohde R., Klemmt R., Rossmeisl J., Bøjesen E. D., Arenz M., Small Struct. 2026,7, 1, e202500666.
